# Association of polygenic scores for depression and neuroticism with perceived stress in daily life during a long‐lasting stress period

**DOI:** 10.1111/gbb.12872

**Published:** 2023-10-24

**Authors:** Hannah L. Peter, Marina Giglberger, Fabian Streit, Josef Frank, Ludwig Kreuzpointner, Marcella Rietschel, Brigitte M. Kudielka, Stefan Wüst

**Affiliations:** ^1^ Institute of Psychology University of Regensburg Regensburg Germany; ^2^ Department of Genetic Epidemiology in Psychiatry, Central Institute of Mental Health University of Mannheim Mannheim Germany

**Keywords:** ambulatory assessment, chronic stress, cortisol awakening response, depression, neuroticism, polygenic scores, quasi‐experimental design

## Abstract

Genetic factors contribute significantly to interindividual differences in the susceptibility to stress‐related disorders. As stress can also be conceptualized as environmental exposure, controlled gene–environment interaction (GxE) studies with an in‐depth phenotyping may help to unravel mechanisms underlying the interplay between genetic factors and stress. In a prospective‐longitudinal quasi‐experimental study, we investigated whether polygenic scores (PGS) for depression (DEP‐PGS) and neuroticism (NEU‐PGS), respectively, were associated with responses to chronic stress in daily life. We examined law students (*n* = 432) over 13 months. Participants in the stress group experienced a long‐lasting stress phase, namely the preparation for the first state examination for law students. The control group consisted of law students without particular stress exposure. In the present manuscript, we analyzed perceived stress levels assessed at high frequency and in an ecologically valid manner by ambulatory assessments as well as depression symptoms and two parameters of the cortisol awakening response. The latter was only assessed in a subsample (*n* = 196). No associations between the DEP‐PGS and stress‐related variables were found. However, for the NEU‐PGS we found a significant GxE effect. Only in individuals experiencing academic stress a higher PGS for neuroticism predicted stronger increases of perceived stress levels until the exam. At baseline, a higher NEU‐PGS was associated with higher perceived stress levels in both groups. Despite the small sample size, we provide preliminary evidence that the genetic disposition for neuroticism is associated with stress level increases in daily life during a long‐lasting stress period.

## Graphical abstract

In the LawSTRESS project, a study with a prospective‐longitudinal quasi‐experimental design, German law students experiencing (stress group) or not experiencing (control group) a long‐lasting stress phase were examined. We analyzed whether polygenic scores (PGS) for depression and neuroticism, respectively, were associated with perceived stress levels in daily life. For the neuroticism PGS we found a significant GxE effect; only in the stress group a higher PGS predicted stronger increases of perceived stress levels over 13 months.

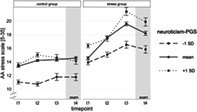

## 1 INTRODUCTION

Differences in the susceptibility to mental disorders can in part be explained by genetic factors.^1^, ^2^ Regarding depression, twin studies have estimated the heritability to range between 30% and 40%^3^, ^4^, ^5^ and recent large‐scale genome‐wide association studies (GWAS) have identified several related genomic loci.^6^, ^7^ Similarly, numerous loci have been found to be associated with neuroticism,^8^, ^9^ a personality trait which is also a risk factor for mental disorders.^10^, ^11^, ^12^ In twin studies the heritability of neuroticism was found to be around 40%.^13^, ^14^ Overall, recent GWAS confirmed the hypothesized polygenic nature of complex traits and common disorders, with each associated genetic variant being characterized by a very small effect size.^7^, ^8^, ^15^, ^16^

Identification of genetic main effects and of gene variants forming the molecular basis of these effects are important goals of genetic psychiatry. Moreover, gene–environment interaction (GxE) effects are of substantial interest as well.^17^, ^18^, ^19^ At this point, an interesting overlap emerges between genetics and stress research. Modern stress concepts define stress as a transactional relationship between individuals and their environment.^20^ Nevertheless, stress—or in the narrow sense ‘stressors’—can also be perceived as a significant environmental exposure, which is known for decades to increase the risk for several physical as well as mental disorders including depression.^21^, ^22^, ^23^, ^24^, ^25^, ^26^ Furthermore, neuroticism is related to stress sensitivity. Individuals with high levels of neuroticism perceive life as more stressful and report a higher negative affect in response to stress.^27^, ^28^, ^29^

The majority of past GxE studies related to stress research investigated single candidate genes.^17^, ^18^, ^30^, ^31^ However, in the last decade a growing number of genome‐wide by environment interaction studies (GWEIS)^32^, ^33^ as well as polygenic score (PGS) analyses studies emerged.^34^, ^35^, ^36^, ^37^ PGS are estimates of the genetic disposition to a specific trait at the individual level. The effects of many common SNPs are aggregated to account for the polygenic nature of stress‐related disorders and complex behavior.^38^, ^39^ PGS are estimated as the sum of effect alleles weighted by the corresponding estimated effect size of this allele derived from a respective GWAS on the examined trait.^38^ This approach enables to estimate the genetic disposition to a specific trait across the whole genome at the individual level. PGS analyses are an interesting approach to combine psychological and genetic research and to examine GxE effects with more predictive power than single candidate SNP analyses.^40^, ^41^, ^42^ Studies applying the PGS approach to investigate the interplay between genetic and environmental factors, mainly examined childhood trauma and stressful life events.^34^, ^35^, ^43^ So far, results have been mixed.^34^, ^35^, ^43^, ^44^, ^45^, ^46^, ^47^, ^48^ These inconsistent results probably arise from methodological differences and lack of power. Most of the studies focused on retrospective assessments of stressful life events or childhood maltreatment, an approach which is important but at the same time known to be susceptible to recall bias and cognitive errors.^49^, ^50^, ^51^ If feasible, prospective‐longitudinal designs are preferable as they have the potential to uncover causal relationships between stress exposure and alterations in psychobiological systems or disease vulnerabilities. In our view, the combination of such a design with methods like ambulatory assessment (AA) which enables the ecologically valid recording of momentary experience and behavior^52^ is a promising approach to overcome some difficulties of previous studies.^53^ Moreover, AA offers a high reliability due to repeated real‐time and real‐life measurements and was proposed to provide higher sensitivity for examining the interplay between psychological and biological processes.^52^, ^54^ First studies investigating associations between PGS and carefully assessed phenotypes obtained promising results.^55^, ^56^, ^57^ Schick et al.^57^ investigated 248 subjects and found that a PGS for schizophrenia (SCZ‐PGS) was associated with psychotic experiences in response to minor daily stressors. Another study, investigating 70 subjects with AA, reported that a SCZ‐PGS and the quantity of social contacts were associated with positive affect in daily life.^55^ These studies document the usefulness of PGS analyses in studies with smaller sample sizes and highlight the importance to investigate the association between genetic factors and precisely assessed (intermediate) phenotypes to understand mechanisms involved in the etiology of psychiatric disorders and stress regulation. Conceptually, a thorough phenotyping may increase the size of the effect of interest. However, it should be noted that phenotyping quality can surely not fully compensate for the lack of power in studies with small samples.

Our prospective‐longitudinal quasi‐experimental LawSTRESS project aimed at identifying predictors of chronic stress responses in daily life to unravel molecular mechanism of stress regulation and interindividual differences.^58^ Besides psychological and neural factors, the identification of genetic predictors was of special interest. The main objective of the genetic study arm was to perform gene‐set analyses to examine the association between chronic stress responses and the overall genetic variability of the neuropeptide S (NPS) system, consisting of the genes for NPS and its receptor (NPSR1).^59^ Our previous analyses did not confirm associations between genetic variability in the NPS/NPSR1 system and perceived stress levels or anxiety symptoms. However, we found a significant association with alterations of salivary cortisol regulation, in particular under the environmental condition ‘chronic stress exposure’. The aim of the present analyses was to expand this candidate gene approach and to conduct secondary exploratory PGS analyses. We investigated the associations between a PGS for depression (DEP‐PGS) and neuroticism (NEU‐PGS), respectively, and three stress‐related phenotypes, namely perceived stress levels, depression symptoms and cortisol regulation, assessed repeatedly over the 13 months observation period. Law students were examined while preparing for their first state examination which is considered one of the most stressful exam periods in the German university system. In Bavaria, this exam consists of six written exams of several hours each within 8 days, it can be repeated only once, has a failure rate of about 24% to 30%, and the final mark is of crucial importance for the future career of the candidate. Additionally, we assessed an adequate control group, consisting of law students in earlier semesters experiencing usual study‐related workload. Especially, perceived stress levels measured at high frequency via AA in 432 participants represent an interesting in‐depth phenotype which complements previous studies using categorial phenotypes.^35^, ^43^ The AA was combined with assessments of the cortisol awakening response (CAR). The CAR is characterized by a sharp increase of cortisol concentrations in the first 30 to 45 minutes after morning awakening.^60^, ^61^ Regulatory mechanisms of the CAR partly differ from the basal diurnal secretion pattern as it is evoked by morning awakening.^62^ A moderate heritability of the CAR was consistently found in twin studies.^63^, ^64^ Besides the repeated measurement of the stress related variables and the detailed phenotyping, the (quasi‐) experimental design of our study holds further advantages for the investigation of GxE effects. To a certain degree, it reduces the measurement error in the environmental component and diminishes the uncontrollable influence of gene–environment correlations, which hinders the discovery of true GxE interactions.^65^, ^66^

The investigation of the genetic disposition to depression and neuroticism seems promising for several reasons. Besides the high relevance of depression and neuroticism in stress research, large‐scale GWAS for both phenotypes are available, enabling to compute PGS with substantial power.^7^, ^8^ Furthermore, although both phenotypes are highly correlated, genetically as well as phenotypically, they also have distinct genetic influences.^5^, ^9^, ^67^ Thus, we assume that they complement each other in the search for genetic predictors of stress responses during a long‐lasting stress phase as the PGS for depression captures the genetic disposition to develop a clinical depression whereas the NEU‐PGS probably has a broader range and stronger overlap with stress reactivity.

We were especially interested in the GxE effect of the DEP‐PGS as well as the NEU‐PGS in combination with the environmental variable “chronic examination stress.” The main hypothesis was that the DEP‐PGS and the NEU‐PGS predict perceived stress levels which were assessed at high frequency via AA over the observation period. We expected this association particularly in the stress group, experiencing chronic academic stress. Furthermore, we investigated whether alterations in depression symptoms and different parameters of the cortisol awakening response were associated with genetic disposition for depression and neuroticism, respectively.

## 2 MATERIALS AND METHODS

### 2.1 Sample

In the LawSTRESS project, we recruited 470 law students from Bavarian universities. Genetic data were analyzed for 451 participants who completed at least the first sampling timepoint. Another 19 participants were excluded during the quality control (QC) steps of the genetic data (see section 2.4) resulting in a final sample of 432 students for the following analyses. For a detailed sample description of the total sample, health and university study‐related information, and trajectories of several psychological questionnaires, please see https://doi.org/10.5283/epub.51920 and Giglberger et al.^58^

Two different cohorts were recruited, each consisting of a stress group (SG), experiencing a long‐lasting and significant stress phase, namely the preparation for the first state examination for law students, and a control group (CG) experiencing usual study‐related workload. Cohort A consisted of 196 students (SG: *n* = 95 and CG: *n* = 101) mainly from the University of Regensburg. Cohort B comprised 236 (SG: *n* = 123 and CG: *n* = 113) law students from other Bavarian universities who underwent a modified examination protocol (less extensive AA, no CAR data; see Section 2.3.1).

Exclusion criteria were: (self‐reported) current psychiatric, neurological, or endocrine disorders, treatment with psychotropic medications, any other medication affecting central nervous system or endocrine functions, or regular night‐shift work. The study was approved by the local ethics committee. All participants provided written informed consent and received monetary compensation and individual feedback.

### 2.2 General procedure

**FIGURE 1:**
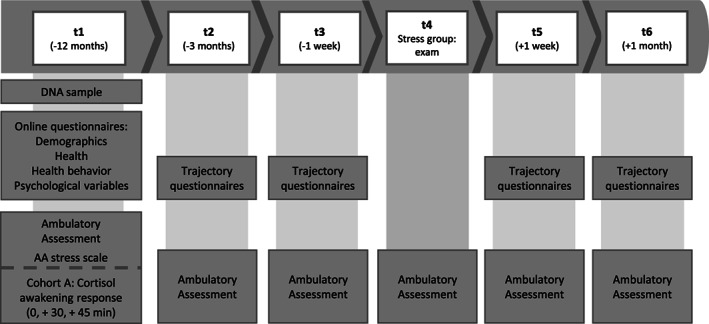
Timing of data collection. For an overview of the whole study procedure of the LawSTRESS project see https://doi.org/10.5283/epub.51920; Reproduced with permission from Peter et al.^59^

As reported elsewhere,^58^ the study comprised six sampling timepoints (t1–t6) over 13 months (Figure 1). T1 for the SG took place 1 year before the exam; the remaining timepoints were scheduled 3 months (t2) and 1 week (t3) prior to the exam, on the weekend during the eight‐days exam period (t4), as well as 1 week (t5) and 1 month (t6) after the exam. For the CG the same procedure applied, except that there was no exam at t4. Data collection lasted from March 2018 until April 2021. At t1, exclusion criteria were checked and written informed consent obtained. An online questionnaire battery to inquire baseline data, psychometrics, physical health, health behavior, and university studies‐related variables was sent out. Furthermore, participants received the material and detailed description for the first AA and a buccal swab for DNA analysis was collected. At t2–t6 the AA was conducted. Moreover, a trajectory questionnaire was assessed at all timepoints except for t4 (Figure 1), comprising health, health behavior and psychological variables.

### 2.3 Acquisition of behavioral and endocrine data

#### 2.3.1 Ambulatory assessment

As previously reported,^58^, ^59^ the AA in cohort A encompassed the collection of saliva samples after awakening for later assessment of the CAR and an assessment of current perceived stress via the newly developed, five‐item AA stress scale. A description of the generation of the AA stress scale consisting of the items ‘time pressure’, ‘relaxed’, ‘tense’, ‘overstrained’ and ‘I am disappointed with my performance’ with a seven‐point Likert scale as response format (‘strongly disagree’ to ‘strongly agree’) can be found in Giglberger et al.^58^ For the AA, the combined smartphone app and web platform movisensXS (Version 1.3.2 to 1.5.13; movisens, Karlsruhe, Germany) was used. At t1, t2, t5, and t6, 10 queries per day were presented on two consecutive working days. At the timepoints close to the exam (t3 and t4), AA was performed on 1 day only to limit the study‐related burden. T4 in the SG (not in the CG) was scheduled at the weekend in the middle of the eight‐days exam period. The first daily query was presented immediately at the individually chosen awakening time between 05:00 and 07:30 a.m. and the last one at 09:00 p.m. The remaining eight queries took place at pseudo‐randomized times between 08:30 a.m. and 08:00 p.m. with a minimum interval of 60 minutes between two queries. Participants who did not have a compatible smartphone received a device provided by the institute (Motorola G4, Motorola Play G4 and Motorola Play G6).

The measurement of the CAR was based on three saliva samples, collected using cortisol Salivettes (Sarstedt, Nümbrecht, Germany) immediately after awakening as well as 30 and 45 min later. Saliva samples were collected on the first day of each AA phase; except for t1, when CAR was assessed on both sampling days. During this period, participants were briefed not to eat, drink (except from water), smoke or brush their teeth. To enhance compliance and sampling accuracy, in 51%–72% (varying over sampling points) of the measurements, functional and non‐functional (‘sham’) electronic monitoring devices to verify times of sample collection (MEMS caps, AARDEX Ltd., Zug, Switzerland) were used.^68^, ^69^ In addition, participants were instructed to transfer a random three‐digit code to the sampling tube for each saliva sampling, which was displayed to them via smartphone. In our lab saliva samples were stored at −20°C until analysis. Samples were assayed in duplicate using a time‐resolved fluorescence immunoassay with fluorometric end‐point detection (DELFIA) at the biochemical laboratory of the University of Trier.^70^ The intra‐assay coefficient of variation was between 4% and 7%; inter‐assay coefficients of variation were between 7% and 9%.

In cohort B, the AA stress scale was assessed via SoSci Survey (alerts via SMS and e‐mail; https://www.soscisurvey.de/)^71^ in the morning at 07:30 a.m. and in the evening at 09:00 p.m. The query had to be answered within 90 min.

#### 2.3.2 Questionnaires

Demographic variables (age, sex, etc.) and different psychological constructs were assessed online with SoSci Survey at t1. Depression symptoms were inquired with the depression subscale of the Hospital Anxiety and Depression Scale (HADS)^72^ at t1, t2, t3, t5 and t6. Please see Giglberger et al.^58^ and https://doi.org/10.5283/epub.51920 for additionally assessed variables not included in the present report.

### 2.4 DNA sampling, genotyping, quality control and genotype imputation

As described previously,^59^ we used a non‐invasive DNA sampling via buccal swabs (Analytik Jena GmbH, Jena) and salting out procedure for DNA isolation.^73^ Genotyping was conducted using the Illumina Infinium™ Global Screening Array 3.0 with Multi‐disease drop in (Illumina, San Diego, CA, USA) at the Life & Brain facilities, Bonn, Germany.

Quality control of the data was conducted with PLINK 1.9 (see www.cog-genomics.org/plink/1.9/).^74^ SNPs with minor allele frequency (MAF) of <0.01, deviating from Hardy–Weinberg equilibrium (HWE) with a *p*‐value of <10^−6^, and with missing data >0.02 were removed. Participants were excluded in case of missingness >0.02, sex‐mismatch, and heterozygosity rate >|0.20|. Filtering for relatedness and population structure was performed on a SNP set filtered for high quality (HWE *p* > 0.02, MAF >0.20, missing rate = 0) and linkage disequilibrium pruning (pairwise *r* ^2^ = 0.10). In the case of relatedness (pi‐hat >0.20), one participant was excluded at random. To adjust for population stratification, principal components (PC) were computed. Outliers on any of the first 20 PCs (|z| > 4.5) were eliminated. In total, 19 participants were excluded. In a last step, data was checked for duplicate SNPs and one was retained at random. Thus, the final data set contained 432 subjects and 476,701 SNPs.

After quality control, genotype imputation was performed with Eagle v2.4.1^75^ and Minimac4.^76^ Data from 1000 Genomes Phase3 v5^77^ was used as reference panel. For the analyses, we used the estimated most likely genotype and only SNPs with an info score ≥0.90. In a last step, data was again checked for MAF of >0.01, for duplicate SNPs, retaining one at random, and SNP rs IDs were added resulting in a total of 5,278,541 SNPs used for PGS analysis. Detailed information on genotyping, quality control steps, and genotype imputation has been previously described in Peter et al.^59^

### 2.5 Polygenic scores

DEP‐PGS for each participant were calculated based on summary statistics of GWAS using data from the Psychiatric Genomics Consortium (PGC), the UK Biobank and 23andMe Inc. (containing 246,363 cases and 561,190 controls).^7^ The NEU‐PGS were computed based on summary statistics of the meta‐analysis of GWAS for neuroticism excluding data from 23andMe, only including data from the UK Biobank, and the Genetics of Personality Consortium (GPC; containing 390,278 subjects).^8^ Calculation of PGS was performed with PRSice 2.3.3.^38^ PGS were calculated as weighted sums of each participant's trait‐associated alleles across SNPs retained after clumping (250 kb sliding window, linkage disequilibrium *r* ^2^ > 0.1) and after removal of variants within the major histocompatibility complex region (−‐x‐range chr6 26,000,000–33,000,000). For the inclusion of SNPs, a *p*‐value threshold (*P*_T_) of ≤0.05 for DEP‐PGS and *P*_T_ ≤ 0.10 for NEU‐PGS, respectively, was applied since they explained the largest proportion of phenotypic variance in their original GWAS.^7^, ^8^ Otherwise, default settings were used. The final DEP‐PGS contained 29,523 SNPs and the NEU‐PGS contained 49,816 SNPs. Moreover, we tested an additional PGS method using continuous shrinkage prior on SNP effect size (so called PGScs)^78^ on our significant results to test their consistency across different approaches.

As positive control, PGS for height were calculated with PRSice using summary statistics from Yengo et al.^79^ for the *p*‐value thresholds 5 × 10^−08^, 10^−06^, 0.0001, 0.001, 0.01, 0.05, 0.10, 0.2, 0.5, and 1. Height PGS were tested for association with measured height and with sex, age and PC1 to 5 as covariates.

### 2.6 Statistical analysis

Because of the hierarchical and longitudinal structure of our data, the associations between the PGS and the stress‐related variables were tested in two level linear mixed models (timepoints nested within participants) using R (version 4.0.3).^80^ Since we were interested in the association between the PGS and the trajectory of the investigated variables under chronic stress conditions, only the timepoints until the exam were included (t1–t4, for depression: t1‐t3). In a first step, the final group models investigating group differences over the observation period are shortly presented. Some of these models have already been presented in Giglberger et al.^58^ and Peter et al.^59^ However, sample sizes are slightly different, only timepoints until the exam were examined, and aggregated parameters of the CAR were used instead of single cortisol values (see Section 2.6.1). The aggregation of the single cortisol values was necessary in order to facilitate interpretability of the final models. In a second step, we then added the PGS to these models to test our hypotheses. All models were estimated with Maximum Likelihood and the significance level was set at *α* = 0.05.

#### 2.6.1 Model structure of the group models to test for group differences

The trajectories of the AA stress scale (*n* = 432; observations = 12,230) and depression symptoms (*n* = 432; observations = 1231) were calculated using generalized linear mixed models (package glmmTMB).^81^ The final group models (group. model) contained the fixed effects *group* (0 = CG, 1 = SG), *timepoint* (centered at the first timepoint) as linear and quadratic time trend, their interactions with *group* (0 = CG; 1 = SG), and the covariates *sex* (0 = men; 1 = women) and *cohort* (0 = cohort A; 1 = cohort B), the latter only in the AA stress scale model. To account for dependencies in the data, random intercepts and slopes for *timepoint* by participant were estimated. To model the CAR (*n* = 196; observations = 919), we used the two parameters area under the curve with respect to the ground (*AUCg*), serving as measurement of the total hormonal output and the AUC with respect to the increase (*AUCi*), representing the time‐dependent change of cortisol in the morning.^60^ Raw cortisol values were used since the residuals of the final models displayed satisfactory approximation to normal distribution. Fourteen cortisol values were excluded because of participants' self‐reported nonadherence to the study protocol and physiologically implausible values (e.g., only one extremely high value within one CAR assessment). Linear mixed models were computed with the package nlme.^82^ The models contained similar fixed effects as presented above, except that *AUCg* was best represented by a linear time trend only and without a random slope for *timepoint*. As covariates, we added the person‐mean centered variable *time of awakening* (in minutes) and instead of *sex* the *hormonal status* was used (0 = women not using hormonal contraceptives, 1 = women using hormonal contraceptives and 2 = men).

#### 2.6.2 Models containing PGS to test main hypotheses

To test our hypotheses that the PGS for depression and the PGS for neuroticism are associated with the trajectories of the stress‐related variables, the following fixed effects were added simultaneously to the group. model: *PGS*, the interaction of *PGS* with *group* and the linear and quadratic time trend as well as the three‐way interaction (*timepoint/timepoint* ^2^ × *group* × *PGS*; PGS.model). The PGS were z‐standardized; for models containing only the SG or the CG, the PGS were standardized within the group. Adding the *PGS*, we used the decrease in AIC and log‐likelihood ratio test to evaluate improvement in model fit. In order to control for genetic ancestry, grand mean‐centered PC1‐5 were added to the PGS.model (PC.model). The covariates *PC1‐5* were only retained in the model if a significant improvement in model fit was observed (AIC and change in −2log‐likelihood with χ^2^‐test) or if their addition led to changes in the results. To account for possible confounders, an alternative approach was evaluated to control for covariates and possible interactions with the PGS and the environmental variable.^83^ Therefore, we exploratively tested whether the addition of all the interaction terms (*PGS* × covariates and *group* × covariates) changed our results. In total, eight main models were tested, four for the DEP‐PGS and four for the NEU‐PGS, respectively. *Post‐hoc*, additional models were computed for the variables AA stress scale and *AUCg*. In order to unravel the interaction between the NEU‐PGS and the group, separate models for the SG and the CG were calculated. The explained variance of the fixed effects of the final models was calculated via marginal R squared (*R* ^2^).^84^ The predictive power of the PGS was then measured by the ‘incremental *R* ^2^’, defined as the increase of marginal *R* ^2^ when the PGS and its interactions were added to the model.

## 3 RESULTS

### 3.1 Demographics and descriptives

Demographic information of the sample can be taken from Table 1. As the control group consisted of students in earlier semesters, the significant age difference between SG and CG was not surprising (*t*(430) = −11.45, *p* < 0.001). Descriptives of the dependent variables and the PGS as well as information about the distribution of the PGS for depression and neuroticism can be found in the supplements (Supplementary Tables **S1**‐S6 and Supplementary Figures **S1**‐S6). No significant differences were found regarding the DEP‐PGS or the NEU‐PGS between the groups or cohorts (*t*(430) > 1.57, *ps* >0.117). The correlation between the DEP‐PGS and NEU‐PGS was *r*(430) = 0.30, *p* < 0.001.

**TABLE 1:** Demographic characteristics of the total sample.

	Stress group	Control group
*n*	218	214
Age (Mean ± standard deviation)	22.96 (± 1.72)	21.04 (± 1.77)
Women	*n* = 160 (73%)	*n* = 165 (77%)
Women using hormonal contraception	*n* = 102	*n* = 103

*Note*: Recruiting was separated in two cohorts. Cohort A (*n* = 196) underwent the elaborate study protocol with laboratory visits in Regensburg and the assessment of the cortisol awakening response whereas cohort B (*n* = 236) consisted of law students from other Bavarian universities who completed a less detailed study protocol (see Section 2.1).

### 3.2 Association of DEP‐PGS and NEU‐PGS with stress‐related phenotypes

The focus of our analysis was to investigate the association of the DEP‐PGS and the NEU‐PGS with the rise in momentary perceived stress levels due to the examination stress. Furthermore, we assessed whether the PGS were associated with the alterations in depression symptoms as well as the CAR parameters *AUCg* and *AUCi* until the exam at t4. As recently reported, trajectories of perceived stress levels, depression symptoms, and the CAR were significantly different between SG and CG.^58^, ^59^ The present analyses, based in part on different sample sizes and aggregated variables for the CAR (see Section 2.6.1), yielded very similar results.

In none of the final PGS models the addition of the covariates *PC1‐5* did improve the model fit. Moreover, only minor effects on the beta values but no alterations of the overall results were observed. Furthermore, we found no significant changes in results regarding our predictors of interest after adding all covariates in interaction with *PGS* and *group*. Therefore, always the less complex PGS.model without covariates and additional interaction terms is presented in the following. Information on PC.models that include *PC1‐5* as well as the interaction terms can be found in Supplementary Tables S7–S24. The positive control, height PGS, showed a positive association with measured height (strongest association: *P*_T_ ≤0.10, *R* ^2^ = 12.26%).

#### 3.2.1 AA stress scale

The AA stress scale represented the most relevant self‐report instrument used in the present study as it was assessed at high frequency as well as in real‐time and real‐life to capture the momentary perceived stress. Considering only t1–t4, a compliance rate of 94% was reached. As already presented elsewhere,^59^ we found significant differences between SG and CG in the trajectories of perceived stress levels until the exam at t4 (*timepoint* × *SG b* = 0.18, *p* < 0.001; *timepoint* ^2^ × *SG b* = −0.04, *p* < 0.001). Mean perceived stress levels in the SG increased, whereas perceived stress levels in the CG stayed relatively stable (see Supplementary Figure S7 and Table S7). Additionally, the SG showed slightly higher perceived stress levels at the baseline measurement, compared with the CG, resulting in a significant difference at t1 (*SG b* = 0.10, *p* = 0.003).

**FIGURE 2:**
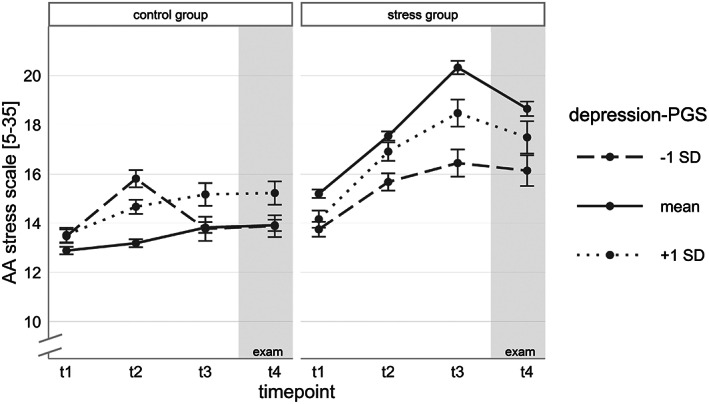
Time course of mean perceived stress levels (±SEM) in the stress group (SG) and control group (CG) separated by polygenic score (PGS) for depression (grouping based on standard deviation (SD) for illustrative purposes only).

**FIGURE 3:**
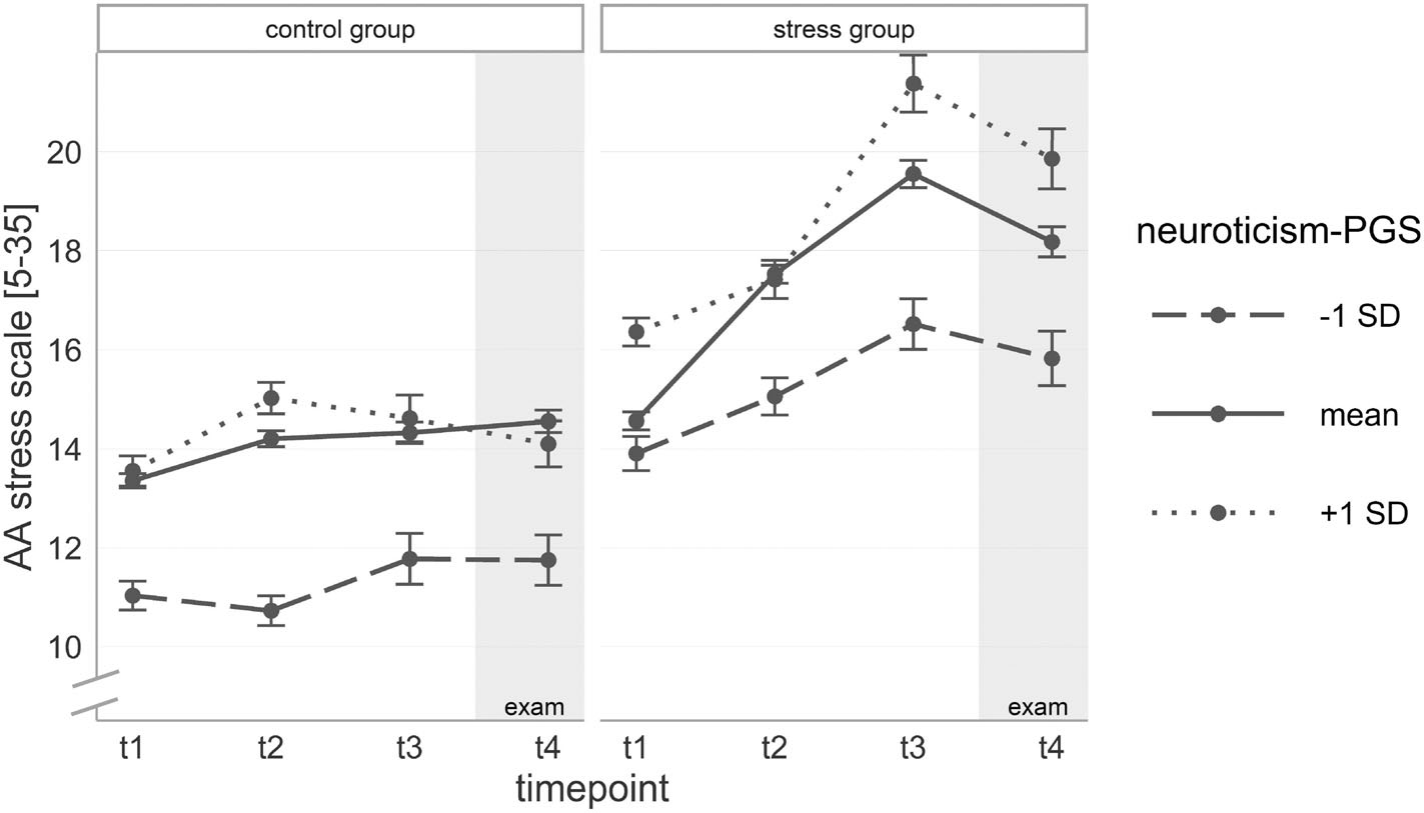
Time course of mean perceived stress levels (±SEM) in the stress group (SG) and control group (CG) separated by polygenic score (PGS) for neuroticism (grouping based on standard deviation (SD) for illustrative purposes only).

Entering the DEP‐PGS to the model did not lead to an improvement of the model (group.model vs. PGS.model *χ* ^2^(6) = 8.63, *p* = 0.196, ΔAIC = −3.37). Therefore, our hypothesis that the increase of stress perception in the SG is predicted by the DEP‐PGS could not be confirmed (Figure 2). Please see Supplementary Table S7 for all model parameters, the explained variance and a detailed model comparison. However, adding the NEU‐PGS resulted in an improved model fit (group.model vs. PGS.model *χ* ^2^(6) = 38.76, *p* < 0.001, ΔAIC = 26.77), indicating a significant association between NEU‐PGS and perceived stress levels. We found a significant effect of the NEU‐PGS on the trajectory of perceived stress levels in the SG (*NEU‐PGS* × *timepoint* × *SG b* = −0.06, *p* = 0.001; *NEU‐PGS* × *timepoint* ^2^ × *SG b* = 0.02, *p* < 0.001) but not in the CG (*NEU‐PGS* × *timepoint b* = 0.02, *p* = 0.093). Only the quadratic trend in the CG seems to be associated slightly with the PGS *(NEU‐PGS* × *timepoint* ^2^ *b* = −0.01, *p* = 0.005). However, as there is nearly no change in perceived stress levels of the CG over the time period, results should be viewed with caution. Additionally, we found a significant effect of the NEU‐PGS on the baseline measure of perceived stress levels at t1 (*NEU‐PGS b* = 0.05, *p* = 0.034). The effect did not differ between the two groups (*NEU‐PGS* × *SG b* = 0.02, *p* = 0.460). To unravel the interaction, separate models were calculated for both groups and this analysis confirmed the result of the total model (see Supplementary Tables S9 and S10). Thus, individuals with a low genetic disposition for neuroticism showed lower perceived stress levels at t1 in both groups as well as a lower increase of stress levels in the SG under chronic examination stress (see Figure 3, Table 2 and Supplementary Table S8). In the PGS.model, 1.53% of the variance could be explained by the NEU‐PGS parameters. Additional analyses with the NEU‐PGScs confirmed these results with only minor differences (see Supplementary Tables S11–S13 for all model parameters).

**TABLE 2:** Parameter estimates for overall effects of the final PGS.model with perceived stress as dependent variable and the polygenic score for neuroticism as predictor.

Fixed effects	Estimates	*SE*	*p*
Intercept	2.46	0.04	<0.001
Timepoint	0.07	0.01	<0.001
Timepoint^2^	−0.02	0.00	<0.001
Women (vs. men)	0.07	0.03	0.049
Cohort B (vs. cohort A)	0.18	0.03	<0.001
SG (vs. CG)	0.09	0.03	0.004
Timepoint × SG	0.18	0.02	<0.001
Timepoint^2^ × SG	−0.05	0.00	<0.001
PGS	0.05	0.02	0.034
PGS × SG	0.02	0.03	0.460
PGS × timepoint	0.02	0.01	0.093
PGS × timepoint^2^	−0.01	0.00	0.005
PGS × timepoint × SG	−0.06	0.02	0.001
PGS × timepoint^2^ × SG	0.02	0.00	< 0.001

Random effects	SD	Correlation Intercept	
Participant (Intercept)	0.31		
Timepoint	0.11	−0.27	

Abbreviations: CG, Control group; PGS, polygenic score; SD, standard deviation; SE, standard error; SG, stress group.

#### 3.2.2 Depression symptoms

Regarding depression symptoms, we found a difference between the SG and the CG over time (*timepoint* × *SG b* = 0.50, *p* < 0.001; *timepoint* ^2^ × *SG b* = −0.11, *p* = 0.044). No difference was found at the baseline measure (t1) between both groups (*SG b* = −0.08, *p* = 0.285). The SG showed a steep increase until the exam, whereas the CG stayed relatively stable (see Supplementary Figure S8 and Table S14). As already presented in Giglberger et al.^58^ 18% of the students in the SG exceeded the clinically relevant score of 11 for depression symptoms at t3, compared with 2% at the baseline measurement and 3%–5% of the CG. Neither including the DEP‐PGS nor the NEU‐PGS resulted in an improved model fit (DEP‐PGS: group.model vs. PGS.model *χ* ^2^(6) = 7.07, *p* = 0.314, ΔAIC = −4.92; NEU‐PGS: group.model vs. PGS.model *χ* ^2^(6) = 8.89, *p* = 0.180, ΔAIC = −3.11). Thus, no association between the DEP‐PGS nor the NEU‐PGS and depression symptoms could be assumed (see Supplementary Figures S9 and S10 & Tables S14 and S15).

#### 3.2.3 Cortisol awakening response: AUCg and AUCi

Regarding the CAR parameters *AUCg* and *AUCi*, we found significant differences between both groups over time (*AUCg*: *timepoint* × *SG b* = −19.15, *p* = 0.016; *AUCi*: *timepoint* ^2^ × *SG b* = −19.81, *p* = 0.015). No differences were found at t1 (*AUCg*: *SG b* = 2.83, *p* = 0.888; *AUCi*: *SG b* = −10.60, *p* = 0.511). The SG showed a strong decline of *AUCg* and *AUCi* at t4 (see Supplementary Figures S11 and S14 & Tables S16 and S23). Thus, our previously reported finding of a blunted CAR in the SG compared with the CG could be reproduced by the present analyses of the aggregated CAR parameters.^58^

For the *AUCg* and the *AUCi*, addition of the DEP‐PGS did not improve the global model fit (*AUCg*: group.model vs. PGS.model *χ* ^2^(6) = 2.64, *p* = 0.620, ΔAIC = −5.36; *AUCi*: group.model vs. PGS.model *χ* ^2^(6) = 2.42, *p* = 0.877, ΔAIC = −9.58). Hence, the DEP‐PGS was not related to the CAR (see Supplementary Figures S12 and S15 & Tables S16 and S23). Also, regarding the NEU‐PGS and the CAR we could not confirm our GxE hypothesis. The NEU‐PGS was not related to the alteration of the *AUCg* and *AUCi* over the time period (*AUCg*: *ps* ≥0.538; AUCi: *ps* ≥ 0.068; see Supplementary Tables S17 and S24). We found an association between the NEU‐PGS and the baseline measurement of the *AUCg* which differed significantly between the two groups (*PGS b* = −47.40, *p* < 0.001; *PGS* × SG *b* = 48.23, *p* = 0.014). Subsequently calculated separate models for the CG and SG confirmed this association solely for the CG (CG.model vs. PGS.model *χ* ^2^(2) = 12.33, *p* = 0.002, ΔAIC = 8.33; *PGS b* = −48.42, *p* < 0.001; Supplementary Table S18) but not for the SG (SG.model vs. PGS.model *χ* ^2^(2) = 0.21, *p* = 0.900, ΔAIC = −3.79; *PGS b* = 1.49, *p* = 0.911; Supplementary Table S19). The PGS parameters in the model containing only the CG explained 5.77% of the variance. For the *AUCi*, a tendency for this association within the CG could also be found (*PGS b* = −25.33, *p* = 0.021), although the model did not improve significantly (group.model vs. PGS.model *χ* ^2^(6) = 7.75, *p* = 0.257, ΔAIC = 0.01; Supplementary Table S24). Individuals in the CG with lower genetic disposition for neuroticism showed a higher *AUCg* (Supplementary Figure S13) and, probably, also a stronger increase in cortisol upon awakening at t1 (Supplementary Figure S19). Results for the AUCg were confirmed by the analyses with the NEU‐PGScs (see Supplementary Tables S20–S22).

## 4 DISCUSSION

In the present analyses, we studied if polygenic scores capturing the genetic disposition for depression and neuroticism, were associated with chronic stress responses in everyday life. The focus was to investigate GxE effects of both PGS and the environmental exposure ‘chronic examination stress’ in a quasi‐experimental and prospective longitudinal design. The main outcome variable, the increase in perceived stress levels, was assessed at high frequency and in an ecological valid manner via AA in 432 subjects. As previously reported,^58^ significant differences between the SG and the CG over the 13 months period could be found: The SG showed increases in perceived stress levels and depression symptoms as well as decreases in the CAR parameters *AUCg* and *AUCi* until the exam compared with the CG. Hence, we can conclude that the chronic examination stress resulted in alterations in psychological well‐being as well as in cortisol regulation.

To examine whether these alterations are associated with genetic factors, PGS for depression and neuroticism were investigated. Both phenotypes are related to stress.^27^, ^85^, ^86^, ^87^ Although they are highly correlated, both phenotypically as well as genetically,^5^, ^8^, ^9^, ^88^ they have also distinct genetic influences^67^ complementing each other in the search for genetic factors influencing chronic stress responses. Contrary to our hypothesis, no relation was found between the DEP‐PGS and perceived stress levels over the observation period. Thus, we did not observe any difference between individuals with elevated or low polygenic disposition for depression regarding their perceived stress levels in daily life over a long‐lasting stress phase. Usually, phenotyping in large‐scale GWAS is not very extensive and recent studies showed that this strategy can result in unspecific PGS capturing not only the risk to develop clinically‐relevant depression but also related constructs and comorbid disorders.^89^, ^90^ However, it can be assumed that the DEP‐PGS particularly represents the risk for clinical depression as the investigated GWAS sample was enriched for patients with diagnosed depression. This risk is probably not fully congruent with the risk to reach high perceived stress levels in the context of academic stress. Thus, we suppose that the DEP‐PGS was not entirely suitable to uncover GxE effects in our study sample consisting of healthy students. Although academic stress was shown to be associated with increased depression symptoms,^91^, ^92^ we expected that the majority of our participants would be rather stress resilient. This notion was supported by our findings that most of the students showed a fast recovery after the exam regarding perceived stress levels as well as other psychometric variables, including anxiety and depression symptoms, reported sleep disturbances, and other facets of chronic stress.^58^ It appears plausible that a larger and more heterogenic sample regarding stress vulnerability would be needed to find meaningful associations between DEP‐PGS and perceived stress levels. Regarding the NEU‐PGS, we found support for our hypothesis as we observed a significant GxE effect. The higher the genetic disposition for neuroticism, the more pronounced was the increase of perceived stress until the exam at t4 in the SG. Additionally, we observed an effect at the baseline measurement in both groups. The higher the NEU‐PGS, the higher were perceived stress levels. The PGS and the PGS × stress exposure effect explained 1.53% of the variance in perceived stress levels. The present findings are in accordance with previous studies reporting an association between neuroticism and stress sensitivity.^28^, ^29^, ^93^ Furthermore, it was shown in a twin study using AA that the phenotypic association between the variability in negative affect in daily life and neuroticism can be partly explained by genetic effects.^94^

Taken together, our analyses using PGS on the individual level, reflecting the polygenicity of neuroticism, expand the current knowledge as they suggest a shared genetic basis of neuroticism and reported momentary stress levels under normal conditions as well as under chronic stress conditions. Furthermore, the findings support the notion that the NEU‐PGS which was proposed to capture the genetic predisposition to subclinical symptoms of anxiety and depression^95^ has a stronger overlap with stress reactivity than the DEP‐PGS. We assume that the NEU‐PGS probably reflects partly the genetic disposition of how individuals react to stress whereas the DEP‐PGS reflects to a higher extent the genetic susceptibility for the disorder itself.

Regarding the prediction of depression symptoms, no effect of neither the DEP‐PGS nor the NEU‐PGS was found. Thus, in our sample the genetic disposition to depression and neuroticism was not related to depression symptoms. Several reasons why we failed to find any association with depression symptoms are conceivable. First, the lack of power due to the small sample size has to be noted, especially since depression symptoms were not assessed via AA in contrast to perceived stress levels. This probably resulted in a lower validity due to the lower proximity to the momentary experience as well as in a lower reliability of the self‐report as symptoms were only assessed once per measurement timepoint. Second, although we found an increase in depression symptoms in the SG, most participants reported only low to moderate depression symptoms, in particular compared with clinical cases. Thus, our variance in the outcome variable could have been too small. Other recent studies which have used DEP‐PGS or NEU‐PGS did find significant GxE effects on depression symptoms.^34^, ^37^, ^96^, ^97^ Fang et al.^34^ examined 5227 training physicians under chronic stress conditions, more precisely during their medical internship year. They found that depression symptoms under stress were predicted by DEP‐PGS, and that this association was stronger than with depression symptoms at baseline. In another longitudinal study, Li et al.^37^ found a significant association between a NEU‐PGS and late life depression investigating a sample of 4877 participants. This association was partly mediated by retrospectively assessed stressful life events. While the phenotyping in these investigations was less extensive, the samples were considerably larger than in our study, which probably explains why Fang et al.^34^ as well as Li et al.^37^ could detect an effect of the PGS on depression symptoms. Two additional interesting longitudinal studies in this context with slightly smaller sample sizes also found significant associations between DEP‐PGS and depression symptoms.^46^, ^98^ However, results are not necessarily comparable to our study as the investigated trauma‐like type of stressor (motor vehicle collision and death of spouse) differed substantially from chronic academic stress.

Investigating the two CAR parameters, *AUCg* and *AUCi*, we found no association with the DEP‐PGS. Furthermore, no association between the NEU‐PGS and alterations of the CAR parameters under chronic stress conditions was observed. In general, it is a well‐known phenomenon that biological indicators of stress are often not or only moderately correlated with subjective stress‐related variables.^58^, ^99^, ^100^ As the PGS were primarily generated based on self‐report data, they predominantly capture the genetic disposition for phenotypes assessed on a subjective psychological level. Thus, the genes influencing these phenotypes may show only limited overlap with the genes modulating alterations of the CAR under chronic examination stress. The baseline effect of the NEU‐PGS on the *AUCg* only in the CG was somewhat unexpected, as we would assume that any effect at the baseline should be visible in both groups. Therefore, and due to the fact that the power of the CAR analyses was substantially lower compared with analyses of perceived stress levels and depression symptoms (*n* = 196, less frequent assessment), results should be interpreted with caution.

Our study has some limitations that need to be considered. Our sample consisted of young students who probably have better overall health and a higher socioeconomic status compared with the general population. Furthermore, only students with European ancestry were investigated. Thus, the generalizability of the present results is limited. Furthermore, like any other study examining real‐life stress, a certain selection bias cannot be ruled out. Students who already felt stressed by their regular study program and who anticipated an exceedingly stressful exam (preparation phase) did possibly not volunteer to participate in a study that was related to (modest) additional burden. Therefore, it might be possible that we underestimated the mean stress load in the stress group to a certain extent. An obvious limitation of our quasi‐experimental design was the missing randomization regarding the assignment to the stress and the control group. To compensate for this issue, our CG contained individuals who were as similar as possible to our SG participants. This rather conservative strategy might have caused an underestimation of group differences as students in the CG as well had substantial study‐related stress. Additionally, as mentioned earlier, the sample size has to be discussed. Although some features of our study design, as for example the quasi‐experimental design as well as the repeated measurements, likely increased the power, our sample size was quite small, particularly for the analysis of depression symptoms and the CAR. Hence, the statistical power to show three‐way interaction effects was limited. In addition, it should be emphasized that, consistent with the exploratory nature of our PGS analyses, no correction for multiple testing was applied. It should also be acknowledged that a small sample size can increase the likelihood of false positive findings. Therefore, the presented findings need to be replicated in an independent sample. Additionally, the number of covariates within the model could have caused overfitting with too small variance left for the PGS to explain, possibly further reducing the power of our analyses. However, we assume that overfitting due to the number of covariates did not impact results as no alterations of the reported results were observed recomputing the models without covariates. It further has to be noted that the variance currently explained by PGS represents only a marginal proportion of genetic contribution and therefore is still very small.^101^

In summary, we conclude that the present PGS analysis in a cohort that has been thoroughly phenotyped in a longitudinal study including a meaningful, long‐lasting and real‐life stress exposure, provided relevant information on the association between genetic disposition and chronic stress responses in daily life. In particular, we found that individuals with a higher NEU‐PGS were more stress sensitive, as they generally reported higher perceived stress levels and showed stronger increases over the stressful period. Assumed associations between genetic disposition for depression and stress‐related phenotypes could not be confirmed. Due the small sample further replication is needed. Future studies could combine polygenic scores with additional factors, such as brain activation changes in response to acute stress, functional connectivity, or other physiological stress markers to predict chronic stress responses in daily life. Such a combination of PGS and other relevant factors was already shown to be useful for disease risk stratification and for the prediction of medication treatment outcomes.^102^, ^103^

## FUNDING INFORMATION

This work was funded by the ‘German Research Foundation’ (DFG) project WU392/9–1 (to S.W.), the German Federal Ministry of Education and Research (BMBF) through ERA‐NET NEURON, ‘SynSchiz—Linking synaptic dysfunction to disease mechanisms in schizophrenia—a multilevel investigation’ (01EW1810 to M.R.), and through ERA‐NET NEURON ‘Impact of early life metabolic and psychosocial stress on susceptibility to mental disorders; from converging epigenetic signatures to novel targets for therapeutic intervention’ (01EW1904 to M.R.). In addition, the study was supported by the DGPs section ‘Biological Psychology and Neuropsychology’ (research fellowship during corona pandemic; to H.L.P and M.G.), the ‘Financial Incentive System to Promote Gender Equality’ (FAS) of the University of Regensburg (to H.L.P and M.G.) and the ‘Bavarian Programme to Realise Equal Opportunities for Women in Research and Teaching 2021’ (to H.L.P.).

## CONFLICT OF INTEREST STATEMENT

The authors declare no conflicts of interest.

## Supporting information


**Data S1:** Supporting Information.

## Data Availability

Research data are not shared. GWAS summary statistics for the DEP‐PGS, include data from 23andme and can be requested from 23andMe.^7^ GWAS summary statistics used for NEU‐PGS are publicly available (https://ctg.cncr.nl/software/summary_statistics).^8^

## References

[1] Pluess M . Individual differences in environmental sensitivity. Child Dev Perspect. 2015;9(3):138‐143. doi:10.1111/cdep.12120

[2] Caspi A , Moffitt TE . Gene–environment interactions in psychiatry: joining forces with neuroscience. Nat Rev Neurosci. 2006;7(7):583‐590. doi:10.1038/nrn1925 16791147

[3] Sullivan PF , Neale MC , Kendler KS . Genetic epidemiology of major depression: review and meta‐analysis. Am J Psychiatry. 2000;157(10):1552‐1562. doi:10.1176/appi.ajp.157.10.1552 11007705

[4] Sullivan PF , Geschwind DH . Defining the genetic, genomic, cellular, and diagnostic architectures of psychiatric disorders. Cell. 2019;177(1):162‐183. doi:10.1016/j.cell.2019.01.015 30901538 PMC6432948

[5] Kendler KS , Gatz M , Gardner CO , Pedersen NL . Personality and major depression: a swedish longitudinal, population‐based twin study. Arch Gen Psychiatry. 2006;63(10):1113‐1120. doi:10.1001/archpsyc.63.10.1113 17015813

[6] Levey DF , Stein MB , Wendt FR , et al. GWAS of depression phenotypes in the million veteran program and meta‐analysis in more than 1.2 million participants yields 178 independent risk loci MedRxiv, 2020.05. 18.20100685. 2020. doi:10.1101/2020.05.18.20100685

[7] Howard DM , Adams MJ , Clarke T‐K , et al. Genome‐wide meta‐analysis of depression identifies 102 independent variants and highlights the importance of the prefrontal brain regions. Nat Neurosci. 2019;22(3):343‐352. doi:10.1038/s41593-018-0326-7 30718901 PMC6522363

[8] Nagel M , Jansen PR , Stringer S , et al. Meta‐analysis of genome‐wide association studies for neuroticism in 449,484 individuals identifies novel genetic loci and pathways. Nat Genet. 2018;50(7):920‐927. doi:10.1038/s41588-018-0151-7 29942085

[9] Luciano M , Hagenaars SP , Davies G , et al. Association analysis in over 329,000 individuals identifies 116 independent variants influencing neuroticism. Nat Genet. 2018;50(1):6‐11. doi:10.1038/s41588-017-0013-8 29255261 PMC5985926

[10] Kendler KS , Myers J . The genetic and environmental relationship between major depression and the five‐factor model of personality. Psychol Med. 2010;40(5):801‐806. doi:10.1017/s0033291709991140 19732485

[11] Kotov R , Gamez W , Schmidt F , Watson D . Linking “big” personality traits to anxiety, depressive, and substance use disorders: a meta‐analysis. Psychol Bull. 2010;136(5):768‐821. doi:10.1037/a0020327 20804236

[12] Prince EJ , Siegel DJ , Carroll CP , Sher KJ , Bienvenu OJ . A longitudinal study of personality traits, anxiety, and depressive disorders in young adults. Anxiety Stress Coping. 2021;34(3):299‐307. doi:10.1080/10615806.2020.1845431 33190525 PMC8068574

[13] Vukasović T , Bratko D . Heritability of personality: a meta‐analysis of behavior genetic studies. Psychol Bull. 2015;141(4):769‐785. doi:10.1037/bul0000017 25961374

[14] Jang KL , Livesley WJ , Vernon PA . Heritability of the big five personality dimensions and their facets: a twin study. J Pers. 1996;64(3):577‐591. doi:10.1111/j.1467-6494.1996.tb00522.x 8776880

[15] Visscher PM , Wray NR , Zhang Q , et al. 10 years of GWAS discovery: biology, function, and translation. Am J Hum Genet. 2017;101(1):5‐22. doi:10.1016/j.ajhg.2017.06.005 28686856 PMC5501872

[16] Duncan LE , Keller MC . A critical review of the first 10 years of candidate gene‐by‐environment interaction research in psychiatry. Am J Psychiatry. 2011;168(10):1041‐1049. doi:10.1176/appi.ajp.2011.11020191 21890791 PMC3222234

[17] Assary E , Vincent JP , Keers R , Pluess M . Gene‐environment interaction and psychiatric disorders: review and future directions. Semin Cell Dev Biol. 2018;77:133‐143. doi:10.1016/j.semcdb.2017.10.016 29051054

[18] Musci RJ , Augustinavicius JL , Volk H . Gene‐environment interactions in psychiatry: recent evidence and clinical implications. Curr Psychiatry Rep. 2019;21(9):81. doi:10.1007/s11920-019-1065-5 31410638 PMC7340157

[19] Uher R , Zwicker A . Etiology in psychiatry: embracing the reality of poly‐gene‐environmental causation of mental illness. World Psychiatry. 2017;16(2):121‐129. doi:10.1002/wps.20436 28498595 PMC5428165

[20] Lazarus RS , Folkman S . Stress, Appraisal, and Coping. Springer publishing company; 1984.

[21] Kivimäki M , Jokela M , Nyberg ST , et al. Long working hours and risk of coronary heart disease and stroke: a systematic review and meta‐analysis of published and unpublished data for 603,838 individuals. Lancet. 2015;386(10005):1739‐1746. doi:10.1016/s0140-6736(15)60295-1 26298822

[22] Kivimäki M , Virtanen M , Kawachi I , et al. Long working hours, socioeconomic status, and the risk of incident type 2 diabetes: a meta‐analysis of published and unpublished data from 222 120 individuals. Lancet Diabetes Endocrinol. 2015;3(1):27‐34. doi:10.1016/s2213-8587(14)70178-0 25262544 PMC4286814

[23] Madsen IEH , Nyberg ST , Magnusson Hanson LL , et al. Job strain as a risk factor for clinical depression: systematic review and meta‐analysis with additional individual participant data. Psychol Med. 2017;47(8):1342‐1356. doi:10.1017/s003329171600355x 28122650 PMC5471831

[24] Nillni YI , Nosen E , Williams PA , Tracy M , Coffey SF , Galea S . Unique and related predictors of major depressive disorder, posttraumatic stress disorder, and their comorbidity after hurricane Katrina. J Nerv Ment Dis. 2013;201(10):841‐847. doi:10.1097/NMD.0b013e3182a430a0 24080670 PMC6554025

[25] Galatzer‐Levy IR , Huang SH , Bonanno GA . Trajectories of resilience and dysfunction following potential trauma: A review and statistical evaluation. Clin Psychol Rev. 2018;63:41‐55. doi:10.1016/j.cpr.2018.05.008 29902711

[26] Chrousos GP . Stress and disorders of the stress system. Nat Rev Endocrinol. 2009;5(7):374‐381. doi:10.1038/nrendo.2009.106 19488073

[27] Lahey BB . Public health significance of neuroticism. Am Psychol. 2009;64(4):241‐256. doi:10.1037/a0015309 19449983 PMC2792076

[28] Schneider TR , Rench TA , Lyons JB , Riffle RR . The influence of neuroticism, extraversion and openness on stress responses. Stress Health. 2012;28(2):102‐110. doi:10.1002/smi.1409 22281953

[29] McCrae RR . Controlling neuroticism in the measurement of stress. Stress Med. 1990;6(3):237‐241. doi:10.1002/smi.2460060309

[30] Sharma S , Powers A , Bradley B , Ressler KJ . Gene × environment determinants of stress‐ and anxiety‐related disorders. Annu Rev Psychol. 2016;67:239‐261. doi:10.1146/annurev-psych-122414-033408 26442668 PMC5739029

[31] Smoller JW . The genetics of stress‐related disorders: PTSD, depression, and anxiety disorders. Neuropsychopharmacology. 2016;41(1):297‐319. doi:10.1038/npp.2015.266 26321314 PMC4677147

[32] Werme J , van der Sluis S , Posthuma D , de Leeuw CA . Genome‐wide gene‐environment interactions in neuroticism: an exploratory study across 25 environments. Transl Psychiatry. 2021;11(1):180. doi:10.1038/s41398-021-01288-9 33753719 PMC7985503

[33] Arnau‐Soler A , Adams MJ , Hayward C , Thomson PA . Genome‐wide interaction study of a proxy for stress‐sensitivity and its prediction of major depressive disorder. PloS One. 2018;13(12):e0209160. doi:10.1371/journal.pone.0209160 30571770 PMC6301766

[34] Fang Y , Scott L , Song P , Burmeister M , Sen S . Genomic prediction of depression risk and resilience under stress. Nat Hum Behav. 2020;4(1):111‐118. doi:10.1038/s41562-019-0759-3 31659322 PMC6980948

[35] Mullins N , Power RA , Fisher HL , et al. Polygenic interactions with environmental adversity in the aetiology of major depressive disorder. Psychol Med. 2016;46(4):759‐770. doi:10.1017/S0033291715002172 26526099 PMC4754832

[36] Lehto K , Karlsson I , Lundholm C , Pedersen NL . Genetic risk for neuroticism predicts emotional health depending on childhood adversity. Psychol Med. 2018;49(2):260‐267. doi:10.1017/s0033291718000715 29576022

[37] Li JJ , Hilton EC , Lu Q , Hong J , Greenberg JS , Mailick MR . Validating psychosocial pathways of risk between neuroticism and late life depression using a polygenic score approach. J Abnorm Psychol. 2019;128(3):200‐211. doi:10.1037/abn0000419 30829503 PMC6462143

[38] Choi SW , O'Reilly PF . PRSice‐2: polygenic risk score software for biobank‐scale data. Gigascience. 2019;8(7):giz082. doi:10.1093/gigascience/giz082 31307061 PMC6629542

[39] Wray NR , Lee SH , Mehta D , Vinkhuyzen AA , Dudbridge F , Middeldorp CM . Research review: polygenic methods and their application to psychiatric traits. J Child Psychol Psychiatry. 2014;55(10):1068‐1087. doi:10.1111/jcpp.12295 25132410

[40] Iyegbe C , Campbell D , Butler A , Ajnakina O , Sham P . The emerging molecular architecture of schizophrenia, polygenic risk scores and the clinical implications for GxE research. Soc Psychiatry Psychiatr Epidemiol. 2014;49(2):169‐182. doi:10.1007/s00127-014-0823-2 24435092

[41] Harden KP . “reports of my death were greatly exaggerated”: behavior genetics in the postgenomic era. Annu Rev Psychol. 2021;72:37‐60. doi:10.1146/annurev-psych-052220-103822 32898465 PMC10372814

[42] Dudbridge F . Power and predictive accuracy of polygenic risk scores. PLoS Genet. 2013;9(3):e1003348. doi:10.1371/journal.pgen.1003348 23555274 PMC3605113

[43] Musliner KL , Andersen KK , Agerbo E , et al. Polygenic liability, stressful life events and risk for secondary‐treated depression in early life: a nationwide register‐based case‐cohort study. Psychol Med. 2021;53:217‐226. doi:10.1017/s0033291721001410 33949298

[44] Arnau‐Soler A , Adams MJ , Clarke TK , et al. A validation of the diathesis‐stress model for depression in generation Scotland. Transl Psychiatry. 2019;9(1):25. doi:10.1038/s41398-018-0356-7 30659167 PMC6338746

[45] Coleman JRI , Peyrot WJ , Purves KL , et al. Genome‐wide gene‐environment analyses of major depressive disorder and reported lifetime traumatic experiences in UK biobank. Mol Psychiatry. 2020;25(7):1430‐1446. doi:10.1038/s41380-019-0546-6 31969693 PMC7305950

[46] Domingue BW , Liu H , Okbay A , Belsky DW . Genetic heterogeneity in depressive symptoms following the death of a spouse: polygenic score analysis of the U.S. health and retirement study. Am J Psychiatry. 2017;174(10):963‐970. doi:10.1176/appi.ajp.2017.16111209 28335623 PMC5610918

[47] Musliner KL , Seifuddin F , Judy JA , Pirooznia M , Goes FS , Zandi PP . Polygenic risk, stressful life events and depressive symptoms in older adults: a polygenic score analysis. Psychol Med. 2015;45(8):1709‐1720. doi:10.1017/s0033291714002839 25488392 PMC4412793

[48] Peyrot WJ , Van der Auwera S , Milaneschi Y , et al. Does childhood trauma moderate polygenic risk for depression? A meta‐analysis of 5765 subjects from the psychiatric genomics consortium. Biol Psychiatry. 2018;84(2):138‐147. doi:10.1016/j.biopsych.2017.09.009 29129318 PMC5862738

[49] Colman I , Kingsbury M , Garad Y , et al. Consistency in adult reporting of adverse childhood experiences. Psychol Med. 2016;46(3):543‐549. doi:10.1017/s0033291715002032 26511669

[50] Monroe SM , Reid MW . Gene‐environment interactions in depression research: genetic polymorphisms and life‐stress polyprocedures. Psychol Sci. 2008;19(10):947‐956. doi:10.1111/j.1467-9280.2008.02181.x 19000200

[51] Zammit S , Owen MJ . Stressful life events, 5‐HTT genotype and risk of depression. Br J Psychiatry. 2006;188:199‐201. doi:10.1192/bjp.bp.105.020644 16507957

[52] Trull TJ , Ebner‐Priemer U . The role of ambulatory assessment in psychological science. Curr Dir Psychol Sci. 2014;23(6):466‐470. doi:10.1177/0963721414550706 25530686 PMC4269226

[53] Fox E , Beevers CG . Differential sensitivity to the environment: contribution of cognitive biases and genes to psychological wellbeing. Mol Psychiatry. 2016;21(12):1657‐1662. doi:10.1038/mp.2016.114 27431291 PMC5075581

[54] Conner TS , Barrett LF . Trends in ambulatory self‐report: the role of momentary experience in psychosomatic medicine. Psychosom Med. 2012;74(4):327‐337. doi:10.1097/PSY.0b013e3182546f18 22582330 PMC3372543

[55] Monninger M , Aggensteiner P‐M , Pollok TM , et al. Real‐time individual benefit from social interactions before and during the lockdown: the crucial role of personality, neurobiology and genes. Transl Psychiatry. 2022;12(1):28. doi:10.1038/s41398-022-01799-z 35064105 PMC8777449

[56] Pries L‐K , Klingenberg B , Menne‐Lothmann C , et al. Polygenic liability for schizophrenia and childhood adversity influences daily‐life emotion dysregulation and psychosis proneness. Acta Psychiatr Scand. 2020;141(5):465‐475. doi:10.1111/acps.13158 32027017 PMC7318228

[57] Schick A , van Winkel R , Lin BD , et al. Polygenic risk, familial liability and stress reactivity in psychosis: an experience sampling study. Psychol Med. 2022;53:2798‐2807. doi:10.1017/s0033291721004761 34991751 PMC10235643

[58] Giglberger M , Peter HL , Kraus E , et al. Daily life stress and the cortisol awakening response over a 13‐months stress period—findings from the LawSTRESS project. Psychoneuroendocrinology. 2022;141:105771. doi:10.1016/j.psyneuen.2022.105771 35489313

[59] Peter HL , Giglberger M , Frank J , et al. The association between genetic variability in the NPS/NPSR1 system and chronic stress responses: a gene‐environment‐(quasi‐) experiment. Psychoneuroendocrinology. 2022;144:105883. doi:10.1016/j.psyneuen.2022.105883 35914393

[60] Pruessner JC , Kirschbaum C , Meinlschmid G , Hellhammer DH . Two formulas for computation of the area under the curve represent measures of total hormone concentration versus time‐dependent change. Psychoneuroendocrinology. 2003;28(7):916‐931. doi:10.1016/s0306-4530(02)00108-7 12892658

[61] Stalder T , Kirschbaum C , Kudielka BM , et al. Assessment of the cortisol awakening response: expert consensus guidelines. Psychoneuroendocrinology. 2016;63:414‐432. doi:10.1016/j.psyneuen.2015.10.010 26563991

[62] Wilhelm I , Born J , Kudielka BM , Schlotz W , Wüst S . Is the cortisol awakening rise a response to awakening? Psychoneuroendocrinology. 2007;32(4):358‐366. doi:10.1016/j.psyneuen.2007.01.008 17408865

[63] Wüst S , Federenko I , Hellhammer DH , Kirschbaum C . Genetic factors, perceived chronic stress, and the free cortisol response to awakening. Psychoneuroendocrinology. 2000;25(7):707‐720. doi:10.1016/s0306-4530(00)00021-4 10938450

[64] Kupper N , de Geus EJ , van den Berg M , Kirschbaum C , Boomsma DI , Willemsen G . Familial influences on basal salivary cortisol in an adult population. Psychoneuroendocrinology. 2005;30(9):857‐868. doi:10.1016/j.psyneuen.2005.04.003 15949896

[65] Bakermans‐Kranenburg MJ , van IJzendoorn MH . The hidden efficacy of interventions: gene×environment experiments from a differential susceptibility perspective. Annu Rev Psychol. 2015;66:381‐409. doi:10.1146/annurev-psych-010814-015407 25148854

[66] van IJzendoorn MH , Bakermans‐Kranenburg MJ , Belsky J , et al. Gene‐by‐environment experiments: a new approach to finding the missing heritability. Nat Rev Genet. 2011;12(12):881. doi:10.1038/nrg2764-c1 22094952

[67] Adams MJ , Howard DM , Luciano M , et al. Genetic stratification of depression by neuroticism: revisiting a diagnostic tradition. Psychol Med. 2020;50(15):2526‐2535. doi:10.1017/s0033291719002629 31576797 PMC7737042

[68] Kudielka BM , Broderick JE , Kirschbaum C . Compliance with saliva sampling protocols: electronic monitoring reveals invalid cortisol daytime profiles in noncompliant subjects. Psychosom Med. 2003;65(2):313‐319. doi:10.1097/01.PSY.0000058374.50240.BF 12652000

[69] Broderick JE , Arnold D , Kudielka BM , Kirschbaum C . Salivary cortisol sampling compliance: comparison of patients and healthy volunteers. Psychoneuroendocrinology. 2004;29(5):636‐650. doi:10.1016/s0306-4530(03)00093-3 15041086

[70] Dressendörfer RA , Kirschbaum C , Rohde W , Stahl F , Strasburger CJ . Synthesis of a cortisol‐biotin conjugate and evaluation as a tracer in an immunoassay for salivary cortisol measurement. J Steroid Biochem Mol Biol. 1992;43(7):683‐692. doi:10.1016/0960-0760(92)90294-s 1472460

[71] *SoSci Survey (version 2.500‐i)* [computer program]. 2014.

[72] Herrmann‐Lingen C , Buss U , Snaith RP . Hospital Anxiety and Depression Scale—Deutsche Version (HADS‐D). 3rd ed. Verlag Hans Huber; 2011.

[73] Miller SA , Dykes DD , Polesky HF . A simple salting out procedure for extracting DNA from human nucleated cells. Nucleic Acids Res. 1988;16(3):1215. doi:10.1093/nar/16.3.1215 3344216 PMC334765

[74] Chang CC , Chow CC , Tellier LC , Vattikuti S , Purcell SM , Lee JJ . Second‐generation PLINK: rising to the challenge of larger and richer datasets. Gigascience. 2015;4(1):7. doi:10.1186/s13742-015-0047-8 25722852 PMC4342193

[75] Loh PR , Danecek P , Palamara PF , et al. Reference‐based phasing using the haplotype reference consortium panel. Nat Genet. 2016;48(11):1443‐1448. doi:10.1038/ng.3679 27694958 PMC5096458

[76] Das S , Forer L , Schönherr S , et al. Next‐generation genotype imputation service and methods. Nat Genet. 2016;48(10):1284‐1287. doi:10.1038/ng.3656 27571263 PMC5157836

[77] Auton A , Brooks LD , Durbin RM , et al. A global reference for human genetic variation. Nature. 2015;526(7571):68‐74. doi:10.1038/nature15393 26432245 PMC4750478

[78] Ge T , Chen CY , Ni Y , Feng YA , Smoller JW . Polygenic prediction via Bayesian regression and continuous shrinkage priors. Nat Commun. 2019;10(1):1776. doi:10.1038/s41467-019-09718-5 30992449 PMC6467998

[79] Yengo L , Sidorenko J , Kemper KE , et al. Meta‐analysis of genome‐wide association studies for height and body mass index in ∼700000 individuals of european ancestry. Hum Mol Genet. 2018;27(20):3641‐3649. doi:10.1093/hmg/ddy271 30124842 PMC6488973

[80] R: A Language and Environment for Statistical Computing [Computer Program]. R Foundation for Statistical Computing; 2020.

[81] Brooks ME , Kristensen K , Van Benthem KJ , et al. glmmTMB balances speed and flexibility among packages for zero‐inflated generalized linear mixed modeling. R J. 2017;9(2):378‐400. doi:10.32614/RJ-2017-066

[82] Pinheiro J , Bates D , DebRoy S , Sarkar D , R Core Team . nlme: linear and nonlinear mixed effects models. R package version 3.1‐139. In:2021. 2019.

[83] Keller MC . Gene × environment interaction studies have not properly controlled for potential confounders: the problem and the (simple) solution. Biol Psychiatry. 2014;75(1):18‐24. doi:10.1016/j.biopsych.2013.09.006 24135711 PMC3859520

[84] Nakagawa S , Schielzeth H . A general and simple method for obtaining R2 from generalized linear mixed‐effects models. Methods Ecol Evol. 2013;4(2):133‐142. doi:10.1111/j.2041-210x.2012.00261.x

[85] Kendler KS , Karkowski LM , Prescott CA . Causal relationship between stressful life events and the onset of major depression. Am J Psychiatry. 1999;156(6):837‐841. doi:10.1176/ajp.156.6.837 10360120

[86] Kessler RC . The effects of stressful life events on depression. Annu Rev Psychol. 1997;48:191‐214. doi:10.1146/annurev.psych.48.1.191 9046559

[87] Dunn EC , Brown RC , Dai Y , et al. Genetic determinants of depression: recent findings and future directions. Harv Rev Psychiatry. 2015;23(1):1‐18. doi:10.1097/hrp.0000000000000054 25563565 PMC4309382

[88] Jylhä P , Isometsä E . The relationship of neuroticism and extraversion to symptoms of anxiety and depression in the general population. Depress Anxiety. 2006;23(5):281‐289. doi:10.1002/da.20167 16688731

[89] Cai N , Revez JA , Adams MJ , et al. Minimal phenotyping yields genome‐wide association signals of low specificity for major depression. Nat Genet. 2020;52(4):437‐447. doi:10.1038/s41588-020-0594-5 32231276 PMC7906795

[90] Mitchell BL , Thorp JG , Wu Y , et al. Polygenic risk scores derived from varying definitions of depression and risk of depression. JAMA Psychiatry. 2021;78(10):1152‐1160. doi:10.1001/jamapsychiatry.2021.1988 34379077 PMC8358814

[91] Rotenstein LS , Ramos MA , Torre M , et al. Prevalence of depression, depressive symptoms, and suicidal ideation among medical students: a systematic review and meta‐analysis. JAMA. 2016;316(21):2214‐2236. doi:10.1001/jama.2016.17324 27923088 PMC5613659

[92] O'Flynn J , Dinan TG , Kelly JR . Examining stress: an investigation of stress, mood and exercise in medical students. Ir J Psychol Med. 2018;35(1):63‐68. doi:10.1017/ipm.2017.54 30115207

[93] Rietschel L , Zhu G , Kirschbaum C , et al. Perceived stress has genetic influences distinct from neuroticism and depression. Behav Genet. 2013;44(6):639‐645. doi:10.1007/s10519-013-9636-4 24366676

[94] Jacobs N , van Os J , Derom C , Thiery E , Delespaul P , Wichers M . Neuroticism explained? From a non‐informative vulnerability marker to informative person‐context interactions in the realm of daily life. Br J Clin Psychol. 2011;50(1):19‐32. doi:10.1348/014466510x491397 21332518

[95] Thorp JG , Campos AI , Grotzinger AD , et al. Symptom‐level modelling unravels the shared genetic architecture of anxiety and depression. Nat Hum Behav. 2021;5(10):1432‐1442. doi:10.1038/s41562-021-01094-9 33859377

[96] Rietschel L , Streit F , Zhu G , et al. Hair cortisol in twins: heritability and genetic overlap with psychological variables and stress‐system genes. Sci Rep. 2017;7(1):15351. doi:10.1038/s41598-017-11852-3 29127340 PMC5703444

[97] de Moor MH , van den Berg SM , Verweij KJ , et al. Meta‐analysis of genome‐wide association studies for neuroticism, and the polygenic association with major depressive disorder. JAMA Psychiatry. 2015;72(7):642‐650. doi:10.1001/jamapsychiatry.2015.0554 25993607 PMC4667957

[98] Lobo JJ , McLean SA , Tungate AS , et al. Polygenic risk scoring to assess genetic overlap and protective factors influencing posttraumatic stress, depression, and chronic pain after motor vehicle collision trauma. Transl Psychiatry. 2021;11(1):359. doi:10.1038/s41398-021-01486-5 34226500 PMC8257703

[99] Fahrenberg J . Psychophysiologische Aktivierungsforschung: Ein Beitrag zu den Grundlagen der multivariaten Emotions‐u. Stress‐Theorie. Munich: Minerva. 1979.

[100] Campbell J , Ehlert U . Acute psychosocial stress: does the emotional stress response correspond with physiological responses? Psychoneuroendocrinology. 2012;37(8):1111‐1134. doi:10.1016/j.psyneuen.2011.12.010 22260938

[101] Wray NR , Lin T , Austin J , et al. From basic science to clinical application of polygenic risk scores. JAMA Psychiatry. 2021;78(1):101‐109. doi:10.1001/jamapsychiatry.2020.3049 32997097

[102] Torkamani A , Wineinger NE , Topol EJ . The personal and clinical utility of polygenic risk scores. Nat Rev Genet. 2018;19(9):581‐590. doi:10.1038/s41576-018-0018-x 29789686

[103] Wang M , Hu K , Fan L , et al. Predicting treatment response in schizophrenia with magnetic resonance imaging and polygenic risk score. Front Genet. 2022;13:848205. doi:10.3389/fgene.2022.848205 35186051 PMC8847599

