# Supplementary material for: Association of polygenic scores for depression and neuroticism with perceived stress in daily life during a long‐lasting stress period
Source: Genes Brain Behav. 2023 Oct 24;22(6):e12872. doi: 10.1111/gbb.12872 (PMC10733580; doi:10.1111/gbb.12872)
Supplement: Supplementary file 1 — Data S1: Supporting Information. [file GBB-22-e12872-s001.docx]

SUPPLEMENTS: Association of polygenic scores for depression and neuroticism with perceived stress in daily life during a long-lasting stress period

Hannah L. Peter^1^, Marina Giglberger^1^, Fabian Streit^2^, Josef Frank^2^, Ludwig Kreuzpointner^1^, Marcella Rietschel^2^, Brigitte M. Kudielka^1^, Stefan Wüst*^1^

^1^ Institute of Psychology, University of Regensburg, Germany

^2^ Department of Genetic Epidemiology in Psychiatry, Central Institute of Mental Health, University of Mannheim, Germany

**Short title:** Polygenic scores and chronic stress responses

**Supplemental Results**

[AA stress scale 2](#_Toc114061120)

[Depression symptoms 5](#_Toc114061121)

[AUCg 9](#_Toc114061122)

[AUCi 15](#_Toc114061123)

[Supplemental References 18](#_Toc114061124)

# Descriptives of the dependent variables

TABLE S1. Mean ± *SD* of AA stress scale for each timepoint divided by group.

|  |  |  | *n* | Mean | *SD* |
| --- | --- | --- | --- | --- | --- |
| AA stress scale | timepoint 1 | SG | 218 | 14.78 | 6.22 |
|  |  | CG | 214 | 13.08 | 5.62 |
|  | timepoint 2 | SG | 195 | 17.11 | 6.48 |
|  |  | CG | 209 | 13.86 | 6.03 |
|  | timepoint 3 | SG | 193 | 19.34 | 7.30 |
|  |  | CG | 208 | 14.03 | 6.25 |
|  | timepoint 4 | SG | 192 | 18.05 | 7.63 |
|  |  | CG | 206 | 14.11 | 6.37 |

TABLE S2. Mean ± *SD* of depression symptoms (HADS) for each timepoint divided by group.

|  |  |  | *n* | Mean | *SD* |
| --- | --- | --- | --- | --- | --- |
| Depression symptoms (HADS) | timepoint 1 | SG | 218 | 3.60 | 2.75 |
|  |  | CG | 214 | 3.95 | 3.06 |
|  | timepoint 2 | SG | 194 | 5.75 | 3.63 |
|  |  | CG | 209 | 4.29 | 3.07 |
|  | timepoint 3 | SG | 188 | 6.70 | 3.95 |
|  |  | CG | 209 | 4.27 | 3.22 |

TABLE S3. Mean ± *SD* of area under the curve with respect to the increase (*AUCi*) for each timepoint divided by group.

|  |  |  | *n* | Mean | *SD* |
| --- | --- | --- | --- | --- | --- |
| *AUCi* | timepoint 1 | SG | 95 | 153.96 | 145.95 |
|  |  | CG | 101 | 180.49 | 131.87 |
|  | timepoint 2 | SG | 84 | 150.70 | 128.64 |
|  |  | CG | 98 | 134.73 | 141.48 |
|  | timepoint 3 | SG | 84 | 127.72 | 135.19 |
|  |  | CG | 97 | 151.77 | 147.26 |
|  | timepoint 4 | SG | 83 | 56.10 | 145.59 |
|  |  | CG | 96 | 144.27 | 138.20 |

TABLE S4. Mean ± *SD* of area under the curve with respect to the ground (*AUCg*) for each timepoint divided by group.

|  |  |  | *n* | Mean | *SD* |
| --- | --- | --- | --- | --- | --- |
| *AUCg* | timepoint 1 | SG | 95 | 493.78 | 173.55 |
|  |  | CG | 101 | 497.89 | 185.69 |
|  | timepoint 2 | SG | 84 | 451.22 | 162.44 |
|  |  | CG | 98 | 482.71 | 176.20 |
|  | timepoint 3 | SG | 84 | 454.09 | 159.68 |
|  |  | CG | 97 | 496.27 | 196.03 |
|  | timepoint 4 | SG | 83 | 417.41 | 160.84 |
|  |  | CG | 96 | 468.22 | 180.07 |

# Distribution and descriptives of the polygenic scores

## Depression-PGS

TABLE S5. Mean ± *SD* of the polygenic score for depression divided by group and cohort.

|  | *n* | Mean | *SD* |
| --- | --- | --- | --- |
| Both groups | 432 | -2.7787 e-04 | 2.6193e-05 |
| Only stress group | 218 | -2.7717 e-04 | 2.7392e-05 |
| Only control group | 214 | -2.7858 e-04 | 2.4956e-05 |
| Only Cohort A | 196 | -2.7849 e-04 | 2.6883e-05 |
| Only Cohort B | 236 | -2.7735 e-04 | 2.5651e-05 |


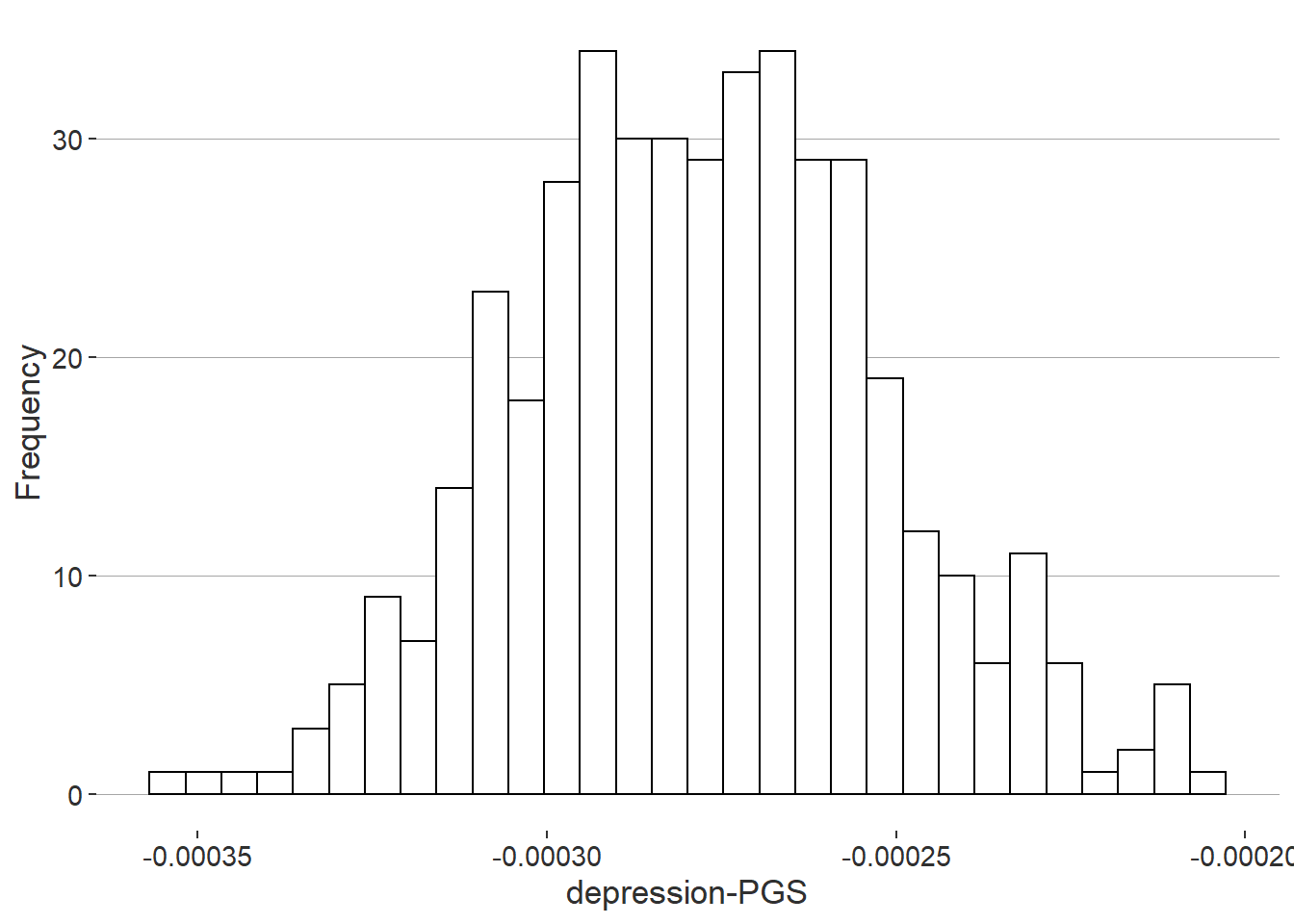


Figure S1. Histogram of the polygenic score for depression.


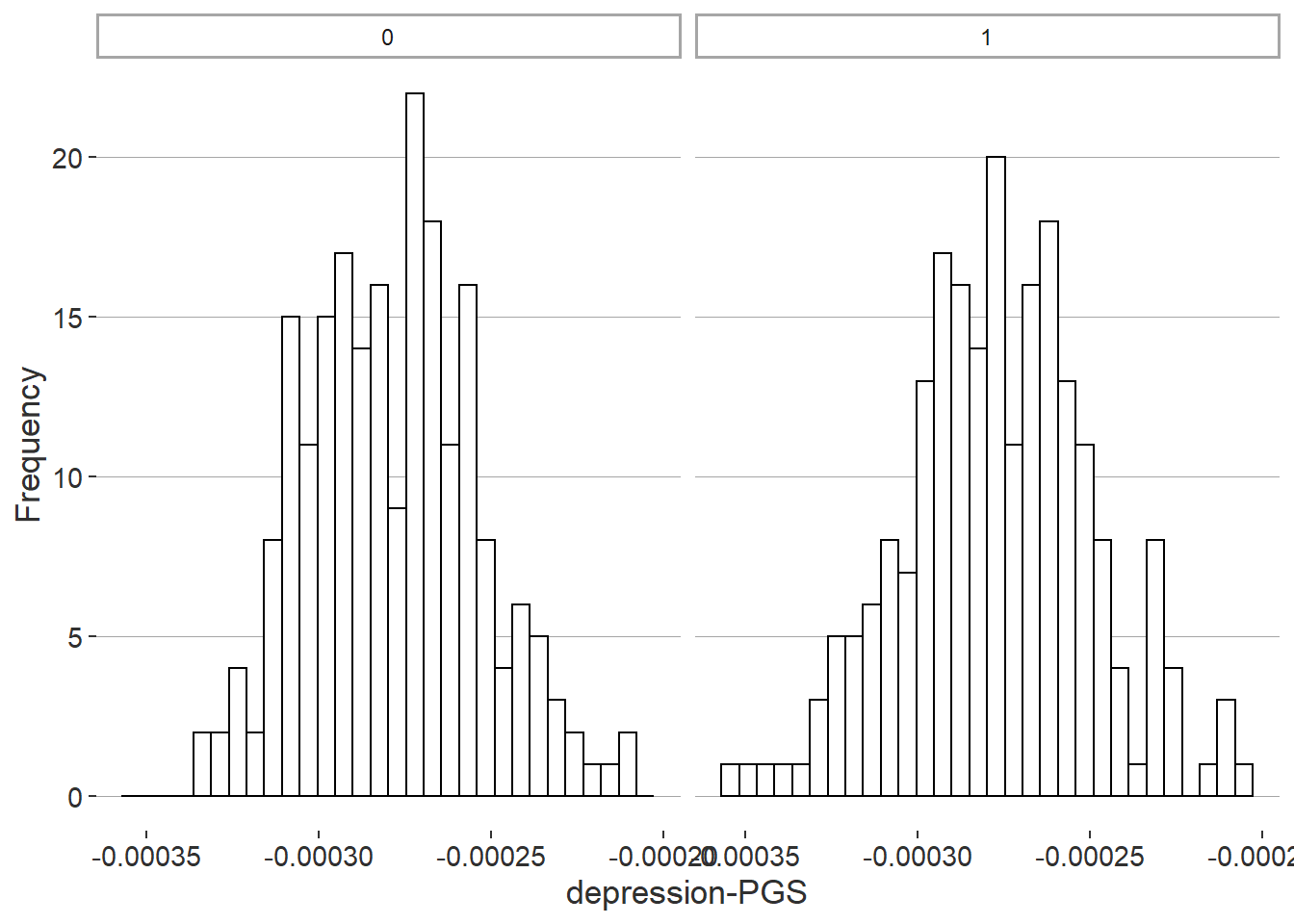


Figure S2. Histogram of the polygenic score for depression divided by group (0 = control group; 1 = stress group).


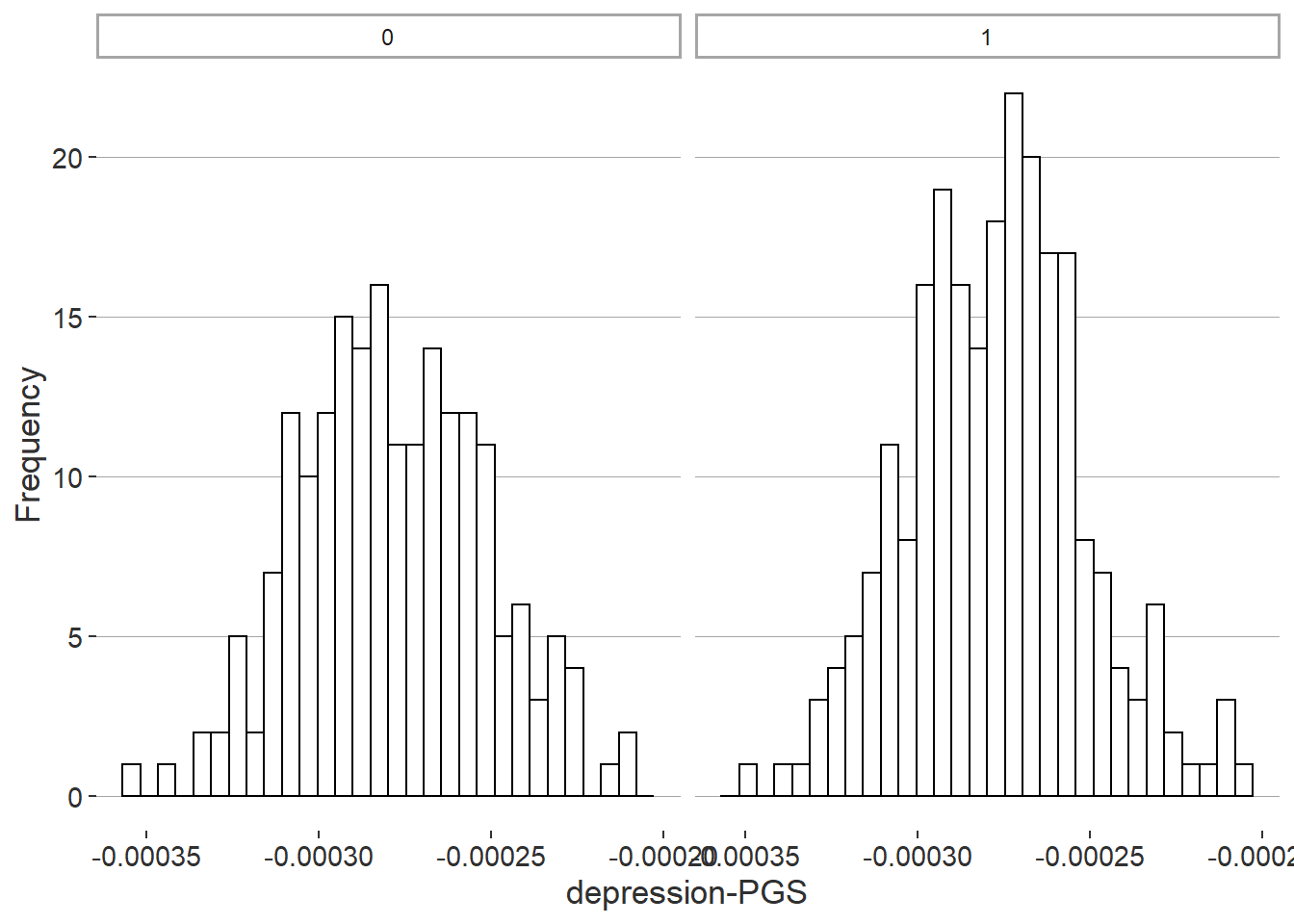


Figure S3. Histogram of the polygenic score for depression divided by cohort (0 = cohort A; 1 = cohort B).

## Neuroticism-PGS

TABLE S6. Mean ± *SD* of the polygenic score for neuroticism divided by group and cohort.

|  | *n* | Mean | *SD* |
| --- | --- | --- | --- |
| Both groups | 432 | -0.010774 | 0.0032348 |
| Only stress group | 218 | -0.010749 | 0.0032279 |
| Only control group | 214 | -0.010799 | 0.0032491 |
| Only Cohort A | 196 | -0.010506 | 0.0032526 |
| Only Cohort B | 236 | -0.010996 | 0.0032097 |


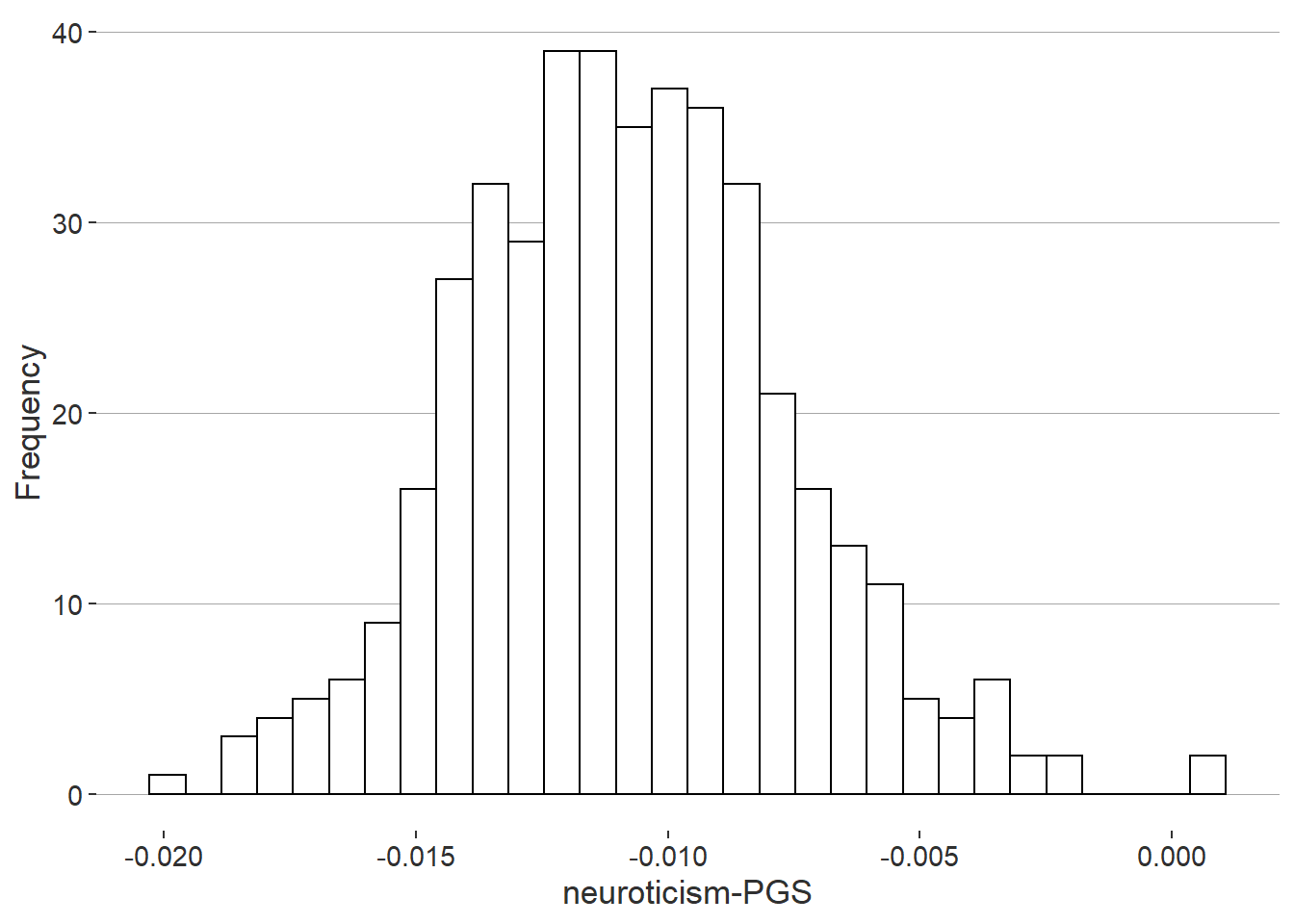


Figure S4. Histogram of the polygenic score for neuroticism.


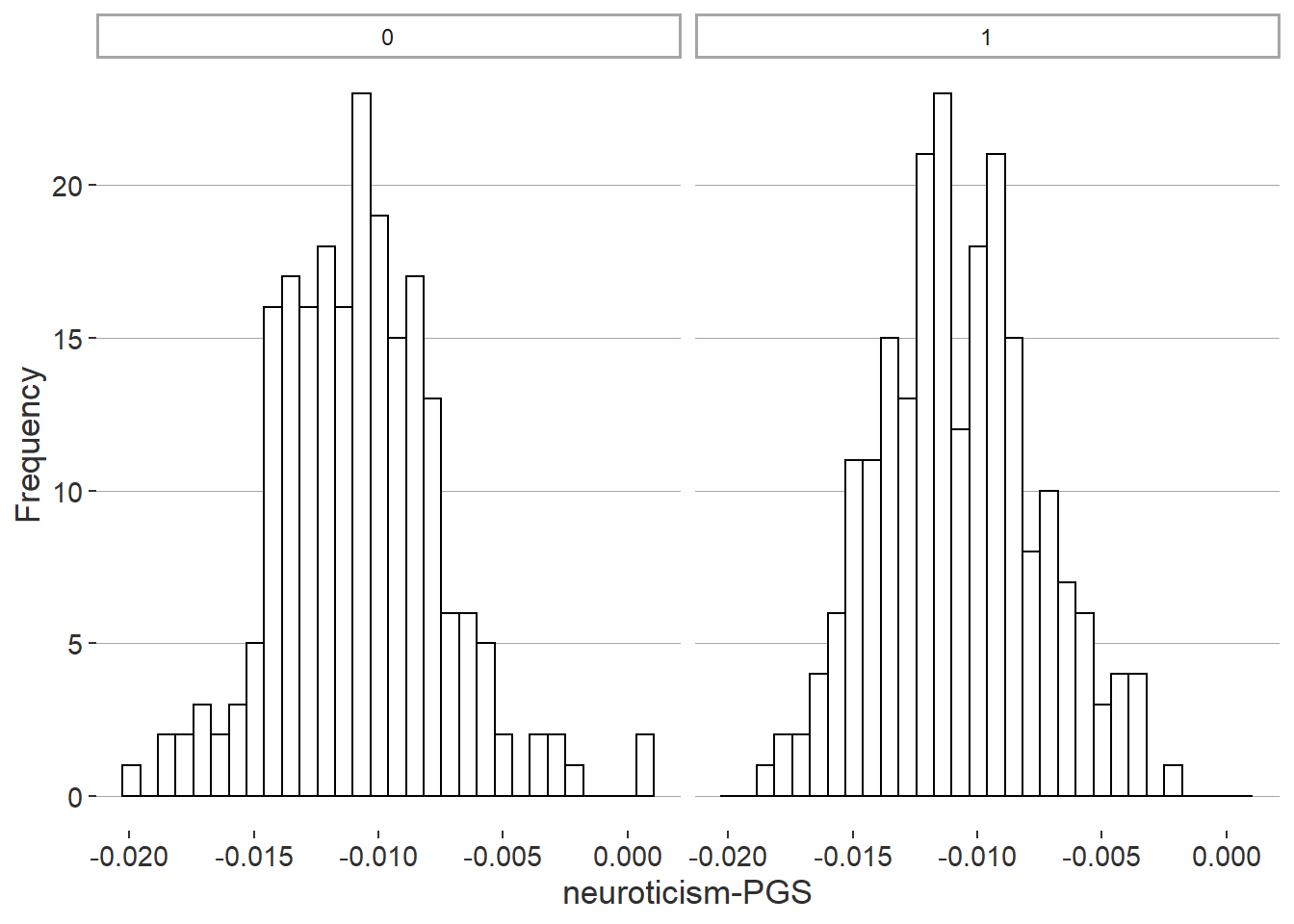


Figure S5. Histogram of the polygenic score for neuroticism divided by group (0 = control group; 1 = stress group).


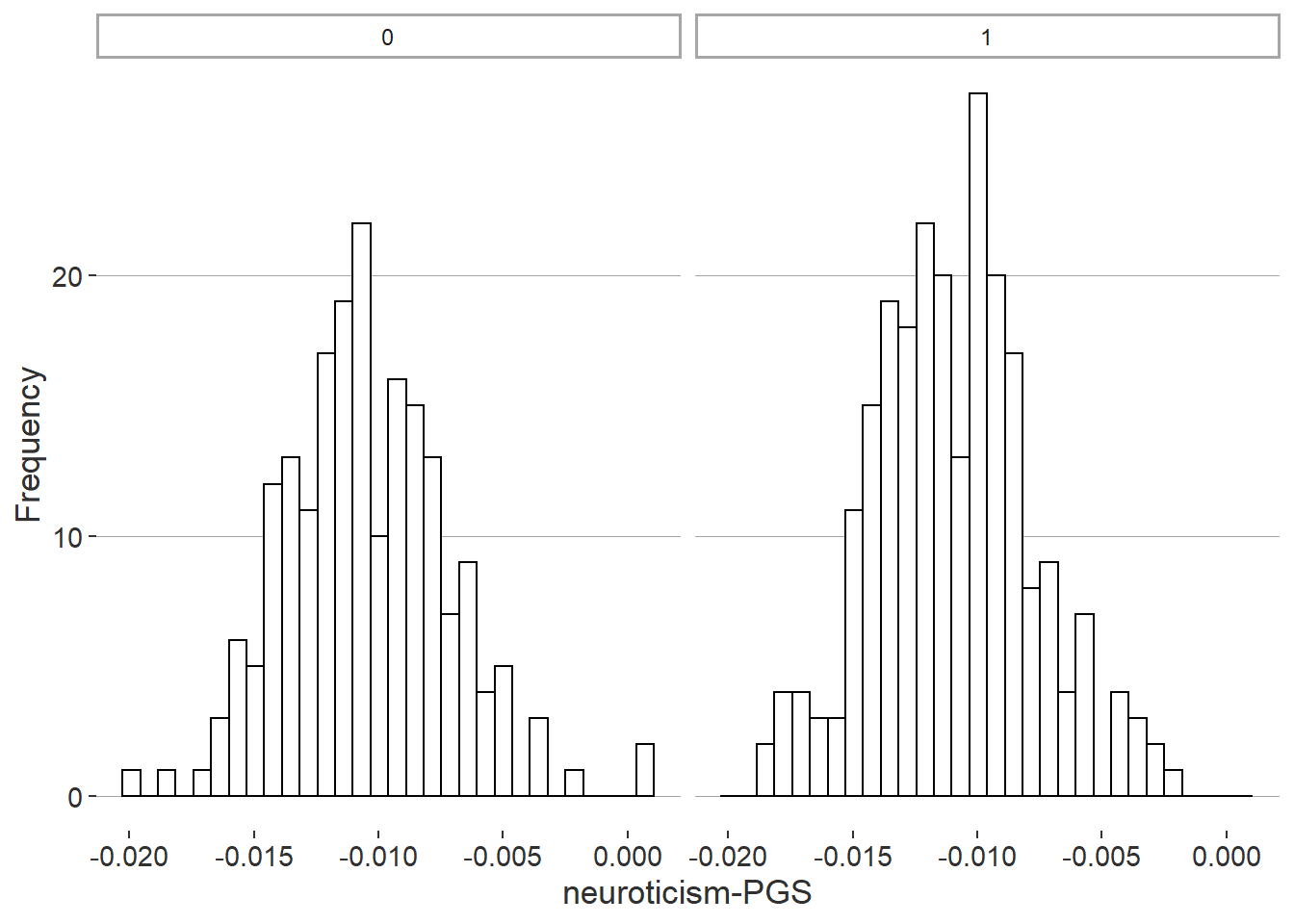


Figure S6. Histogram of the polygenic score for neuroticism divided by cohort (0 = cohort A; 1 = cohort B).

# AA stress scale

|  | **group.model^a^** | | |  | **DEP-PGS.model^b^** | | |  | **PC.model^c^** | | |
| --- | --- | --- | --- | --- | --- | --- | --- | --- | --- | --- | --- |
|  | Estimates | *SE* | *p* |  | Estimates | *SE* | *p* |  | Estimates | *SE* | *p* |
| Intercept | 2.46 | 0.04 | **< .001** |  | 2.46 | 0.04 | **< .001** |  | 2.48 | 0.05 | **< .001** |
| Timepoint | .07 | 0.01 | **.001** |  | .07 | 0.01 | **.001** |  | .07 | 0.01 | **< .001** |
| Timepoint^2^ | -.02 | 0.00 | **< .001** |  | -.02 | 0.00 | **< .001** |  | -.02 | 0.00 | **< .001** |
| Women (vs. men) | .07 | 0.03 | .054 |  | .07 | 0.03 | .062 |  | .03 | 0.05 | .577 |
| Cohort B (vs. cohort A) | .17 | 0.03 | **< .001** |  | .17 | 0.03 | **< .001** |  | .20 | 0.04 | **< .001** |
| SG (vs. CG) | .10 | 0.03 | **.003** |  | .09 | 0.03 | **.003** |  | .07 | 0.07 | .323 |
| Timepoint x SG | .18 | 0.02 | **< .001** |  | .18 | 0.02 | **< .001** |  | .18 | 0.02 | **< .001** |
| Timepoint^2^ x SG | -.04 | 0.00 | **< .001** |  | -.04 | 0.00 | **< .001** |  | -.04 | 0.00 | **< .001** |
| PGS |  |  |  |  | -.01 | 0.02 | .586 |  | -.01 | 0.04 | .830 |
| PGS x SG |  |  |  |  | .04 | 0.03 | .201 |  | .04 | 0.03 | .194 |
| PGS x timepoint |  |  |  |  | -.02 | 0.01 | .171 |  | -.02 | 0.01 | .171 |
| PGS x timepoint^2^ |  |  |  |  | .01 | 0.00 | **.018** |  | .01 | 0.00 | **.018** |
| PGS x timepoint x SG |  |  |  |  | .02 | 0.02 | .248 |  | .02 | 0.02 | .248 |
| PGS x timepoint^2^ x SG |  |  |  |  | -.01 | 0.00 | **.047** |  | -.01 | 0.00 | **.048** |
| PC1 |  |  |  |  |  |  |  |  | -.03 | 0.47 | .952 |
| PC2 |  |  |  |  |  |  |  |  | -.38 | 0.51 | .453 |
| PC3 |  |  |  |  |  |  |  |  | .02 | 0.48 | .974 |
| PC4 |  |  |  |  |  |  |  |  | -.42 | 0.49 | .389 |
| PC5 |  |  |  |  |  |  |  |  | -.23 | 0.49 | .645 |
| SG x PC1 |  |  |  |  |  |  |  |  | -.46 | 0.66 | .487 |
| SG x PC2 |  |  |  |  |  |  |  |  | -.44 | 0.70 | .526 |
| SG x PC3 |  |  |  |  |  |  |  |  | .02 | 0.68 | .976 |
| SG x PC4 |  |  |  |  |  |  |  |  | .31 | 0.70 | .655 |
| SG x PC5 |  |  |  |  |  |  |  |  | .35 | 0.69 | .612 |
| SG x Women |  |  |  |  |  |  |  |  | .08 | 0.07 | .255 |
| SG x Cohort B |  |  |  |  |  |  |  |  | -.06 | 0.06 | .289 |
| PGS x PC1 |  |  |  |  |  |  |  |  | .02 | 0.33 | .948 |
| PGS x PC2 |  |  |  |  |  |  |  |  | -.27 | 0.35 | .445 |
| PGS x PC3 |  |  |  |  |  |  |  |  | .14 | 0.34 | .688 |
| PGS x PC4 |  |  |  |  |  |  |  |  | -.50 | 0.33 | .126 |
| PGS x PC5 |  |  |  |  |  |  |  |  | .12 | 0.35 | .743 |
| PGS x Women |  |  |  |  |  |  |  |  | -.02 | 0.03 | .629 |
| PGS x Cohort B |  |  |  |  |  |  |  |  | .01 | 0.03 | .755 |
| AIC | 69670.06 | | |  | 69673.43 | | |  | 69699.49 | | |
| BIC | 69751.59 | | |  | 69799.43 | | |  | 69966.31 | | |
| Log-likelihood | -34824.03 | | |  | -34819.71 | | |  | -34813.75 | | |
| Observations | 12230 | | |  | 12230 | | |  | 12230 | | |
| *n* | 432 | | |  | 432 | | |  | 432 | | |
| *SD*: participant (Intercept) | 0.31 | | |  | 0.31 | | |  | 0.31 | | |
| *SD*: timepoint | 0.11 | | |  | 0.11 | | |  | 0.11 | | |
| Correlation: participant x timepoint | -.27 | | |  | -.27 | | |  | -.27 | | |
| Marginal *R^2^* | 0.1005 | | |  | 0.1032 | | |  | 0.1262 | | |
| Conditional *R^2^* | 0.6612 | | |  | 0.6612 | | |  | 0.6649 | | |

TABLE S7. Parameter estimates for overall effects of the models with perceived stress as dependent variable and the polygenic score for depression as predictor.

Abbreviations: CG = Control group; PC = Principal component; PGS = Polygenic score; *SD* = Standard deviation; *SE* = Standard error; SG = Stress group.

^a^The group.model consists of a random intercept for participant, a random slope for the linear time trend and the fixed effects for the linear and quadratic time trend, group and its interactions with the time trends as well as the covariates sex and cohort.

^b^The PGS.model includes the additional fixed effects PGS, PGS in interaction with group as well as with the linear and quadratic time trend, and the three-way interaction of the PGS, group and the time trends.

^c^In the PC.model the covariates PC1 to 5 are added as well as the interactions PGS x covariates and group x covariates.

TABLE S8. Parameter estimates for overall effects of the models with perceived stress as dependent variable and the polygenic score for neuroticism as predictor.

|  | **group.model^a^** | | |  | **NEU-PGS.model^b^** | | |  | **PC.model^c^** | | |
| --- | --- | --- | --- | --- | --- | --- | --- | --- | --- | --- | --- |
|  | Estimates | *SE* | *p* |  | Estimates | *SE* | *p* |  | Estimates | *SE* | *p* |
| Intercept | 2.46 | 0.04 | **< .001** |  | 2.46 | 0.04 | **< .001** |  | 2.46 | 0.05 | **< .001** |
| Timepoint | .07 | 0.01 | **< .001** |  | .07 | 0.01 | **< .001** |  | .07 | 0.01 | **< .001** |
| Timepoint^2^ | -.02 | 0.00 | **.003** |  | -.02 | 0.00 | **< .001** |  | -.02 | 0.00 | **< .001** |
| Women (vs. men) | .07 | 0.03 | .054 |  | .07 | 0.03 | **.049** |  | .05 | 0.05 | .331 |
| Cohort B (vs. cohort A) | .17 | 0.03 | **< .001** |  | .18 | 0.03 | **< .001** |  | .21 | 0.04 | **< .001** |
| SG (vs. CG) | .10 | 0.03 | **.003** |  | .09 | 0.03 | **.004** |  | .07 | 0.07 | .271 |
| Timepoint x SG | .18 | 0.02 | **< .001** |  | .18 | 0.02 | **< .001** |  | .19 | 0.02 | **< .001** |
| Timepoint^2^ x SG | -.04 | 0.00 | **< .001** |  | -.05 | 0.00 | **< .001** |  | -.05 | 0.00 | **< .001** |
| PGS |  |  |  |  | .05 | 0.02 | **.034** |  | .10 | 0.04 | **.006** |
| PGS x SG |  |  |  |  | .02 | 0.03 | .460 |  | .03 | 0.03 | .307 |
| PGS x timepoint |  |  |  |  | .02 | 0.01 | .093 |  | .02 | 0.01 | .089 |
| PGS x timepoint^2^ |  |  |  |  | -.01 | 0.00 | **.005** |  | -.01 | 0.00 | **.005** |
| PGS x timepoint x SG |  |  |  |  | -.06 | 0.02 | **.001** |  | -.06 | 0.02 | **.001** |
| PGS x timepoint^2^ x SG |  |  |  |  | .02 | 0.00 | **< .001** |  | .02 | 0.00 | **< .001** |
| PC1 |  |  |  |  |  |  |  |  | -.14 | 0.46 | .759 |
| PC2 |  |  |  |  |  |  |  |  | -.44 | 0.49 | .371 |
| PC3 |  |  |  |  |  |  |  |  | .06 | 0.48 | .902 |
| PC4 |  |  |  |  |  |  |  |  | -.41 | 0.47 | .383 |
| PC5 |  |  |  |  |  |  |  |  | -.16 | 0.48 | .734 |
| SG x PC1 |  |  |  |  |  |  |  |  | -.49 | 0.66 | .457 |
| SG x PC2 |  |  |  |  |  |  |  |  | -.35 | 0.68 | .608 |
| SG x PC3 |  |  |  |  |  |  |  |  | -.01 | 0.67 | .989 |
| SG x PC4 |  |  |  |  |  |  |  |  | .18 | 0.68 | .787 |
| SG x PC5 |  |  |  |  |  |  |  |  | .26 | 0.67 | .702 |
| SG x Women |  |  |  |  |  |  |  |  | .07 | 0.07 | .330 |
| SG x Cohort B |  |  |  |  |  |  |  |  | -.06 | 0.06 | .349 |
| PGS x PC1 |  |  |  |  |  |  |  |  | -.05 | 0.32 | .880 |
| PGS x PC2 |  |  |  |  |  |  |  |  | .25 | 0.34 | .457 |
| PGS x PC3 |  |  |  |  |  |  |  |  | .18 | 0.35 | .605 |
| PGS x PC4 |  |  |  |  |  |  |  |  | -.15 | 0.35 | .671 |
| PGS x PC5 |  |  |  |  |  |  |  |  | .59 | 0.38 | .121 |
| PGS x Women |  |  |  |  |  |  |  |  | -.05 | 0.03 | .182 |
| PGS x Cohort B |  |  |  |  |  |  |  |  | -.04 | 0.03 | .200 |
| AIC | 69670.06 | | |  | 69643.29 | | |  | 69663.24 | | |
| BIC | 69751.59 | | |  | 69769.29 | | |  | 69930.06 | | |
| Log-likelihood | -34824.03 | | |  | -34804.65 | | |  | -34795.62 | | |
| Observations | 12230 | | |  | 12230 | | |  | 12230 | | |
| *n* | 432 | | |  | 432 | | |  | 432 | | |
| *SD*: participant (Intercept) | 0.31 | | |  | 0.31 | | |  | 0.30 | | |
| *SD*: timepoint | 0.11 | | |  | 0.11 | | |  | 0.11 | | |
| Correlation: participant x timepoint | -.27 | | |  | -.27 | | |  | -.29 | | |
| Marginal *R^2^* | 0.1006 | | |  | 0.1159 | | |  | 0.1519 | | |
| Conditional *R^2^* | 0.6612 | | |  | 0.6609 | | |  | 0.6668 | | |

Abbreviations: CG = Control group; PC = Principal component; PGS = Polygenic score; *SD* = Standard deviation; *SE* = Standard error; SG = Stress group.

^a^The group.model consists of a random intercept for participant, a random slope for the linear time trend and the fixed effects for the linear and quadratic time trend, group and its interactions with the time trends as well as the covariates sex and cohort.

^b^The PGS.model includes the additional fixed effects PGS, PGS in interaction with group as well as with the linear and quadratic time trend, and the three-way interaction of the PGS, group and the time trends.

^c^In the PC.model the covariates PC1 to 5 are added as well as the interactions PGS x covariates and group x covariates.


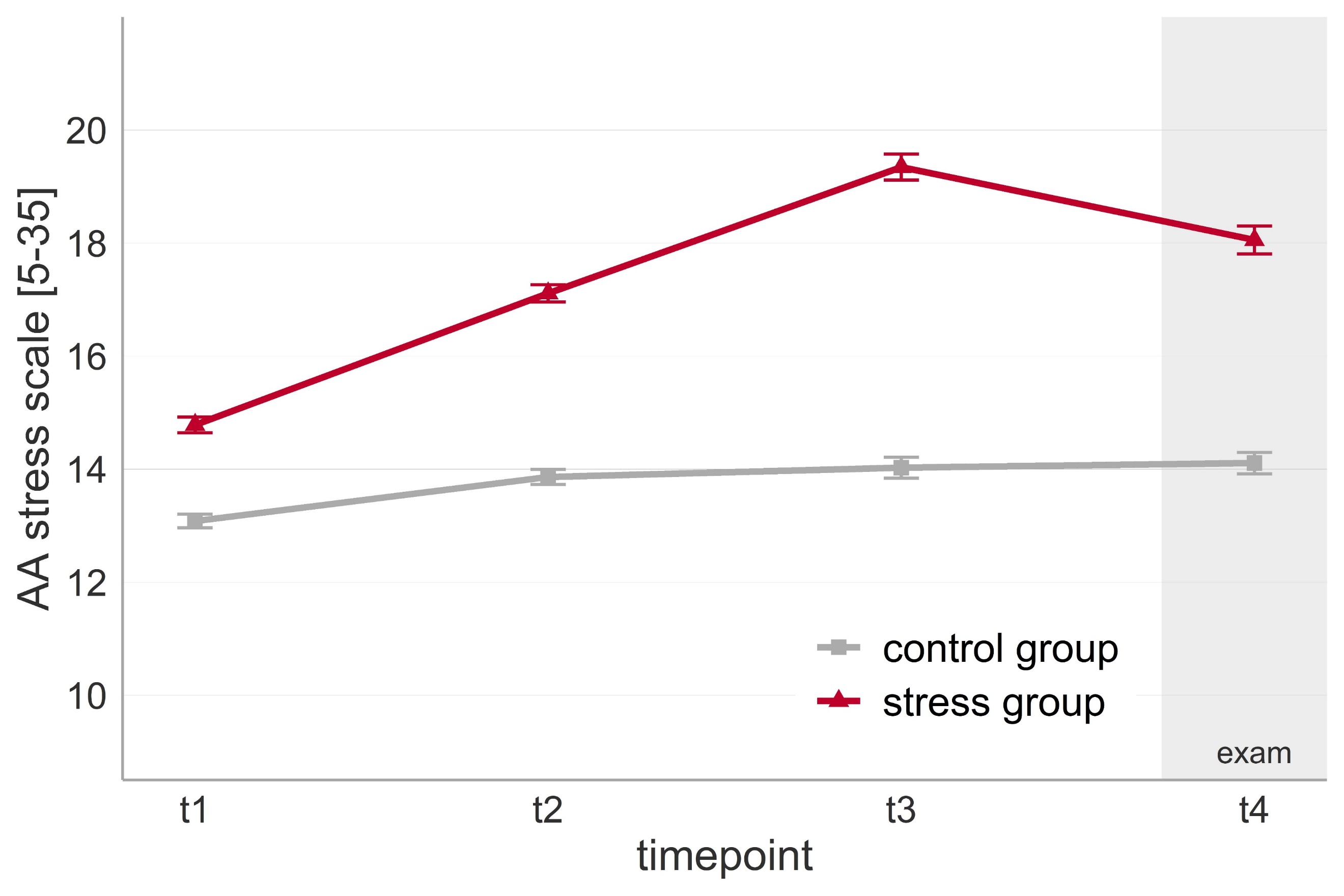


FIGURE S7. Time course of mean perceived stress levels (± SEM) in the stress group (SG) and control group (CG) until the exam at timepoint 4. Adapted from Giglberger et al.^1^

TABLE S9. Parameter estimates for overall effects of the models only containing the stress group (SG) with perceived stress as dependent variable and the polygenic score for neuroticism as predictor.

|  | **SG.model^a^** | | |  | **NEU-PGS.model^b^** | | |  | **PC.model^c^** | | |
| --- | --- | --- | --- | --- | --- | --- | --- | --- | --- | --- | --- |
|  | Estimates | *SE* | *p* |  | Estimates | *SE* | *p* |  | Estimates | *SE* | *p* |
| Intercept | 2.55 | 0.05 | **< .001** |  | 2.54 | 0.05 | **< .001** |  | 2.53 | 0.04 | **< .001** |
| Timepoint | .25 | 0.01 | **< .001** |  | .25 | 0.01 | **< .001** |  | .25 | 0.01 | **< .001** |
| Timepoint^2^ | -.06 | 0.00 | **< .001** |  | -.07 | 0.00 | **< .001** |  | -.07 | 0.00 | **< .001** |
| Women (vs. men) | .10 | 0.05 | **.034** |  | .10 | 0.05 | **.036** |  | .11 | 0.05 | **.014** |
| Cohort B (vs. cohort A) | .14 | 0.04 | **.001** |  | .15 | 0.04 | **< .001** |  | .17 | 0.04 | **< .001** |
| PGS |  |  |  |  | .07 | 0.02 | **.002** |  | .17 | 0.04 | **< .001** |
| PGS x timepoint |  |  |  |  | -.04 | 0.01 | **.004** |  | -.04 | 0.01 | **.006** |
| PGS x timepoint^2^ |  |  |  |  | .01 | 0.00 | **< .001** |  | .01 | 0.00 | **< .001** |
| PC1 |  |  |  |  |  |  |  |  | -.62 | 0.47 | .191 |
| PC2 |  |  |  |  |  |  |  |  | -.62 | 0.46 | .177 |
| PC3 |  |  |  |  |  |  |  |  | .12 | 0.46 | .791 |
| PC4 |  |  |  |  |  |  |  |  | -.21 | 0.48 | .659 |
| PC5 |  |  |  |  |  |  |  |  | .00 | 0.45 | .993 |
| PGS x PC1 |  |  |  |  |  |  |  |  | -.30 | 0.41 | .464 |
| PGS x PC2 |  |  |  |  |  |  |  |  | 1.15 | 0.47 | **.013** |
| PGS x PC3 |  |  |  |  |  |  |  |  | .80 | 0.50 | .108 |
| PGS x PC4 |  |  |  |  |  |  |  |  | -.29 | 0.49 | .556 |
| PGS x PC5 |  |  |  |  |  |  |  |  | 1.14 | 0.51 | **.027** |
| PGS x Women |  |  |  |  |  |  |  |  | -.06 | 0.05 | .210 |
| PGS x Cohort B |  |  |  |  |  |  |  |  | -.09 | 0.04 | .055 |
| AIC | 33850.42 | | |  | 33832.07 | | |  | 33831.72 | | |
| BIC | 33903.71 | | |  | 33905.35 | | |  | 33984.94 | | |
| Log-likelihood | -16917.21 | | |  | -16905.03 | | |  | -16892.86 | | |
| Observations | 5778 | | |  | 5778 | | |  | 5778 | | |
| *n* | 218 | | |  | 218 | | |  | 218 | | |
| *SD*: participant (Intercept) | 0.31 | | |  | 0.31 | | |  | 0.29 | | |
| *SD*: timepoint | 0.12 | | |  | 0.12 | | |  | 0.12 | | |
| Correlation: participant x timepoint | -.28 | | |  | -.29 | | |  | -.31 | | |
| Marginal *R^2^* | 0.0845 | | |  | 0.1031 | | |  | 0.1747 | | |
| Conditional *R^2^* | 0.6824 | | |  | 0.6821 | | |  | 0.6822 | | |

Abbreviations: PC = Principal component; PGS = Polygenic score; *SD* = Standard deviation; *SE* = Standard error; SG = Stress group.

^a^The group.model consists of a random intercept for participant, a random slope for the linear time trend and the fixed effects for the linear and quadratic time trend and the covariates sex and cohort.

^b^The PGS.model includes the additional fixed effects PGS, PGS in interaction with the linear and quadratic time trend.

^c^In the PC.model the covariates PC1 to 5 are added as well as the interactions PGS x covariates.

TABLE S10. Parameter estimates for overall effects of the models only containing the control group (CG) with perceived stress as dependent variable and the polygenic score for neuroticism as predictor.

|  | **CG.model^a^** | | |  | **NEU-PGS.model^b^** | | |  | **PC.model^c^** | | |
| --- | --- | --- | --- | --- | --- | --- | --- | --- | --- | --- | --- |
|  | Estimates | *SE* | *p* |  | Estimates | *SE* | *p* |  | Estimates | *SE* | *p* |
| Intercept | 2.49 | 0.05 | **< .001** |  | 2.49 | 0.05 | **< .001** |  | 2.48 | 0.05 | **< .001** |
| Timepoint | .01 | 0.01 | .225 |  | .01 | 0.01 | .223 |  | .01 | 0.01 | .222 |
| Women (vs. men) | .03 | 0.05 | .535 |  | .04 | 0.05 | .468 |  | .04 | 0.05 | .412 |
| Cohort B (vs. cohort A) | .20 | 0.04 | **< .001** |  | .21 | 0.04 | **< .001** |  | .21 | 0.04 | **< .001** |
| PGS |  |  |  |  | .06 | 0.02 | **.010** |  | .11 | 0.05 | **.036** |
| PGS x timepoint |  |  |  |  | -.01 | 0.01 | .543 |  | -.01 | 0.01 | .540 |
| PC1 |  |  |  |  |  |  |  |  | -.21 | 0.48 | .662 |
| PC2 |  |  |  |  |  |  |  |  | -.40 | 0.50 | .422 |
| PC3 |  |  |  |  |  |  |  |  | -.02 | 0.48 | .966 |
| PC4 |  |  |  |  |  |  |  |  | -.35 | 0.48 | .468 |
| PC5 |  |  |  |  |  |  |  |  | -.29 | 0.49 | .556 |
| PGS x PC1 |  |  |  |  |  |  |  |  | -.01 | 0.53 | .978 |
| PGS x PC2 |  |  |  |  |  |  |  |  | -.59 | 0.51 | .242 |
| PGS x PC3 |  |  |  |  |  |  |  |  | -.16 | 0.50 | .750 |
| PGS x PC4 |  |  |  |  |  |  |  |  | .23 | 0.53 | .667 |
| PGS x PC5 |  |  |  |  |  |  |  |  | .44 | 0.59 | .463 |
| PGS x Women |  |  |  |  |  |  |  |  | -.05 | 0.05 | .292 |
| PGS x Cohort B |  |  |  |  |  |  |  |  | -.01 | 0.04 | .759 |
| AIC | 35861.50 | | |  | 35858.96 | | |  | 35878.21 | | |
| BIC | 35908.91 | | |  | 35919.91 | | |  | 36020.42 | | |
| Log-likelihood | -17923.75 | | |  | -17920.48 | | |  | -17918.10 | | |
| Observations | 6452 | | |  | 6452 | | |  | 6452 | | |
| *n* | 214 | | |  | 214 | | |  | 214 | | |
| *SD*: participant (Intercept) | 0.31 | | |  | 0.30 | | |  | 0.30 | | |
| *SD*: timepoint | 0.11 | | |  | 0.11 | | |  | 0.11 | | |
| Correlation: participant x timepoint | -.26 | | |  | -.26 | | |  | -.27 | | |
| Marginal *R^2^* | 0.0296 | | |  | 0.0444 | | |  | 0.0555 | | |
| Conditional *R^2^* | 0.6147 | | |  | 0.6151 | | |  | 0.6067 | | |

Abbreviations: CG = Control group; PC = Principal component; PGS = Polygenic score; *SD* = Standard deviation; *SE* = Standard error.

^a^The group.model consists of a random intercept for participant, a random slope for the linear time trend and the fixed effects for the linear time trend and the covariates sex and cohort.

^b^The PGS.model includes the additional fixed effects PGS, PGS in interaction with the linear time trend.

^c^In the PC.model the covariates PC1 to 5 are added as well as the interactions PGS x covariates.

TABLE S11. Parameter estimates for overall effects of the models with perceived stress as dependent variable and the polygenic score continuous shrinkage (PGScs) for neuroticism as predictor.

|  | **group.model^a^** | | |  | **NEU-PGScs.model^b^** | | |  | **PC.model^c^** | | |
| --- | --- | --- | --- | --- | --- | --- | --- | --- | --- | --- | --- |
|  | Estimates | *SE* | *p* |  | Estimates | *SE* | *p* |  | Estimates | *SE* | *p* |
| Intercept | 2.46 | 0.04 | **< .001** |  | 2.45 | 0.04 | **< .001** |  | 2.43 | 0.05 | **< .001** |
| Timepoint | .07 | 0.01 | **< .001** |  | .07 | 0.01 | **< .001** |  | .07 | 0.01 | **< .001** |
| Timepoint^2^ | -.02 | 0.00 | **.003** |  | -.02 | 0.00 | **< .001** |  | -.02 | 0.00 | **< .001** |
| Women (vs. men) | .07 | 0.03 | .054 |  | .07 | 0.03 | **.038** |  | .07 | 0.05 | .172 |
| Cohort B (vs. cohort A) | .17 | 0.03 | **< .001** |  | .18 | 0.03 | **< .001** |  | .21 | 0.04 | **< .001** |
| SG (vs. CG) | .10 | 0.03 | **.003** |  | .09 | 0.03 | **.003** |  | .10 | 0.07 | .146 |
| Timepoint x SG | .18 | 0.02 | **< .001** |  | .18 | 0.02 | **< .001** |  | .18 | 0.02 | **< .001** |
| Timepoint^2^ x SG | -.04 | 0.00 | **< .001** |  | -.04 | 0.00 | **< .001** |  | -.04 | 0.00 | **< .001** |
| PGScs |  |  |  |  | .06 | 0.02 | **.011** |  | .12 | 0.04 | **.001** |
| PGScs x SG |  |  |  |  | .02 | 0.03 | .550 |  | .03 | 0.03 | .342 |
| PGScs x timepoint |  |  |  |  | -.02 | 0.01 | .069 |  | -.02 | 0.01 | .073 |
| PGScs x timepoint^2^ |  |  |  |  | .01 | 0.00 | .075 |  | .01 | 0.00 | .077 |
| PGScs x timepoint x SG |  |  |  |  | -.03 | 0.02 | .081 |  | -.03 | 0.02 | .076 |
| PGScs x timepoint^2^ x SG |  |  |  |  | .02 | 0.00 | **< .001** |  | .02 | 0.00 | **< .001** |
| PC1 |  |  |  |  |  |  |  |  | -.12 | 0.46 | .792 |
| PC2 |  |  |  |  |  |  |  |  | -.36 | 0.49 | .463 |
| PC3 |  |  |  |  |  |  |  |  | -.06 | 0.47 | .901 |
| PC4 |  |  |  |  |  |  |  |  | -.43 | 0.47 | .369 |
| PC5 |  |  |  |  |  |  |  |  | -.22 | 0.48 | .650 |
| SG x PC1 |  |  |  |  |  |  |  |  | -.66 | 0.65 | .316 |
| SG x PC2 |  |  |  |  |  |  |  |  | -.56 | 0.68 | .411 |
| SG x PC3 |  |  |  |  |  |  |  |  | .13 | 0.67 | .850 |
| SG x PC4 |  |  |  |  |  |  |  |  | .26 | 0.68 | .701 |
| SG x PC5 |  |  |  |  |  |  |  |  | .34 | 0.67 | .609 |
| SG x Women |  |  |  |  |  |  |  |  | .04 | 0.07 | .543 |
| SG x Cohort B |  |  |  |  |  |  |  |  | -.06 | 0.06 | .272 |
| PGScs x PC1 |  |  |  |  |  |  |  |  | .36 | 0.33 | .272 |
| PGScs x PC2 |  |  |  |  |  |  |  |  | .07 | 0.35 | .829 |
| PGScs x PC3 |  |  |  |  |  |  |  |  | .20 | 0.33 | .554 |
| PGScs x PC4 |  |  |  |  |  |  |  |  | .42 | 0.36 | .242 |
| PGScs x PC5 |  |  |  |  |  |  |  |  | .68 | 0.36 | .063 |
| PGScs x Women |  |  |  |  |  |  |  |  | -.08 | 0.03 | **.017** |
| PGScs x Cohort B |  |  |  |  |  |  |  |  | -.01 | 0.03 | .758 |
| AIC | 69670.06 | | |  | 69615.73 | | |  | 69632.63 | | |
| BIC | 69751.59 | | |  | 69741.73 | | |  | 69899.45 | | |
| Log-likelihood | -34824.03 | | |  | -34790.87 | | |  | -34780.32 | | |
| Observations | 12230 | | |  | 12230 | | |  | 12230 | | |
| *n* | 432 | | |  | 432 | | |  | 432 | | |
| *SD*: participant (Intercept) | 0.31 | | |  | 0.31 | | |  | 0.30 | | |
| *SD*: timepoint | 0.11 | | |  | 0.11 | | |  | 0.11 | | |
| Correlation: participant x timepoint | -.27 | | |  | -.28 | | |  | -.30 | | |
| Marginal *R^2^* | 0.1006 | | |  | 0.1182 | | |  | 0.1481 | | |
| Conditional *R^2^* | 0.6612 | | |  | 0.6619 | | |  | 0.6641 | | |

Abbreviations: CG = Control group; PC = Principal component; PGScs = Polygenic score continuous shrinkage; *SD* = Standard deviation; *SE* = Standard error; SG = Stress group.

^a^The group.model consists of a random intercept for participant, a random slope for the linear time trend and the fixed effects for the linear and quadratic time trend, group and its interactions with the time trends as well as the covariates sex and cohort.

^b^The PGS.model includes the additional fixed effects PGScs, PGScs in interaction with group as well as with the linear and quadratic time trend, and the three-way interaction of the PGScs, group and the time trends.

^c^In the PC.model the covariates PC1 to 5 are added as well as the interactions PGScs x covariates and group x covariates.

TABLE S12. Parameter estimates for overall effects of the models only containing the stress group (SG) with perceived stress as dependent variable and the polygenic score continuous shrinkage (PGScs) for neuroticism as predictor.

|  | **SG.model^a^** | | |  | **NEU-PGScs.model^b^** | | |  | **PC.model^c^** | | |
| --- | --- | --- | --- | --- | --- | --- | --- | --- | --- | --- | --- |
|  | Estimates | *SE* | *p* |  | Estimates | *SE* | *p* |  | Estimates | *SE* | *p* |
| Intercept | 2.55 | 0.05 | **< .001** |  | 2.54 | 0.05 | **< .001** |  | **2.54** | **0.04** | **< .001** |
| Timepoint | .25 | 0.01 | **< .001** |  | .26 | 0.01 | **< .001** |  | **.26** | **0.01** | **< .001** |
| Timepoint^2^ | -.06 | 0.00 | **< .001** |  | -.07 | 0.00 | **< .001** |  | **-.07** | **0.00** | **< .001** |
| Women (vs. men) | .10 | 0.05 | **.034** |  | .10 | 0.05 | **.033** |  | **.10** | **0.05** | **.034** |
| Cohort B (vs. cohort A) | .14 | 0.04 | **.001** |  | .15 | 0.04 | **< .001** |  | **.14** | **0.04** | **.001** |
| PGS |  |  |  |  | .07 | 0.02 | **.001** |  | **.20** | **0.05** | **< .001** |
| PGScs x timepoint |  |  |  |  | -.06 | 0.01 | **< .001** |  | **-.06** | **0.01** | **< .001** |
| PGScs x timepoint^2^ |  |  |  |  | .02 | 0.00 | **< .001** |  | **.02** | **0.00** | **< .001** |
| PC1 |  |  |  |  |  |  |  |  | -.90 | 0.46 | .054 |
| PC2 |  |  |  |  |  |  |  |  | -.74 | 0.47 | .112 |
| PC3 |  |  |  |  |  |  |  |  | .17 | 0.47 | .724 |
| PC4 |  |  |  |  |  |  |  |  | -.22 | 0.48 | .646 |
| PC5 |  |  |  |  |  |  |  |  | .07 | 0.45 | .876 |
| PGScs x PC1 |  |  |  |  |  |  |  |  | -.16 | 0.40 | .691 |
| PGScs x PC2 |  |  |  |  |  |  |  |  | .74 | 0.47 | .117 |
| PGScs x PC3 |  |  |  |  |  |  |  |  | .80 | 0.47 | .089 |
| PGScs x PC4 |  |  |  |  |  |  |  |  | .05 | 0.54 | .926 |
| PGScs x PC5 |  |  |  |  |  |  |  |  | 1.23 | 0.48 | **.010** |
| PGScs x Women |  |  |  |  |  |  |  |  | -.09 | 0.05 | .070 |
| PGScs x Cohort B |  |  |  |  |  |  |  |  | -.09 | 0.04 | **.034** |
| AIC | 33850.42 | | |  | 33798.73 | | |  | 33800.60 | | |
| BIC | 33903.71 | | |  | 33872.01 | | |  | 33953.82 | | |
| Log-likelihood | -16917.21 | | |  | -16888.37 | | |  | -16877.30 | | |
| Observations | 5778 | | |  | 5778 | | |  | 5778 | | |
| *n* | 218 | | |  | 218 | | |  | 218 | | |
| *SD*: participant (Intercept) | 0.31 | | |  | 0.31 | | |  | 0.29 | | |
| *SD*: timepoint | 0.12 | | |  | 0.12 | | |  | 0.12 | | |
| Correlation: participant x timepoint | -.28 | | |  | -.31 | | |  | -.33 | | |
| Marginal *R^2^* | 0.0845 | | |  | 0.1127 | | |  | 0.1781 | | |
| Conditional *R^2^* | 0.6824 | | |  | 0.6783 | | |  | 0.6838 | | |

Abbreviations: PC = Principal component; PGScs = Polygenic score continuous shrinkage; *SD* = Standard deviation; *SE* = Standard error; SG = Stress group.

^a^The group.model consists of a random intercept for participant, a random slope for the linear time trend and the fixed effects for the linear and quadratic time trend and the covariates sex and cohort.

^b^The PGS.model includes the additional fixed effects PGS, PGScs in interaction with the linear and quadratic time trend.

^c^In the PC.model the covariates PC1 to 5 are added as well as the interactions PGScs x covariates.

TABLE S13. Parameter estimates for overall effects of the models only containing the control group (CG) with perceived stress as dependent variable and the polygenic score continuous shrinkage (PGScs) for neuroticism as predictor.

|  | **CG.model^a^** | | |  | **NEU-PGScs.model^b^** | | |  | **PC.model^c^** | | |
| --- | --- | --- | --- | --- | --- | --- | --- | --- | --- | --- | --- |
|  | Estimates | *SE* | *p* |  | Estimates | *SE* | *p* |  | Estimates | *SE* | *p* |
| Intercept | 2.49 | 0.05 | **< .001** |  | 2.48 | 0.05 | **< .001** |  | 2.45 | 0.05 | **< .001** |
| Timepoint | .01 | 0.01 | .225 |  | .01 | 0.01 | .218 |  | .01 | 0.01 | .217 |
| Women (vs. men) | .03 | 0.05 | .535 |  | .04 | 0.05 | .388 |  | .07 | 0.05 | .180 |
| Cohort B (vs. cohort A) | .20 | 0.04 | **< .001** |  | .21 | 0.04 | **< .001** |  | .21 | 0.04 | **< .001** |
| PGS |  |  |  |  | .05 | 0.02 | **.021** |  | .10 | 0.05 | **.049** |
| PGScs x timepoint |  |  |  |  | -.01 | 0.01 | .460 |  | -.01 | 0.01 | .429 |
| PC1 |  |  |  |  |  |  |  |  | -.14 | 0.46 | .769 |
| PC2 |  |  |  |  |  |  |  |  | -.22 | 0.49 | .654 |
| PC3 |  |  |  |  |  |  |  |  | -.17 | 0.47 | .725 |
| PC4 |  |  |  |  |  |  |  |  | -.39 | 0.47 | .408 |
| PC5 |  |  |  |  |  |  |  |  | -.25 | 0.48 | .604 |
| PGScs x PC1 |  |  |  |  |  |  |  |  | .76 | 0.55 | .164 |
| PGScs x PC2 |  |  |  |  |  |  |  |  | -.52 | 0.52 | .313 |
| PGScs x PC3 |  |  |  |  |  |  |  |  | -.21 | 0.48 | .666 |
| PGScs x PC4 |  |  |  |  |  |  |  |  | .65 | 0.48 | .177 |
| PGScs x PC5 |  |  |  |  |  |  |  |  | .56 | 0.61 | .362 |
| PGScs x Women |  |  |  |  |  |  |  |  | -.09 | 0.05 | .059 |
| PGScs x Cohort B |  |  |  |  |  |  |  |  | .05 | 0.05 | .283 |
| AIC | 35861.50 | | |  | 35860.18 | | |  | 35871.21 | | |
| BIC | 35908.91 | | |  | 35921.13 | | |  | 36013.43 | | |
| Log-likelihood | -17923.75 | | |  | -17921.09 | | |  | -17914.61 | | |
| Observations | 6452 | | |  | 6452 | | |  | 6452 | | |
| *n* | 214 | | |  | 214 | | |  | 214 | | |
| *SD*: participant (Intercept) | 0.31 | | |  | 0.30 | | |  | 0.30 | | |
| *SD*: timepoint | 0.11 | | |  | 0.11 | | |  | 0.11 | | |
| Correlation: participant x timepoint | -.26 | | |  | -.26 | | |  | -.28 | | |
| Marginal *R^2^* | 0.0296 | | |  | 0.0400 | | |  | 0.0602 | | |
| Conditional *R^2^* | 0.6147 | | |  | 0.6070 | | |  | 0.6023 | | |

Abbreviations: CG = Control group; PC = Principal component; PGScs = Polygenic score continuous shrinkage; *SD* = Standard deviation; *SE* = Standard error.

^a^The group.model consists of a random intercept for participant, a random slope for the linear time trend and the fixed effects for the linear time trend and the covariates sex and cohort.

^b^The PGS.model includes the additional fixed effects PGS, PGScs in interaction with the linear time trend.

^c^In the PC.model the covariates PC1 to 5 are added as well as the interactions PGScs x covariates.

# Depression symptoms

TABLE S14. Parameter estimates for overall effects of the models with depression symptoms as dependent variable and the polygenic score for depression as predictor.

|  | **group.model^a^** | | |  | **DEP-PGS.model^b^** | | |  | **PC.model^c^** | | |
| --- | --- | --- | --- | --- | --- | --- | --- | --- | --- | --- | --- |
|  | Estimates | *SE* | *p* |  | Estimates | *SE* | *p* |  | Estimates | *SE* | *p* |
| Intercept | 1.31 | 0.08 | **< .001** |  | 1.31 | 0.08 | **< .001** |  | 1.37 | 0.10 | **< .001** |
| Timepoint | .17 | 0.09 | .059 |  | .17 | 0.09 | .058 |  | .17 | 0.09 | .061 |
| Timepoint^2^ | -.06 | 0.04 | .123 |  | -.06 | 0.04 | .122 |  | -.06 | 0.04 | .121 |
| Women (vs. men) | -.14 | 0.07 | .**043** |  | -.14 | 0.07 | **.045** |  | -.21 | 0.10 | **.044** |
| SG (vs. CG) | -.08 | 0.08 | .285 |  | -.08 | 0.08 | .282 |  | -.17 | 0.13 | .209 |
| Timepoint x SG | .50 | 0.12 | **< .001** |  | .50 | 0.12 | **< .001** |  | .50 | 0.12 | **< .001** |
| Timepoint^2^ x SG | -.11 | 0.06 | **.043** |  | -.11 | 0.06 | **.047** |  | -.11 | 0.06 | **.048** |
| PGS |  |  |  |  | .07 | 0.06 | .241 |  | .06 | 0.08 | .429 |
| PGS x SG |  |  |  |  | -.05 | 0.08 | .520 |  | -.05 | 0.08 | .557 |
| PGS x timepoint |  |  |  |  | -.06 | 0.09 | .527 |  | -.06 | 0.09 | .527 |
| PGS x timepoint^2^ |  |  |  |  | .01 | 0.04 | .865 |  | .01 | 0.04 | .863 |
| PGS x timepoint x SG |  |  |  |  | .20 | 0.12 | .105 |  | .20 | 0.12 | .108 |
| PGS x timepoint^2^ x SG |  |  |  |  | -.07 | 0.06 | .198 |  | -.07 | 0.06 | .202 |
| PC1 |  |  |  |  |  |  |  |  | .24 | 0.96 | .799 |
| PC2 |  |  |  |  |  |  |  |  | .23 | 1.07 | .833 |
| PC3 |  |  |  |  |  |  |  |  | .54 | 0.99 | .587 |
| PC4 |  |  |  |  |  |  |  |  | .09 | 1.03 | .927 |
| PC5 |  |  |  |  |  |  |  |  | -.26 | 1.02 | .796 |
| SG x PC1 |  |  |  |  |  |  |  |  | -.47 | 1.36 | .731 |
| SG x PC2 |  |  |  |  |  |  |  |  | -.92 | 1.45 | .523 |
| SG x PC3 |  |  |  |  |  |  |  |  | .40 | 1.39 | .776 |
| SG x PC4 |  |  |  |  |  |  |  |  | 1.38 | 1.43 | .335 |
| SG x PC5 |  |  |  |  |  |  |  |  | 1.38 | 1.41 | .330 |
| SG x Women |  |  |  |  |  |  |  |  | .12 | 0.14 | .420 |
| PGS x PC1 |  |  |  |  |  |  |  |  | -.68 | 0.68 | .320 |
| PGS x PC2 |  |  |  |  |  |  |  |  | -.50 | 0.73 | .491 |
| PGS x PC3 |  |  |  |  |  |  |  |  | .43 | 0.68 | .530 |
| PGS x PC4 |  |  |  |  |  |  |  |  | -.41 | 0.67 | .536 |
| PGS x PC5 |  |  |  |  |  |  |  |  | .46 | 0.71 | .521 |
| PGS x Women |  |  |  |  |  |  |  |  | .00 | 0.07 | .984 |
| AIC | 5835.56 | | |  | 5840.48 | | |  | 5864.73 | | |
| BIC | 5886.71 | | |  | 5922.33 | | |  | 6033.55 | | |
| Log-likelihood | -2907.78 | | |  | -2904.24 | | |  | -2899.37 | | |
| Observations | 1231 | | |  | 1231 | | |  | 1231 | | |
| *n* | 432 | | |  | 432 | | |  | 432 | | |
| *SD*: participant (Intercept) | 0.58 | | |  | 0.58 | | |  | 0.57 | | |
| *SD*: timepoint | 0.18 | | |  | 0.18 | | |  | 0.18 | | |
| Correlation: participant x timepoint | -.31 | | |  | -.30 | | |  | -.28 | | |
| Marginal *R^2^* | 0.0848 | | |  | 0.0878 | | |  | 0.1015 | | |
| Conditional *R^2^* | 0.6076 | | |  | 0.6085 | | |  | 0.6087 | | |

Abbreviations: CG = Control group; PC = Principal component; PGS = Polygenic score; *SD* = Standard deviation; *SE* = Standard error; SG = Stress group.

^a^The group.model consists of a random intercept for participant, a random slope for the linear time trend and the fixed effects for the linear and quadratic time trend, group and its interactions with the time trends as well as the covariates sex and cohort.

^b^The PGS.model includes the additional fixed effects PGS, PGS in interaction with group as well as with the linear and quadratic time trend, and the three-way interaction of the PGS, group and the time trends.

^c^In the PC.model the covariates PC1 to 5 are added as well as the interactions PGS x covariates and group x covariates.

TABLE S15. Parameter estimates for overall effects of the models with depression symptoms as dependent variable and the polygenic score for neuroticism as predictor.

|  | **group.model^a^** | | |  | **NEU-PGS.model^b^** | | |  | **PC.model^c^** | | |
| --- | --- | --- | --- | --- | --- | --- | --- | --- | --- | --- | --- |
|  | Estimates | *SE* | *p* |  | Estimates | *SE* | *p* |  | Estimates | *SE* | *p* |
| Intercept | 1.31 | 0.08 | **< .001** |  | 1.32 | 0.08 | **< .001** |  | 1.38 | 0.10 | **< .001** |
| Timepoint | .17 | 0.09 | .059 |  | .17 | 0.09 | .057 |  | .17 | 0.09 | .058 |
| Timepoint^2^ | -.06 | 0.04 | .123 |  | -.06 | 0.04 | .121 |  | -.06 | 0.04 | .122 |
| Women (vs. men) | -.14 | 0.07 | .**043** |  | -.15 | 0.07 | **.037** |  | -.22 | 0.10 | **.030** |
| SG (vs. CG) | -.08 | 0.08 | .285 |  | -.08 | 0.08 | .273 |  | -.18 | 0.13 | .160 |
| Timepoint x SG | .50 | 0.12 | **< .001** |  | .50 | 0.12 | **< .001** |  | .50 | 0.12 | **< .001** |
| Timepoint^2^ x SG | -.11 | 0.06 | **.043** |  | -.11 | 0.06 | **.046** |  | -.11 | 0.06 | **.047** |
| PGS |  |  |  |  | -.03 | 0.05 | .527 |  | -.03 | 0.07 | .722 |
| PGS x SG |  |  |  |  | .13 | 0.08 | .089 |  | .13 | 0.08 | .094 |
| PGS x timepoint |  |  |  |  | .09 | 0.09 | .351 |  | .08 | 0.09 | .358 |
| PGS x timepoint^2^ |  |  |  |  | -.05 | 0.04 | .297 |  | -.04 | 0.04 | .302 |
| PGS x timepoint x SG |  |  |  |  | -.04 | 0.13 | .762 |  | -.04 | 0.13 | .782 |
| PGS x timepoint^2^ x SG |  |  |  |  | .03 | 0.06 | .638 |  | .03 | 0.06 | .653 |
| PC1 |  |  |  |  |  |  |  |  | .19 | 0.96 | .847 |
| PC2 |  |  |  |  |  |  |  |  | .09 | 1.04 | .932 |
| PC3 |  |  |  |  |  |  |  |  | .59 | 0.98 | .548 |
| PC4 |  |  |  |  |  |  |  |  | .25 | 1.00 | .800 |
| PC5 |  |  |  |  |  |  |  |  | -.35 | 1.01 | .733 |
| SG x PC1 |  |  |  |  |  |  |  |  | -.81 | 1.36 | .551 |
| SG x PC2 |  |  |  |  |  |  |  |  | -.92 | 1.43 | .521 |
| SG x PC3 |  |  |  |  |  |  |  |  | .41 | 1.38 | .765 |
| SG x PC4 |  |  |  |  |  |  |  |  | 1.06 | 1.42 | .456 |
| SG x PC5 |  |  |  |  |  |  |  |  | 1.42 | 1.41 | .314 |
| SG x Women |  |  |  |  |  |  |  |  | .13 | 0.14 | .359 |
| PGS x PC1 |  |  |  |  |  |  |  |  | -.69 | 0.66 | .294 |
| PGS x PC2 |  |  |  |  |  |  |  |  | .44 | 0.70 | .529 |
| PGS x PC3 |  |  |  |  |  |  |  |  | .03 | 0.72 | .965 |
| PGS x PC4 |  |  |  |  |  |  |  |  | .11 | 0.74 | .881 |
| PGS x PC5 |  |  |  |  |  |  |  |  | .57 | 0.79 | .472 |
| PGS x Women |  |  |  |  |  |  |  |  | -.01 | 0.07 | .868 |
| AIC | 5835.56 | | |  | 5838.66 | | |  | 5863.60 | | |
| BIC | 5886.71 | | |  | 5920.51 | | |  | 6032.42 | | |
| Log-likelihood | -2907.78 | | |  | -2903.33 | | |  | -2898.80 | | |
| Observations | 1231 | | |  | 1231 | | |  | 1231 | | |
| *n* | 432 | | |  | 432 | | |  | 432 | | |
| *SD*: participant (Intercept) | 0.58 | | |  | 0.58 | | |  | 0.57 | | |
| *SD*: timepoint | 0.18 | | |  | 0.18 | | |  | 0.18 | | |
| Correlation: participant x timepoint | -.31 | | |  | -.31 | | |  | -.32 | | |
| Marginal *R^2^* | 0.0848 | | |  | 0.0946 | | |  | 0.1072 | | |
| Conditional *R^2^* | 0.6076 | | |  | 0.6075 | | |  | 0.6071 | | |

Abbreviations: CG = Control group; PC = Principal component; PGS = Polygenic score; *SD* = Standard deviation; *SE* = Standard error; SG = Stress group.

^a^The group.model consists of a random intercept for participant, a random slope for the linear time trend and the fixed effects for the linear and quadratic time trend, group and its interactions with the time trends as well as the covariates sex and cohort.

^b^The PGS.model includes the additional fixed effects PGS, PGS in interaction with group as well as with the linear and quadratic time trend, and the three-way interaction of the PGS, group and the time trends.

^c^In the PC.model the covariates PC1 to 5 are added as well as the interactions PGS x covariates and group x covariates.


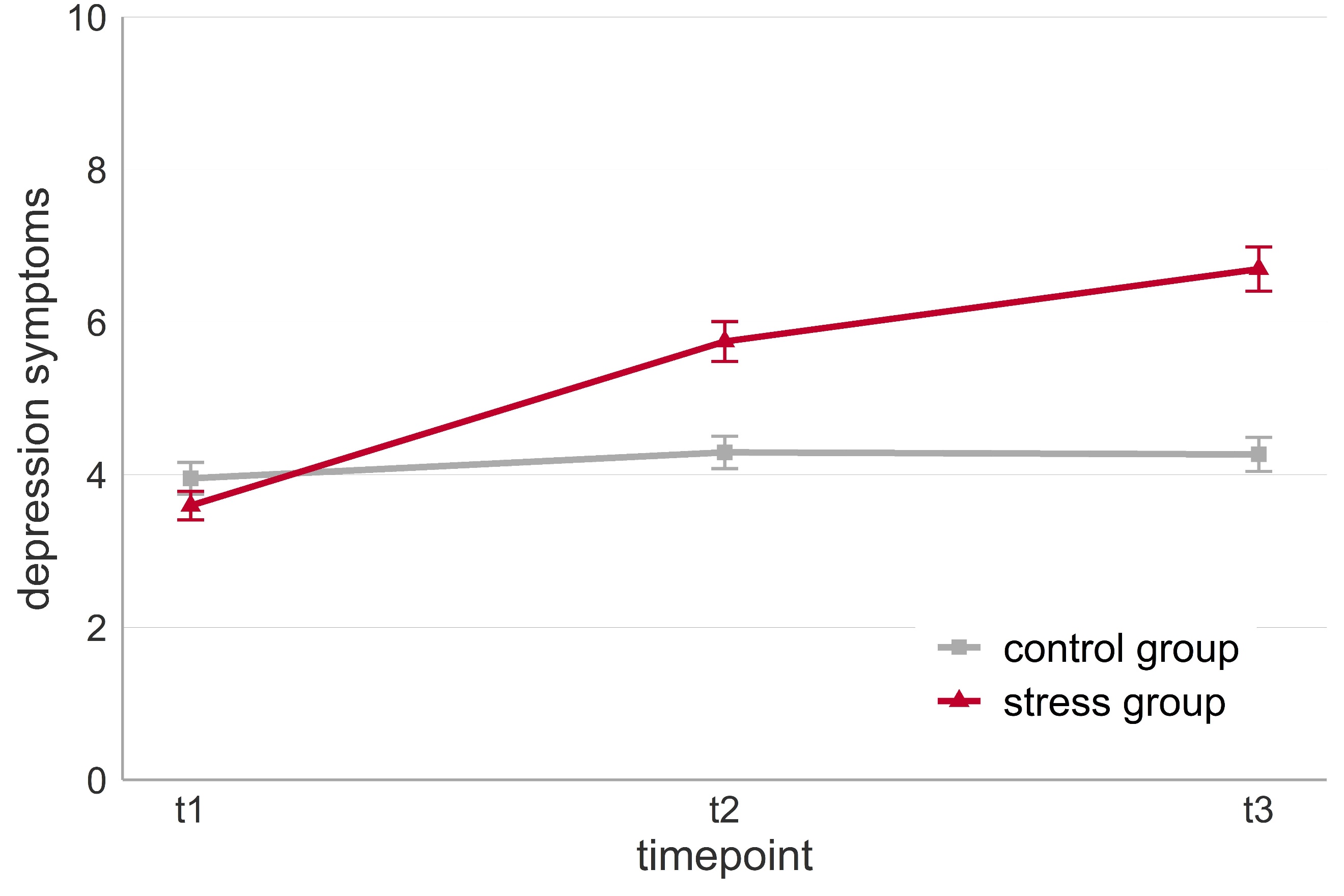


FIGURE S8. Time course of mean depression symptoms (± SEM) in the stress group (SG) and control group (CG) until one week prior exam. Adapted from Giglberger et al. ^1^


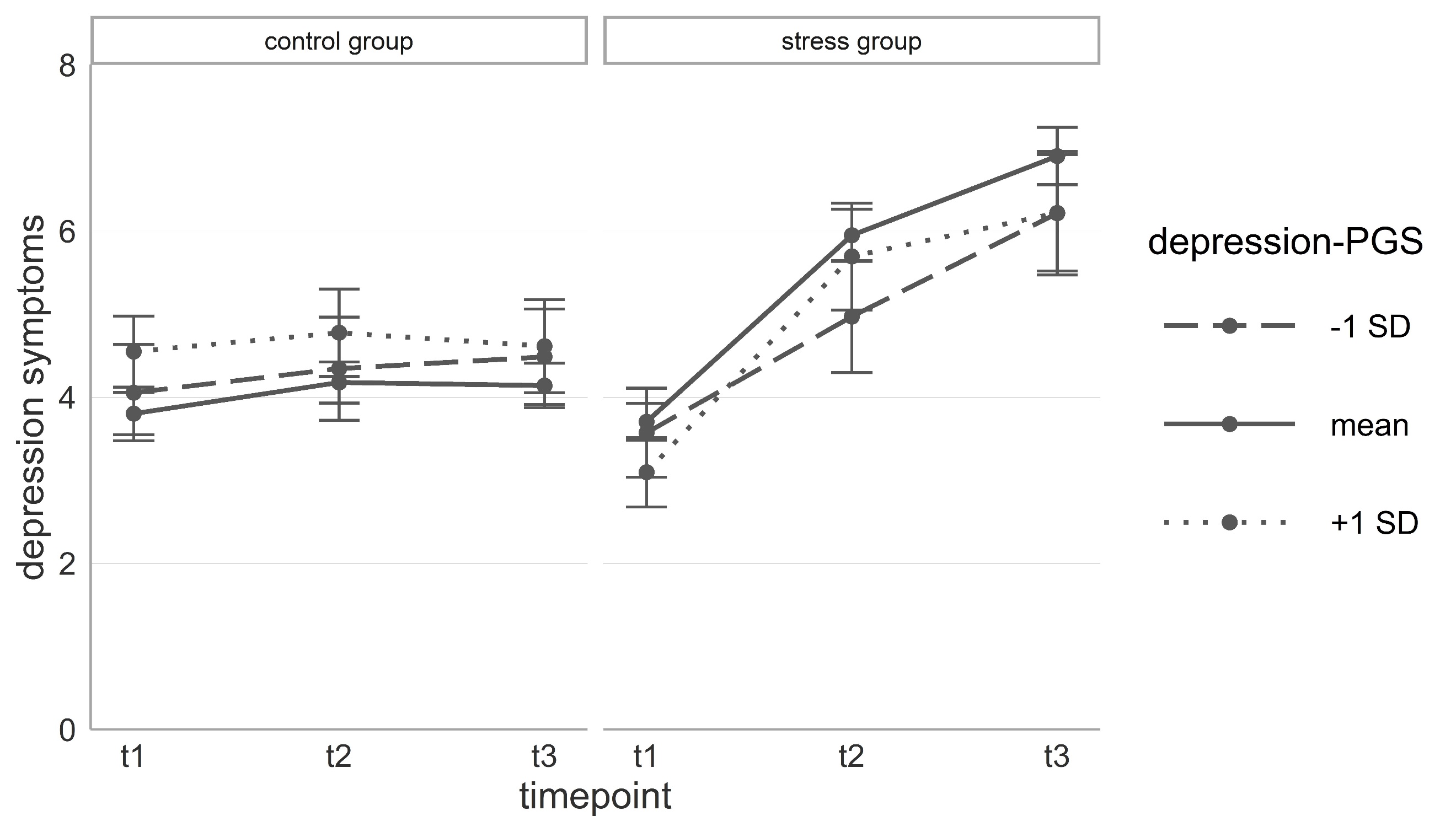


FIGURE S9. Time course of mean depression symptoms (± SEM) in the stress group (SG) and control group (CG) separated by polygenic score (PGS) for depression (grouping based on SD for illustrative purposes only). *SD* = standard deviation.


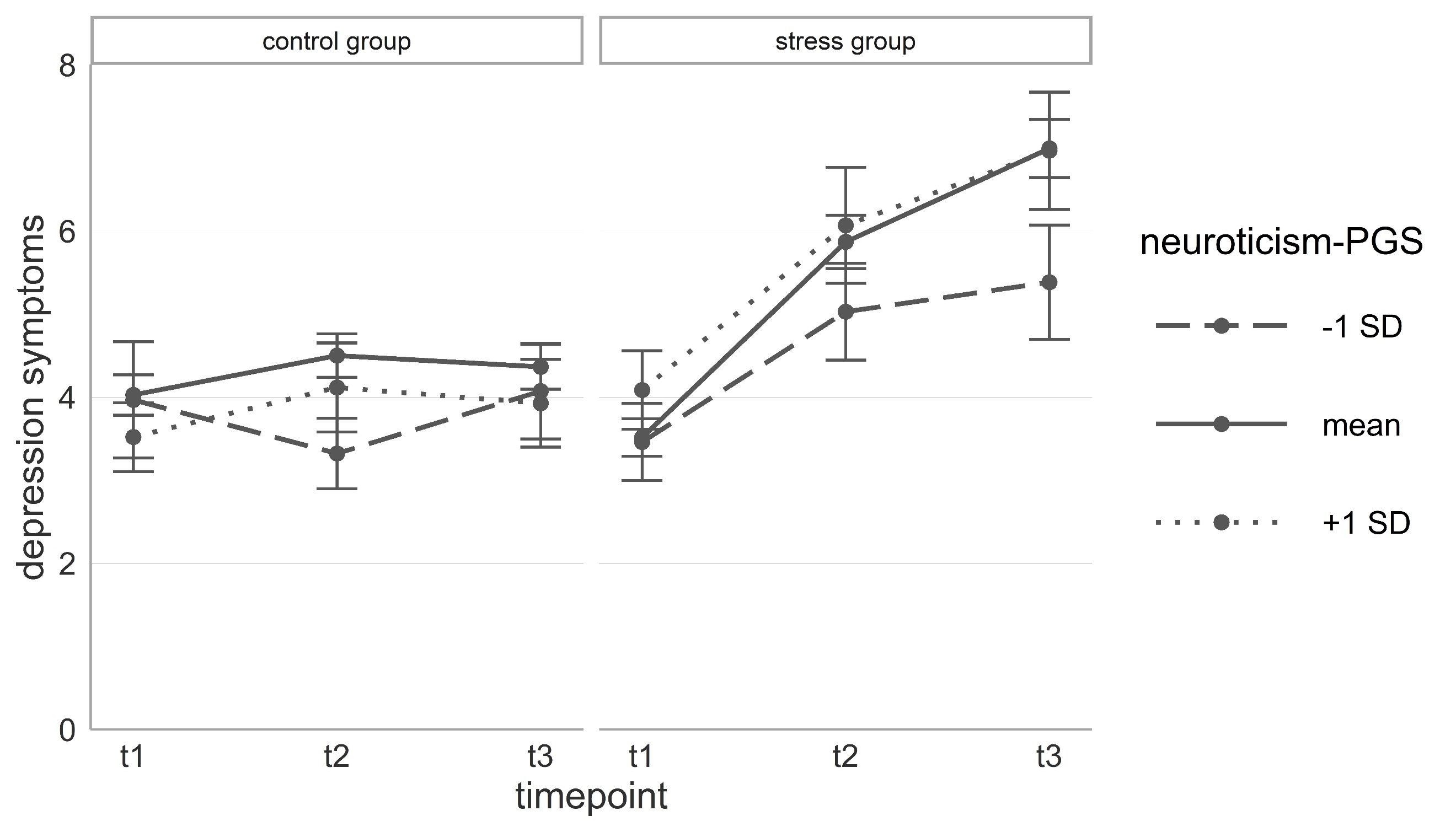


FIGURE S10. Time course of mean depression symptoms (± SEM) in the stress group (SG) and control group (CG) separated by polygenic score (PGS) for neuroticism (grouping based on SD for illustrative purposes only). *SD* = standard deviation.

# AUCg

TABLE S16. Parameter estimates for overall effects of the models with area under the curve with respect to the ground (*AUCg*) as dependent variable and the polygenic score for depression as predictor.

|  | **group.model^a^** | | |  | **DEP-PGS.model^b^** | | |  | **PC.model^c^** | | |
| --- | --- | --- | --- | --- | --- | --- | --- | --- | --- | --- | --- |
|  | Estimates | *SE* | *p* |  | Estimates | *SE* | *p* |  | Estimates | *SE* | *p* |
| Intercept | 549.93 | 19.66 | **< .001** |  | 548.32 | 19.64 | **< .001** |  | 554.05 | 23.06 | **< .001** |
| Timepoint | -7.62 | 5.40 | .158 |  | -7.67 | 5.41 | .157 |  | -8.77 | 5.48 | .110 |
| Women using HC (vs. other groups) | -77.64 | 21.73 | **< .001** |  | -76.61 | 21.68 | **.001** |  | -81.99 | 28.60 | **.005** |
| Men (vs. other groups) | -64.31 | 25.22 | **.012** |  | -60.03 | 25.31 | **.019** |  | -84.56 | 35.48 | **.018** |
| Awakening time | 27.02 | 12.81 | **.035** |  | 26.84 | 12.83 | **.037** |  | 49.79 | 16.29 | **.002** |
| SG (vs. CG) | 2.83 | 20.00 | .888 |  | 2.39 | 19.96 | .905 |  | -26.43 | 37.61 | .483 |
| Timepoint x SG | -19.15 | 7.89 | **.016** |  | -18.99 | 7.91 | **.017** |  | -15.43 | 8.12 | .058 |
| PGS |  |  |  |  | -13.55 | 14.02 | .335 |  | -21.53 | 23.54 | .362 |
| PGS x SG |  |  |  |  | 21.59 | 19.88 | .279 |  | 17.30 | 2.42 | .398 |
| PGS x timepoint |  |  |  |  | -3.24 | 5.41 | .550 |  | -3.25 | 5.49 | .554 |
| PGS x timepoint x SG |  |  |  |  | 2.43 | 7.83 | .757 |  | 2.17 | 7.90 | .784 |
| PC1 |  |  |  |  |  |  |  |  | -666.93 | 287.01 | **.021** |
| PC2 |  |  |  |  |  |  |  |  | 277.16 | 293.62 | .347 |
| PC3 |  |  |  |  |  |  |  |  | 175.17 | 291.36 | .549 |
| PC4 |  |  |  |  |  |  |  |  | -219.98 | 287.16 | .445 |
| PC5 |  |  |  |  |  |  |  |  | 135.19 | 291.06 | .643 |
| SG x PC1 |  |  |  |  |  |  |  |  | 516.60 | 406.07 | .205 |
| SG x PC2 |  |  |  |  |  |  |  |  | -33.33 | 414.27 | .426 |
| SG x PC3 |  |  |  |  |  |  |  |  | -504.76 | 422.89 | .234 |
| SG x PC4 |  |  |  |  |  |  |  |  | 231.68 | 448.82 | .606 |
| SG x PC5 |  |  |  |  |  |  |  |  | 215.34 | 416.93 | .606 |
| SG x Women using HC |  |  |  |  |  |  |  |  | 23.33 | 44.48 | .601 |
| SG x Men |  |  |  |  |  |  |  |  | 54.50 | 51.48 | .291 |
| SG x Awakening time |  |  |  |  |  |  |  |  | -62.65 | 26.99 | **.021** |
| PGS x PC1 |  |  |  |  |  |  |  |  | 255.85 | 192.94 | .187 |
| PGS x PC2 |  |  |  |  |  |  |  |  | -147.80 | 224.38 | .511 |
| PGS x PC3 |  |  |  |  |  |  |  |  | 305.52 | 212.00 | .151 |
| PGS x PC4 |  |  |  |  |  |  |  |  | 28.59 | 22.90 | .897 |
| PGS x PC5 |  |  |  |  |  |  |  |  | 27.10 | 229.29 | .906 |
| PGS x Women using HC |  |  |  |  |  |  |  |  | 11.65 | 25.97 | .654 |
| PGS x Men |  |  |  |  |  |  |  |  | 22.31 | 25.90 | .390 |
| PGS x Awakening time |  |  |  |  |  |  |  |  | 2.02 | 14.25 | .887 |
| AIC | 11879.83 | | |  | 11885.19 | | |  | 11907.09 | | |
| BIC | 11923.21 | | |  | 11947.85 | | |  | 12070.972 | | |
| Log-likelihood | -5930.91 | | |  | -5929.59 | | |  | -5919.55 | | |
| Observations | 916 | | |  | 916 | | |  | 916 | | |
| *n* | 196 | | |  | 196 | | |  | 196 | | |
| *SD*: participant (Intercept) | 105.40 | | |  | 104.48 | | |  | 99.42 | | |
| Marginal *R^2^* | 0.0553 | | |  | 0.0615 | | |  | 0.0972 | | |
| Conditional *R^2^* | 0.4083 | | |  | 0.4090 | | |  | 0.4110 | | |

Abbreviations: CG = Control group; HC = Hormonal contraception; PC = Principal component; PGS = Polygenic score; *SD* = Standard deviation; *SE* = Standard error; SG = Stress group.

^a^The group.model consists of a random intercept for participant and the fixed effects for the linear time trend, group and its interactions with the time trend as well as the covariates hormonal status and awakening time.

^b^The PGS.model includes the additional fixed effects PGS, PGS in interaction with group as well as with the linear time trend, and the three-way interaction of the PGS, group and the linear time trend.

^c^In the PC.model the covariates PC1 to 5 are added as well as the interactions PGS x covariates and group x covariates.

TABLE S17. Parameter estimates for overall effects of the models with area under the curve with respect to the ground (*AUCg*) as dependent variable and the polygenic score for neuroticism as predictor.

|  | **group.model^a^** | | |  | **NEU-PGS.model^b^** | | |  | **PC.model^c^** | | |
| --- | --- | --- | --- | --- | --- | --- | --- | --- | --- | --- | --- |
|  | Estimates | *SE* | *p* |  | Estimates | *SE* | *p* |  | Estimates | *SE* | *p* |
| Intercept | 549.93 | 19.66 | **< .001** |  | 547.89 | 19.08 | **< .001** |  | 564.73 | 21.48 | **< .001** |
| Timepoint | -7.62 | 5.40 | .158 |  | -7.66 | 5.41 | .157 |  | -10.53 | 5.48 | .055 |
| Women using HC (vs. other groups) | -77.64 | 21.73 | **< .001** |  | -75.86 | 21.01 | **< .001** |  | -95.17 | 26.68 | **.001** |
| Men (vs. other groups) | -64.31 | 25.22 | **.012** |  | -59.06 | 24.42 | **.017** |  | -89.51 | 33.72 | **.009** |
| Awakening time | 27.02 | 12.81 | **.035** |  | 27.47 | 12.89 | **.033** |  | 56.82 | 16.35 | **.001** |
| SG (vs. CG) | 2.83 | 20.00 | .888 |  | 2.39 | 19.47 | .903 |  | -35.20 | 36.14 | .331 |
| Timepoint x SG | -19.15 | 7.89 | **.016** |  | -19.25 | 7.91 | **.015** |  | -14.85 | 8.08 | .066 |
| PGS |  |  |  |  | -47.40 | 13.24 | **< .001** |  | -69.10 | 18.78 | **< .001** |
| PGS x SG |  |  |  |  | 48.23 | 19.36 | **.014** |  | 39.14 | 20.04 | .052 |
| PGS x timepoint |  |  |  |  | 2.67 | 5.28 | .614 |  | 4.18 | 5.31 | .432 |
| PGS x timepoint x SG |  |  |  |  | -4.91 | 7.96 | .538 |  | -2.81 | 8.07 | .728 |
| PC1 |  |  |  |  |  |  |  |  | -405.27 | 272.17 | .138 |
| PC2 |  |  |  |  |  |  |  |  | 336.43 | 277.98 | .228 |
| PC3 |  |  |  |  |  |  |  |  | 225.77 | 277.46 | .417 |
| PC4 |  |  |  |  |  |  |  |  | -316.83 | 269.78 | .242 |
| PC5 |  |  |  |  |  |  |  |  | 151.78 | 271.63 | .577 |
| SG x PC1 |  |  |  |  |  |  |  |  | 373.71 | 380.60 | .328 |
| SG x PC2 |  |  |  |  |  |  |  |  | -392.69 | 412.46 | .342 |
| SG x PC3 |  |  |  |  |  |  |  |  | -534.03 | 404.02 | .188 |
| SG x PC4 |  |  |  |  |  |  |  |  | 458.09 | 434.67 | .293 |
| SG x PC5 |  |  |  |  |  |  |  |  | 86.18 | 390.17 | .826 |
| SG x Women using HC |  |  |  |  |  |  |  |  | 40.95 | 42.42 | .336 |
| SG x Men |  |  |  |  |  |  |  |  | 66.61 | 49.77 | .183 |
| SG x Awakening time |  |  |  |  |  |  |  |  | -63.13 | 26.82 | **.019** |
| PGS x PC1 |  |  |  |  |  |  |  |  | -130.56 | 211.38 | .538 |
| PGS x PC2 |  |  |  |  |  |  |  |  | -129.46 | 204.28 | .527 |
| PGS x PC3 |  |  |  |  |  |  |  |  | -685.74 | 229.96 | **.003** |
| PGS x PC4 |  |  |  |  |  |  |  |  | -173.06 | 209.38 | .410 |
| PGS x PC5 |  |  |  |  |  |  |  |  | 492.26 | 232.26 | **.036** |
| PGS x Women using HC |  |  |  |  |  |  |  |  | 40.37 | 21.64 | .064 |
| PGS x Men |  |  |  |  |  |  |  |  | 21.83 | 23.55 | .355 |
| PGS x Awakening time |  |  |  |  |  |  |  |  | -37.50 | 13.20 | **.005** |
| AIC | 11879.83 | | |  | 11873.43 | | |  | 11878.76 | | |
| BIC | 11923.21 | | |  | 11936.09 | | |  | 12042.65 | | |
| Log-likelihood | -5930.91 | | |  | -5923.72 | | |  | -5905.38 | | |
| Observations | 916 | | |  | 916 | | |  | 916 | | |
| *n* | 196 | | |  | 196 | | |  | 196 | | |
| *SD*: participant (Intercept) | 105.40 | | |  | 100.13 | | |  | 92.45 | | |
| Marginal *R^2^* | 0.0553 | | |  | 0.0897 | | |  | 0.1465 | | |
| Conditional *R^2^* | 0.4083 | | |  | 0.4089 | | |  | 0.4191 | | |

Abbreviations: CG = Control group; HC = Hormonal contraception; PC = Principal component; PGS = Polygenic score; *SD* = Standard deviation; *SE* = Standard error; SG = Stress group.

^a^The group.model consists of a random intercept for participant and the fixed effects for the linear time trend, group and its interactions with the time trend as well as the covariates hormonal status and awakening time.

^b^The PGS.model includes the additional fixed effects PGS, PGS in interaction with group as well as with the linear time trend, and the three-way interaction of the PGS, group and the linear time trend.

^c^In the PC.model the covariates PC1 to 5 are added as well as the interactions PGS x covariates and group x covariates.

FIGURE S11. Time course of mean area under the curve with respect to the ground (*AUCg* ± SEM) in the stress group (SG) and control group (CG) until the exam at timepoint 4.


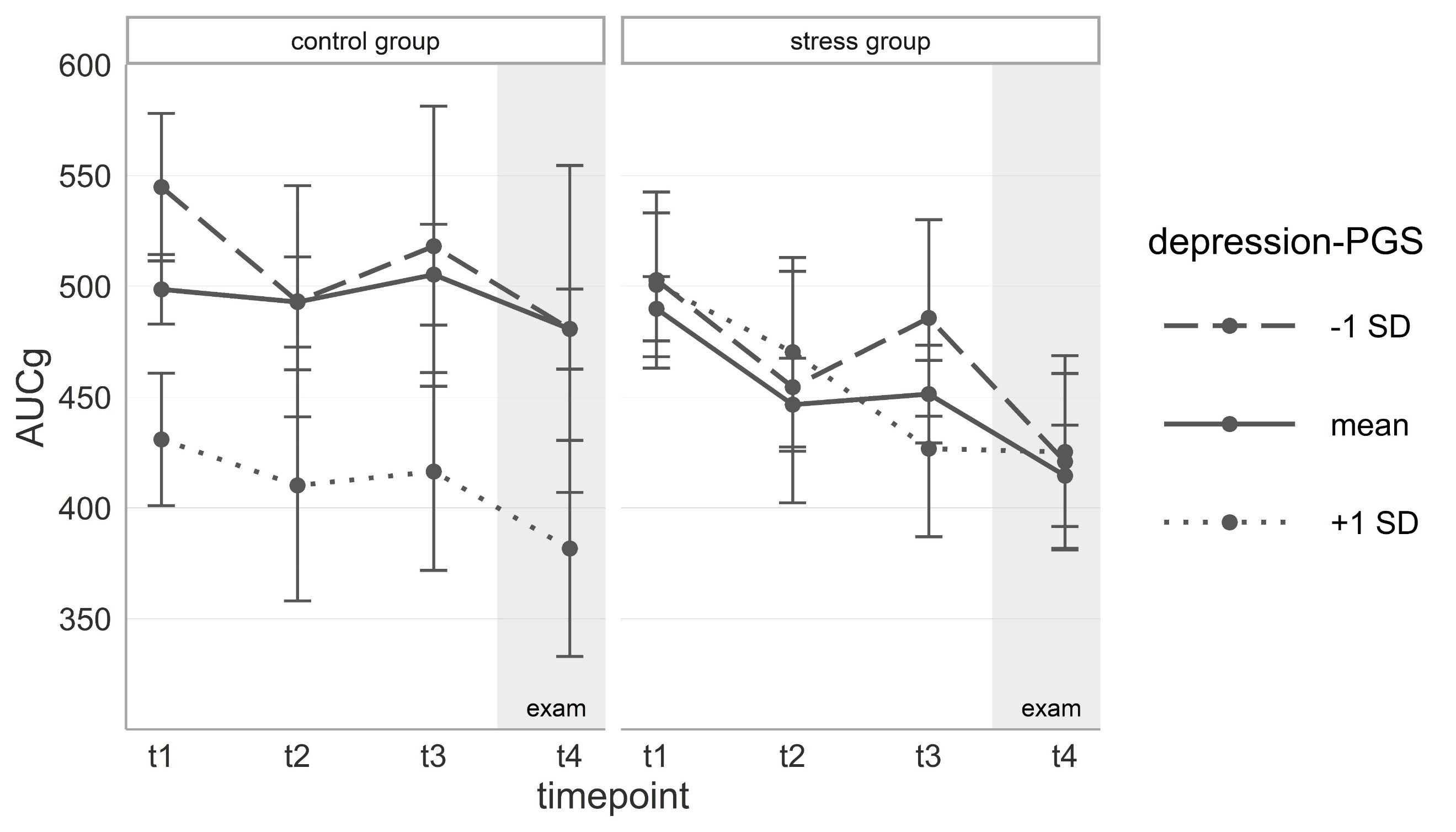

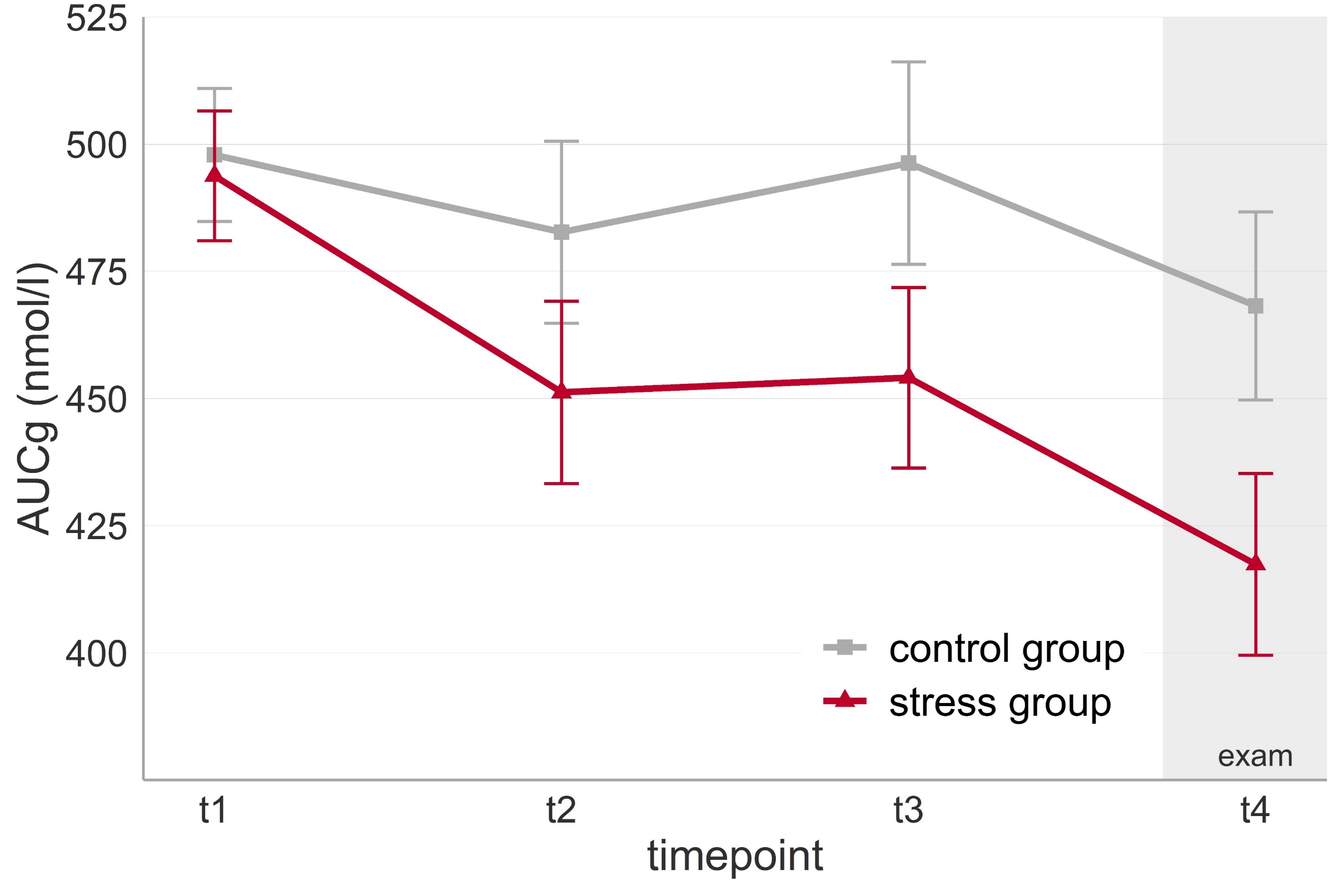


FIGURE S12. Time course of mean area under the curve with respect to the ground (*AUCg* ± SEM) in the stress group (SG) and control group (CG) separated by polygenic score (PGS) for depression (grouping based on SD for illustrative purposes only). *SD* = standard deviation.


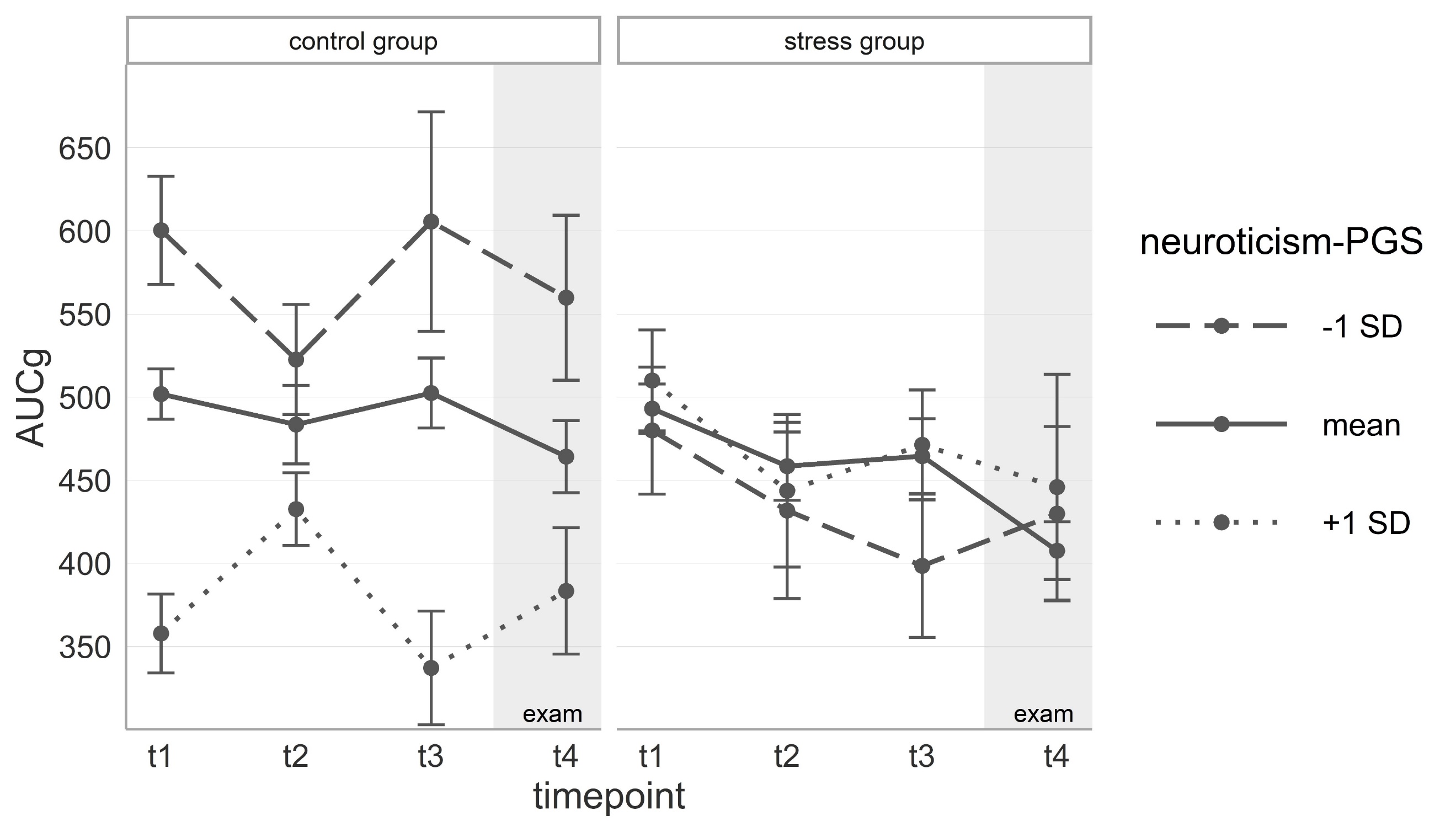


FIGURE S13. Time course of mean area under the curve with respect to the ground (*AUCg* ± SEM) in the stress group (SG) and control group (CG) separated by polygenic score (PGS) for neuroticism (grouping based on SD for illustrative purposes only). *SD* = standard deviation.

TABLE S18. Parameter estimates for overall effects of the models only containing the stress group (SG) with area under the curve with respect to the ground (*AUCg*) as dependent variable and the polygenic score for neuroticism as predictor.

|  | **SG.model^a^** | | |  | **NEU-PGS.model^b^** | | |  | **PC.model^c^** | | |
| --- | --- | --- | --- | --- | --- | --- | --- | --- | --- | --- | --- |
|  | Estimates | *SE* | *p* |  | Estimates | *SE* | *p* |  | Estimates | *SE* | *p* |
| Intercept | 534.47 | 27.39 | **< .001** |  | 534.45 | 27.45 | **< .001** |  | 536.95 | 26.17 | **< .001** |
| Timepoint | -24.28 | 5.95 | **< .001** |  | -24.43 | 5.97 | **< .001** |  | -25.94 | 6.01 | **< .001** |
| Women using HC (vs. other groups) | -64.75 | 3.99 | **.040** |  | -64.69 | 31.08 | **.040** |  | -59.01 | 29.25 | **.047** |
| Men (vs. other groups) | -34.11 | 34.05 | .319 |  | -34.09 | 34.13 | .321 |  | -29.37 | 32.92 | .375 |
| Awakening time | -12.77 | 21.39 | .551 |  | -13.09 | 21.45 | .542 |  | -3.28 | 21.79 | .880 |
| PGS |  |  |  |  | 1.49 | 13.29 | .911 |  | -33.84 | 24.97 | .179 |
| PGS x timepoint |  |  |  |  | -2.61 | 5.93 | .660 |  | 3.31 | 6.33 | .601 |
| PC1 |  |  |  |  |  |  |  |  | 39.72 | 267.28 | .882 |
| PC2 |  |  |  |  |  |  |  |  | 61.87 | 279.78 | .826 |
| PC3 |  |  |  |  |  |  |  |  | -401.26 | 255.14 | .120 |
| PC4 |  |  |  |  |  |  |  |  | 53.87 | 307.37 | .861 |
| PC5 |  |  |  |  |  |  |  |  | 229.65 | 249.24 | .360 |
| PGS x PC1 |  |  |  |  |  |  |  |  | 23.24 | 279.04 | .934 |
| PGS x PC2 |  |  |  |  |  |  |  |  | -13.17 | 301.77 | .965 |
| PGS x PC3 |  |  |  |  |  |  |  |  | -1071.02 | 297.72 | **.001** |
| PGS x PC4 |  |  |  |  |  |  |  |  | -605.72 | 334.10 | .074 |
| PGS x PC5 |  |  |  |  |  |  |  |  | 456.58 | 304.44 | .138 |
| PGS x Women using HC |  |  |  |  |  |  |  |  | 41.72 | 30.53 | .176 |
| PGS x Men |  |  |  |  |  |  |  |  | 15.25 | 31.15 | .626 |
| PGS x Awakening time |  |  |  |  |  |  |  |  | -56.65 | 20.47 | **.006** |
| AIC | 5527.23 | | |  | 5531.02 | | |  | 5527.42 | | |
| BIC | 5555.63 | | |  | 5567.53 | | |  | 5616.67 | | |
| Log-likelihood | -2756.62 | | |  | -2756.51 | | |  | -2741.71 | | |
| Observations | 427 | | |  | 427 | | |  | 427 | | |
| *n* | 95 | | |  | 95 | | |  | 95 | | |
| *SD*: participant (Intercept) | 89.86 | | |  | 89.87 | | |  | 74.11 | | |
| Marginal *R^2^* | 0.0530 | | |  | 0.0534 | | |  | 0.1539 | | |
| Conditional *R^2^* | 0.3384 | | |  | 0.3388 | | |  | 0.3489 | | |

Abbreviations: HC = Hormonal contraception; PC = Principal component; PGS = Polygenic score; *SD* = Standard deviation; *SE* = Standard error; SG = Stress group.

^a^The SG.model consists of a random intercept for participant and the fixed effects for the linear time trend and the covariates hormonal status and awakening time.

^b^The PGS.model includes the additional fixed effects PGS and PGS in interaction with the linear time trend.

^c^In the PC.model the covariates PC1 to 5 are added as well as the interactions PGS x covariates.

TABLE S19. Parameter estimates for overall effects of the models only containing the control group (CG) with area under the curve with respect to the ground (*AUCg*) as dependent variable and the polygenic score for neuroticism as predictor.

|  | **CG.model^a^** | | |  | **NEU-PGS.model^b^** | | |  | **PC.model^c^** | | |
| --- | --- | --- | --- | --- | --- | --- | --- | --- | --- | --- | --- |
|  | Estimates | *SE* | *p* |  | Estimates | *SE* | *p* |  | Estimates | *SE* | *p* |
| Intercept | 559.91 | 24.31 | **< .001** |  | 556.84 | 23.09 | **< .001** |  | 562.04 | 23.06 | **< .001** |
| Timepoint | -8.74 | 5.38 | .105 |  | -8.86 | 5.39 | .101 |  | -9.87 | 5.51 | .074 |
| Women using HC (vs. other groups) | -83.66 | 3.30 | **.007** |  | -81.17 | 28.65 | **.006** |  | -86.18 | 29.03 | **.004** |
| Men (vs. other groups) | -91.68 | 37.28 | **.016** |  | -81.52 | 35.35 | **.023** |  | -84.92 | 36.82 | **.024** |
| Awakening time | 49.60 | 15.91 | **.002** |  | 50.78 | 16.03 | **.002** |  | 54.74 | 16.47 | **.001** |
| PGS |  |  |  |  | -48.42 | 14.02 | **.001** |  | -6.19 | 24.15 | **.015** |
| PGS x timepoint |  |  |  |  | 3.59 | 5.32 | .501 |  | 4.07 | 5.40 | .451 |
| PC1 |  |  |  |  |  |  |  |  | -393.80 | 30.13 | .193 |
| PC2 |  |  |  |  |  |  |  |  | 435.14 | 305.98 | .159 |
| PC3 |  |  |  |  |  |  |  |  | 146.55 | 305.64 | .633 |
| PC4 |  |  |  |  |  |  |  |  | -281.49 | 294.36 | .342 |
| PC5 |  |  |  |  |  |  |  |  | 16.61 | 302.99 | .597 |
| PGS x PC1 |  |  |  |  |  |  |  |  | -185.95 | 344.04 | .590 |
| PGS x PC2 |  |  |  |  |  |  |  |  | -392.60 | 31.51 | .210 |
| PGS x PC3 |  |  |  |  |  |  |  |  | -156.61 | 376.24 | .678 |
| PGS x PC4 |  |  |  |  |  |  |  |  | 148.15 | 298.55 | .621 |
| PGS x PC5 |  |  |  |  |  |  |  |  | 546.46 | 372.33 | .146 |
| PGS x Women using HC |  |  |  |  |  |  |  |  | 22.73 | 32.05 | .480 |
| PGS x Men |  |  |  |  |  |  |  |  | 13.40 | 38.88 | .731 |
| PGS x Awakening time |  |  |  |  |  |  |  |  | -23.81 | 17.50 | .174 |
| AIC | 6352.37 | | |  | 6344.04 | | |  | 6359.28 | | |
| BIC | 6381.72 | | |  | 6381.78 | | |  | 6451.51 | | |
| Log-likelihood | -3169.19 | | |  | -3163.02 | | |  | -3157.64 | | |
| Observations | 489 | | |  | 489 | | |  | 489 | | |
| *n* | 101 | | |  | 101 | | |  | 101 | | |
| *SD*: participant (Intercept) | 116.41 | | |  | 107.88 | | |  | 101.80 | | |
| Marginal *R^2^* | 0.0599 | | |  | 0.1176 | | |  | 0.1581 | | |
| Conditional *R^2^* | 0.4609 | | |  | 0.4618 | | |  | 0.4646 | | |

Abbreviations: CG = Control group; HC = Hormonal contraception; PC = Principal component; PGS = Polygenic score; *SD* = Standard deviation; *SE* = Standard error.

^a^The CG.model consists of a random intercept for participant and the fixed effects for the linear time trend and the covariates hormonal status and awakening time.

^b^The PGS.model includes the additional fixed effects PGS and PGS in interaction with the linear time trend.

^c^In the PC.model the covariates PC1 to 5 are added as well as the interactions PGS x covariates.

TABLE S20. Parameter estimates for overall effects of the models with area under the curve with respect to the ground (*AUCg*) as dependent variable and the polygenic score continuous shrinkage (PGScs) for neuroticism as predictor.

|  | **group.model^a^** | | |  | **NEU-PGScs.model^b^** | | |  | **PC.model^c^** | | |
| --- | --- | --- | --- | --- | --- | --- | --- | --- | --- | --- | --- |
|  | Estimates | *SE* | *p* |  | Estimates | *SE* | *p* |  | Estimates | *SE* | *p* |
| Intercept | 549.93 | 19.66 | **< .001** |  | 550.04 | 18.99 | **< .001** |  | 562.14 | 21.89 | **< .001** |
| Timepoint | -7.62 | 5.40 | .158 |  | -7.55 | 5.41 | .163 |  | -9.19 | 5.48 | .094 |
| Women using HC (vs. other groups) | -77.64 | 21.73 | **< .001** |  | -78.60 | 2.92 | **< .001** |  | -94.16 | 27.38 | **.001** |
| Men (vs. other groups) | -64.31 | 25.22 | **.012** |  | -58.31 | 24.32 | **.017** |  | -70.37 | 34.60 | **.044** |
| Awakening time | 27.02 | 12.81 | **.035** |  | 27.66 | 12.84 | **.032** |  | 52.99 | 16.34 | **.001** |
| SG (vs. CG) | 2.83 | 20.00 | .888 |  | 1.14 | 19.40 | .953 |  | -27.20 | 36.66 | .459 |
| Timepoint x SG | -19.15 | 7.89 | **.016** |  | -18.83 | 7.91 | **.018** |  | -15.19 | 8.11 | .062 |
| PGS |  |  |  |  | -49.23 | 13.19 | **< .001** |  | -55.32 | 19.40 | **.005** |
| PGScs x SG |  |  |  |  | 38.26 | 19.35 | **.049** |  | 36.46 | 20.39 | .076 |
| PGScs x timepoint |  |  |  |  | 1.77 | 5.27 | .736 |  | 3.10 | 5.36 | .563 |
| PGScs x timepoint x SG |  |  |  |  | 4.32 | 7.90 | .585 |  | 3.60 | 7.97 | .651 |
| PC1 |  |  |  |  |  |  |  |  | -410.48 | 271.37 | .132 |
| PC2 |  |  |  |  |  |  |  |  | 454.47 | 288.12 | .117 |
| PC3 |  |  |  |  |  |  |  |  | 141.64 | 283.25 | .618 |
| PC4 |  |  |  |  |  |  |  |  | -306.86 | 278.24 | .272 |
| PC5 |  |  |  |  |  |  |  |  | 123.87 | 271.97 | .649 |
| SG x PC1 |  |  |  |  |  |  |  |  | 355.97 | 400.23 | .375 |
| SG x PC2 |  |  |  |  |  |  |  |  | -497.91 | 420.47 | .238 |
| SG x PC3 |  |  |  |  |  |  |  |  | -494.61 | 412.72 | .232 |
| SG x PC4 |  |  |  |  |  |  |  |  | 405.78 | 441.46 | .359 |
| SG x PC5 |  |  |  |  |  |  |  |  | 115.90 | 391.95 | .768 |
| SG x Women using HC |  |  |  |  |  |  |  |  | 31.26 | 43.45 | .473 |
| SG x Men |  |  |  |  |  |  |  |  | 39.32 | 50.42 | .437 |
| SG x Awakening time |  |  |  |  |  |  |  |  | -63.80 | 26.97 | **.018** |
| PGScs x PC1 |  |  |  |  |  |  |  |  | -125.98 | 218.70 | .565 |
| PGScs x PC2 |  |  |  |  |  |  |  |  | -75.01 | 217.19 | .730 |
| PGScs x PC3 |  |  |  |  |  |  |  |  | -305.74 | 214.47 | .156 |
| PGScs x PC4 |  |  |  |  |  |  |  |  | -261.48 | 217.90 | .232 |
| PGScs x PC5 |  |  |  |  |  |  |  |  | 59.57 | 218.56 | .786 |
| PGScs x Women using HC |  |  |  |  |  |  |  |  | 17.81 | 22.39 | .428 |
| PGScs x Men |  |  |  |  |  |  |  |  | 1.17 | 25.18 | .963 |
| PGScs x Awakening time |  |  |  |  |  |  |  |  | -18.19 | 12.75 | .154 |
| AIC | 11879.83 | | |  | 11870.65 | | |  | 11893.80 | | |
| BIC | 11923.21 | | |  | 11933.31 | | |  | 12057.68 | | |
| Log-likelihood | -5930.91 | | |  | -5922.33 | | |  | -5912.90 | | |
| Observations | 916 | | |  | 916 | | |  | 916 | | |
| *n* | 196 | | |  | 196 | | |  | 196 | | |
| *SD*: participant (Intercept) | 105.40 | | |  | 99.50 | | |  | 92.45 | | |
| Marginal *R^2^* | 0.0553 | | |  | 0.0947 | | |  | 0.1244 | | |
| Conditional *R^2^* | 0.4083 | | |  | 0.4098 | | |  | 0.4153 | | |

Abbreviations: CG = Control group; HC = Hormonal contraception; PC = Principal component; PGScs = Polygenic score continuous shrinkage; *SD* = Standard deviation; *SE* = Standard error; SG = Stress group.

^a^The group.model consists of a random intercept for participant and the fixed effects for the linear time trend, group and its interactions with the time trend as well as the covariates hormonal status and awakening time.

^b^The PGS.model includes the additional fixed effects PGS, PGScs in interaction with group as well as with the linear time trend, and the three-way interaction of the PGS, group and the linear time trend.

^c^In the PC.model the covariates PC1 to 5 are added as well as the interactions PGScs x covariates and group x covariates.

TABLE S21. Parameter estimates for overall effects of the models only containing the stress group (SG) with area under the curve with respect to the ground (*AUCg*) as dependent variable and the polygenic score continuous shrinkage (PGScs) for neuroticism as predictor.

|  | **SG.model^a^** | | |  | **NEU-PGScs.model^b^** | | |  | **PC.model^c^** | | |
| --- | --- | --- | --- | --- | --- | --- | --- | --- | --- | --- | --- |
|  | Estimates | *SE* | *p* |  | Estimates | *SE* | *p* |  | Estimates | *SE* | *p* |
| Intercept | 534.47 | 27.39 | **< .001** |  | 534.37 | 27.45 | **< .001** |  | 540.79 | 27.49 | **< .001** |
| Timepoint | -24.28 | 5.95 | **< .001** |  | -24.09 | 5.97 | **< .001** |  | -24.74 | 6.03 | **< .001** |
| Women using HC (vs. other groups) | -64.75 | 3.99 | **.040** |  | -64.61 | 31.10 | **.041** |  | -67.80 | 31.15 | **.033** |
| Men (vs. other groups) | -34.11 | 34.05 | .319 |  | -34.17 | 34.11 | .319 |  | -25.44 | 34.22 | .460 |
| Awakening time | -12.77 | 21.39 | .551 |  | -11.49 | 21.45 | .593 |  | -9.52 | 21.67 | .661 |
| PGS |  |  |  |  | -9.81 | 13.27 | .462 |  | -5.27 | 29.11 | .857 |
| PGScs x timepoint |  |  |  |  | 5.35 | 5.85 | .361 |  | 8.98 | 6.12 | .143 |
| PC1 |  |  |  |  |  |  |  |  | 22.18 | 291.24 | .940 |
| PC2 |  |  |  |  |  |  |  |  | 95.69 | 289.84 | .742 |
| PC3 |  |  |  |  |  |  |  |  | -474.31 | 278.90 | .093 |
| PC4 |  |  |  |  |  |  |  |  | 175.39 | 323.57 | .589 |
| PC5 |  |  |  |  |  |  |  |  | 196.56 | 256.38 | .446 |
| PGScs x PC1 |  |  |  |  |  |  |  |  | -378.99 | 282.25 | .183 |
| PGScs x PC2 |  |  |  |  |  |  |  |  | -290.98 | 325.54 | .374 |
| PGScs x PC3 |  |  |  |  |  |  |  |  | -744.15 | 330.72 | **.027** |
| PGScs x PC4 |  |  |  |  |  |  |  |  | -789.81 | 367.43 | **.035** |
| PGScs x PC5 |  |  |  |  |  |  |  |  | 13.19 | 272.28 | .962 |
| PGScs x Women using HC |  |  |  |  |  |  |  |  | -2.80 | 31.69 | .930 |
| PGScs x Men |  |  |  |  |  |  |  |  | -14.53 | 38.66 | .708 |
| PGScs x Awakening time |  |  |  |  |  |  |  |  | -45.82 | 21.12 | **.031** |
| AIC | 5527.23 | | |  | 5530.27 | | |  | 5538.78 | | |
| BIC | 5555.63 | | |  | 5566.78 | | |  | 5628.03 | | |
| Log-likelihood | -2756.62 | | |  | -2756.13 | | |  | -2747.39 | | |
| Observations | 427 | | |  | 427 | | |  | 427 | | |
| *n* | 95 | | |  | 95 | | |  | 95 | | |
| *SD*: participant (Intercept) | 89.86 | | |  | 89.82 | | |  | 80.44 | | |
| Marginal *R^2^* | 0.0530 | | |  | 0.0548 | | |  | 0.1158 | | |
| Conditional *R^2^* | 0.3384 | | |  | 0.3400 | | |  | 0.3454 | | |

Abbreviations: HC = Hormonal contraception; PC = Principal component; PGScs = Polygenic score; *SD* = Standard deviation continuous shrinkage; *SE* = Standard error; SG = Stress group.

^a^The SG.model consists of a random intercept for participant and the fixed effects for the linear time trend and the covariates hormonal status and awakening time.

^b^The PGS.model includes the additional fixed effects PGScs and PGScs in interaction with the linear time trend.

^c^In the PC.model the covariates PC1 to 5 are added as well as the interactions PGScs x covariates.

TABLE S22. Parameter estimates for overall effects of the models only containing the control group (CG) with area under the curve with respect to the ground (*AUCg*) as dependent variable and the polygenic score continuous shrinkage (PGScs) for neuroticism as predictor.

|  | **CG.model^a^** | | |  | **NEU-PGScs.model^b^** | | |  | **PC.model^c^** | | |
| --- | --- | --- | --- | --- | --- | --- | --- | --- | --- | --- | --- |
|  | Estimates | *SE* | *p* |  | Estimates | *SE* | *p* |  | Estimates | *SE* | *p* |
| Intercept | 559.91 | 24.31 | **< .001** |  | 558.73 | 22.92 | **< .001** |  | 559.11 | 22.97 | **< .001** |
| Timepoint | -8.74 | 5.38 | .105 |  | -8.69 | 5.39 | .108 |  | -8.64 | 5.48 | .116 |
| Women using HC (vs. other groups) | -83.66 | 3.30 | **.007** |  | -86.15 | 28.44 | **.003** |  | -87.39 | 29.18 | **.004** |
| Men (vs. other groups) | -91.68 | 37.28 | **.016** |  | -78.83 | 35.15 | **.027** |  | -69.25 | 37.00 | .065 |
| Awakening time | 49.60 | 15.91 | **.002** |  | 49.91 | 15.96 | **.002** |  | 50.23 | 16.37 | .002 |
| PGS |  |  |  |  | -49.82 | 14.01 | **.001** |  | -47.05 | 25.08 | .**064** |
| PGScs x timepoint |  |  |  |  | 2.21 | 5.33 | .679 |  | 2.23 | 5.49 | .685 |
| PC1 |  |  |  |  |  |  |  |  | -391.68 | 288.19 | .178 |
| PC2 |  |  |  |  |  |  |  |  | 427.88 | 309.09 | .170 |
| PC3 |  |  |  |  |  |  |  |  | 192.48 | 302.50 | .526 |
| PC4 |  |  |  |  |  |  |  |  | -237.01 | 298.18 | .429 |
| PC5 |  |  |  |  |  |  |  |  | 201.49 | 290.77 | .490 |
| PGScs x PC1 |  |  |  |  |  |  |  |  | 62.20 | 339.47 | .855 |
| PGScs x PC2 |  |  |  |  |  |  |  |  | -58.96 | 309.23 | .849 |
| PGScs x PC3 |  |  |  |  |  |  |  |  | 12.41 | 314.90 | .969 |
| PGScs x PC4 |  |  |  |  |  |  |  |  | -96.56 | 286.86 | .737 |
| PGScs x PC5 |  |  |  |  |  |  |  |  | 98.00 | 368.00 | .791 |
| PGScs x Women using HC |  |  |  |  |  |  |  |  | 14.72 | 33.46 | .661 |
| PGScs x Men |  |  |  |  |  |  |  |  | -34.78 | 39.18 | .377 |
| PGScs x Awakening time |  |  |  |  |  |  |  |  | -2.41 | 16.21 | .882 |
| AIC | 6352.37 | | |  | 6342.75 | | |  | 6362.13 | | |
| BIC | 6381.72 | | |  | 6380.48 | | |  | 6454.37 | | |
| Log-likelihood | -3169.19 | | |  | -3162.38 | | |  | -3159.07 | | |
| Observations | 489 | | |  | 489 | | |  | 489 | | |
| *n* | 101 | | |  | 101 | | |  | 101 | | |
| *SD*: participant (Intercept) | 116.41 | | |  | 106.86 | | |  | 102.30 | | |
| Marginal *R^2^* | 0.0599 | | |  | 0.1247 | | |  | 0.1528 | | |
| Conditional *R^2^* | 0.4609 | | |  | 0.4621 | | |  | 0.4621 | | |

Abbreviations: CG = Control group; HC = Hormonal contraception; PC = Principal component; PGScs = Polygenic score continuous shrinkage; *SD* = Standard deviation; *SE* = Standard error.

^a^The CG.model consists of a random intercept for participant and the fixed effects for the linear time trend and the covariates hormonal status and awakening time.

^b^The PGS.model includes the additional fixed effects PGScs and PGScs in interaction with the linear time trend.

^c^In the PC.model the covariates PC1 to 5 are added as well as the interactions PGScs x covariates.

# AUCi

TABLE S23. Parameter estimates for overall effects of the models with area under the curve with respect to the increase (*AUCi*) as dependent variable and the polygenic score for depression as predictor.

|  | **group.model^a^** | | |  | **DEP-PGS.model^b^** | | |  | **PC.model^c^** | | |
| --- | --- | --- | --- | --- | --- | --- | --- | --- | --- | --- | --- |
|  | Estimates | *SE* | *p* |  | Estimates | *SE* | *p* |  | Estimates | *SE* | *p* |
| Intercept | 228.30 | 14.50 | **< .001** |  | 227.94 | 14.55 | **< .001** |  | 234.24 | 16.29 | **< .001** |
| Timepoint | -35.13 | 16.11 | **.030** |  | -34.97 | 16.14 | **.031** |  | -33.40 | 16.40 | **.042** |
| Timepoint^2^ | 9.09 | 5.36 | .091 |  | 9.06 | 5.38 | .092 |  | 8.67 | 5.46 | .113 |
| Women using HC (vs. other groups) | -78.03 | 14.37 | **< .001** |  | -77.96 | 14.43 | **< .001** |  | -83.90 | 18.53 | **< .001** |
| Men (vs. other groups) | -75.75 | 16.77 | **< .001** |  | -75.25 | 16.94 | **< .001** |  | -86.67 | 22.95 | **< .001** |
| Awakening time | -38.44 | 11.76 | **.001** |  | -38.04 | 11.79 | **.001** |  | -42.00 | 14.74 | **.005** |
| SG (vs. CG) | -10.60 | 16.11 | .511 |  | -10.34 | 16.13 | .523 |  | -26.54 | 26.20 | .313 |
| Timepoint x SG | 38.46 | 24.14 | .112 |  | 38.31 | 24.20 | .114 |  | 39.90 | 24.68 | .106 |
| Timepoint^2^ x SG | -19.81 | 8.12 | **.015** |  | -19.80 | 8.14 | **.015** |  | -2.65 | 8.39 | **.014** |
| PGS |  |  |  |  | -13.12 | 11.33 | .249 |  | -2.41 | 16.63 | .885 |
| PGS x SG |  |  |  |  | 10.46 | 16.08 | .516 |  | 1.64 | 16.18 | .511 |
| PGS x timepoint |  |  |  |  | 20.60 | 16.28 | .206 |  | 19.27 | 16.53 | .244 |
| PGS x timepoint^2^ |  |  |  |  | -4.89 | 5.42 | .368 |  | -4.59 | 5.50 | .404 |
| PGS x timepoint x SG |  |  |  |  | -19.18 | 23.59 | .416 |  | -16.11 | 24.44 | .510 |
| PGS x timepoint^2^ x SG |  |  |  |  | 4.87 | 7.87 | .536 |  | 3.83 | 8.20 | .641 |
| PC1 |  |  |  |  |  |  |  |  | 209.04 | 185.81 | .262 |
| PC2 |  |  |  |  |  |  |  |  | 342.64 | 191.00 | .075 |
| PC3 |  |  |  |  |  |  |  |  | 253.13 | 189.11 | .183 |
| PC4 |  |  |  |  |  |  |  |  | -369.97 | 186.22 | **.049** |
| PC5 |  |  |  |  |  |  |  |  | 74.67 | 188.98 | .693 |
| SG x PC1 |  |  |  |  |  |  |  |  | -568.76 | 264.72 | **.033** |
| SG x PC2 |  |  |  |  |  |  |  |  | -39.98 | 269.58 | .149 |
| SG x PC3 |  |  |  |  |  |  |  |  | -663.40 | 276.24 | **.017** |
| SG x PC4 |  |  |  |  |  |  |  |  | 365.25 | 295.42 | .218 |
| SG x PC5 |  |  |  |  |  |  |  |  | 25.45 | 27.82 | .356 |
| SG x Women using HC |  |  |  |  |  |  |  |  | 18.50 | 28.95 | .524 |
| SG x Men |  |  |  |  |  |  |  |  | 31.25 | 33.60 | .354 |
| SG x Awakening time |  |  |  |  |  |  |  |  | 12.56 | 25.29 | .620 |
| PGS x PC1 |  |  |  |  |  |  |  |  | -209.74 | 125.12 | .096 |
| PGS x PC2 |  |  |  |  |  |  |  |  | -139.95 | 145.98 | .339 |
| PGS x PC3 |  |  |  |  |  |  |  |  | -73.42 | 137.86 | .595 |
| PGS x PC4 |  |  |  |  |  |  |  |  | -57.99 | 143.43 | .687 |
| PGS x PC5 |  |  |  |  |  |  |  |  | -141.80 | 149.94 | .346 |
| PGS x Women using HC |  |  |  |  |  |  |  |  | -4.13 | 16.88 | .807 |
| PGS x Men |  |  |  |  |  |  |  |  | -21.68 | 16.86 | .200 |
| PGS x Awakening time |  |  |  |  |  |  |  |  | 6.05 | 13.24 | .648 |
| AIC | 11527.59 | | |  | 11537.17 | | |  | 11553.99 | | |
| BIC | 11590.25 | | |  | 11628.75 | | |  | 11746.79 | | |
| Log-likelihood | -5750.80 | | |  | -5749.58 | | |  | -5737.00 | | |
| Observations | 916 | | |  | 916 | | |  | 916 | | |
| *n* | 196 | | |  | 196 | | |  | 196 | | |
| *SD*: participant (Intercept) | 78.30 | | |  | 77.99 | | |  | 71.94 | | |
| *SD*: timepoint | 34.39 | | |  | 34.08 | | |  | 33.58 | | |
| Correlation: participant x timepoint | -.61 | | |  | -.61 | | |  | -.64 | | |
| Marginal *R^2^* | 0.1118 | | |  | 0.1143 | | |  | 0.1535 | | |
| Conditional *R^2^* | 0.3843 | | |  | 0.3853 | | |  | 0.3844 | | |

Abbreviations: CG = Control group; HC = Hormonal contraception; PC = Principal component; PGS = Polygenic score; *SD* = Standard deviation; *SE* = Standard error; SG = Stress group.

^a^The group.model consists of a random intercept for participant, a random slope for the linear time trend and the fixed effects for the linear and quadratic time trend, group and its interactions with the time trends as well as the covariates hormonal status and awakening time.

^b^The PGS.model includes the additional fixed effects PGS, PGS in interaction with group as well as with the linear and quadratic time trend, and the three-way interaction of the PGS, group and the time trends.

^c^In the PC.model the covariates PC1 to 5 are added as well as the interactions PGS x covariates and group x covariates.

|  | **group.model^a^** | | |  | **NEU-PGS.model^b^** | | |  | **PC.model^c^** | | |
| --- | --- | --- | --- | --- | --- | --- | --- | --- | --- | --- | --- |
|  | Estimates | *SE* | *p* |  | Estimates | *SE* | *p* |  | Estimates | *SE* | *p* |
| Intercept | 228.30 | 14.50 | **< .001** |  | 227.68 | 14.41 | **< .001** |  | 232.74 | 15.70 | **< .001** |
| Timepoint | -35.13 | 16.11 | **.030** |  | -35.13 | 16.12 | **.030** |  | -34.02 | 16.38 | **.038** |
| Timepoint^2^ | 9.09 | 5.36 | .091 |  | 9.08 | 5.37 | .091 |  | 8.76 | 5.44 | .108 |
| Women using HC (vs. other groups) | -78.03 | 14.37 | **< .001** |  | -77.61 | 14.27 | **< .001** |  | -84.47 | 17.98 | **< .001** |
| Men (vs. other groups) | -75.75 | 16.77 | **< .001** |  | -73.98 | 16.68 | **< .001** |  | -82.54 | 22.69 | **< .001** |
| Awakening time | -38.44 | 11.76 | **.001** |  | -38.04 | 11.81 | **.001** |  | -42.42 | 14.90 | **.005** |
| SG (vs. CG) | -10.60 | 16.11 | .511 |  | -10.75 | 16.01 | .503 |  | -3.61 | 26.00 | .241 |
| Timepoint x SG | 38.46 | 24.14 | .112 |  | 38.23 | 24.18 | .114 |  | 41.85 | 24.66 | .090 |
| Timepoint^2^ x SG | -19.81 | 8.12 | **.015** |  | -19.65 | 8.13 | **.016** |  | -21.15 | 8.38 | **.012** |
| PGS |  |  |  |  | -25.33 | 1.91 | **.021** |  | -3.97 | 14.01 | **.029** |
| PGS x SG |  |  |  |  | 28.93 | 15.94 | .071 |  | 26.11 | 16.02 | .105 |
| PGS x timepoint |  |  |  |  | 28.63 | 15.69 | .068 |  | 29.04 | 16.15 | .073 |
| PGS x timepoint^2^ |  |  |  |  | -8.82 | 5.23 | .092 |  | -9.07 | 5.38 | .092 |
| PGS x timepoint x SG |  |  |  |  | -36.28 | 23.72 | .127 |  | -35.24 | 24.78 | .156 |
| PGS x timepoint^2^ x SG |  |  |  |  | 12.09 | 7.95 | .129 |  | 11.95 | 8.45 | .157 |
| PC1 |  |  |  |  |  |  |  |  | 174.08 | 183.35 | .344 |
| PC2 |  |  |  |  |  |  |  |  | 26.55 | 187.59 | .167 |
| PC3 |  |  |  |  |  |  |  |  | 193.25 | 187.53 | .304 |
| PC4 |  |  |  |  |  |  |  |  | -279.55 | 182.00 | .126 |
| PC5 |  |  |  |  |  |  |  |  | 38.88 | 183.47 | .832 |
| SG x PC1 |  |  |  |  |  |  |  |  | -642.93 | 257.98 | **.014** |
| SG x PC2 |  |  |  |  |  |  |  |  | -354.52 | 279.06 | .206 |
| SG x PC3 |  |  |  |  |  |  |  |  | -567.35 | 274.63 | **.040** |
| SG x PC4 |  |  |  |  |  |  |  |  | 36.87 | 296.64 | .226 |
| SG x PC5 |  |  |  |  |  |  |  |  | 274.67 | 263.96 | .300 |
| SG x Women using HC |  |  |  |  |  |  |  |  | 22.02 | 28.73 | .444 |
| SG x Men |  |  |  |  |  |  |  |  | 33.51 | 33.77 | .323 |
| SG x Awakening time |  |  |  |  |  |  |  |  | 15.07 | 25.19 | .550 |
| PGS x PC1 |  |  |  |  |  |  |  |  | -211.04 | 144.97 | .147 |
| PGS x PC2 |  |  |  |  |  |  |  |  | -85.61 | 137.93 | .536 |
| PGS x PC3 |  |  |  |  |  |  |  |  | -261.10 | 156.34 | .097 |
| PGS x PC4 |  |  |  |  |  |  |  |  | 35.00 | 141.75 | .805 |
| PGS x PC5 |  |  |  |  |  |  |  |  | 112.52 | 157.33 | .476 |
| PGS x Women using HC |  |  |  |  |  |  |  |  | 1.73 | 14.66 | .906 |
| PGS x Men |  |  |  |  |  |  |  |  | 18.26 | 15.96 | .254 |
| PGS x Awakening time |  |  |  |  |  |  |  |  | -2.40 | 12.35 | .846 |
| AIC | 11527.59 | | |  | 11531.84 | | |  | 11546.35 | | |
| BIC | 11590.25 | | |  | 11623.42 | | |  | 11739.15 | | |
| Log-likelihood | -5750.80 | | |  | -5746.92 | | |  | -5733.18 | | |
| Observations | 916 | | |  | 916 | | |  | 916 | | |
| *n* | 196 | | |  | 196 | | |  | 196 | | |
| *SD*: participant (Intercept) | 78.30 | | |  | 77.07 | | |  | 68.28 | | |
| *SD*: timepoint | 34.39 | | |  | 34.44 | | |  | 33.83 | | |
| Correlation: participant x timepoint | -.61 | | |  | -.61 | | |  | -.60 | | |
| Marginal *R^2^* | 0.1118 | | |  | 0.1217 | | |  | 0.1642 | | |
| Conditional *R^2^* | 0.3843 | | |  | 0.3881 | | |  | 0.3879 | | |

TABLE S24. Parameter estimates for overall effects of the models with area under the curve with respect to the increase (*AUCi*) as dependent variable and the polygenic score for neuroticism as predictor.

Abbreviations: CG = Control group; PC = Principal component; PGS = Polygenic score; *SD* = Standard deviation; *SE* = Standard error; SG = Stress group.

^a^The group.model consists of a random intercept for participant, a random slope for the linear time trend and the fixed effects for the linear and quadratic time trend, group and its interactions with the time trends as well as the covariates hormonal status and awakening time.

^b^The PGS.model includes the additional fixed effects PGS, PGS in interaction with group as well as with the linear and quadratic time trend, and the three-way interaction of the PGS, group and the time trends.

^c^In the PC.model the covariates PC1 to 5 are added as well as the interactions PGS x covariates and group x covariates.


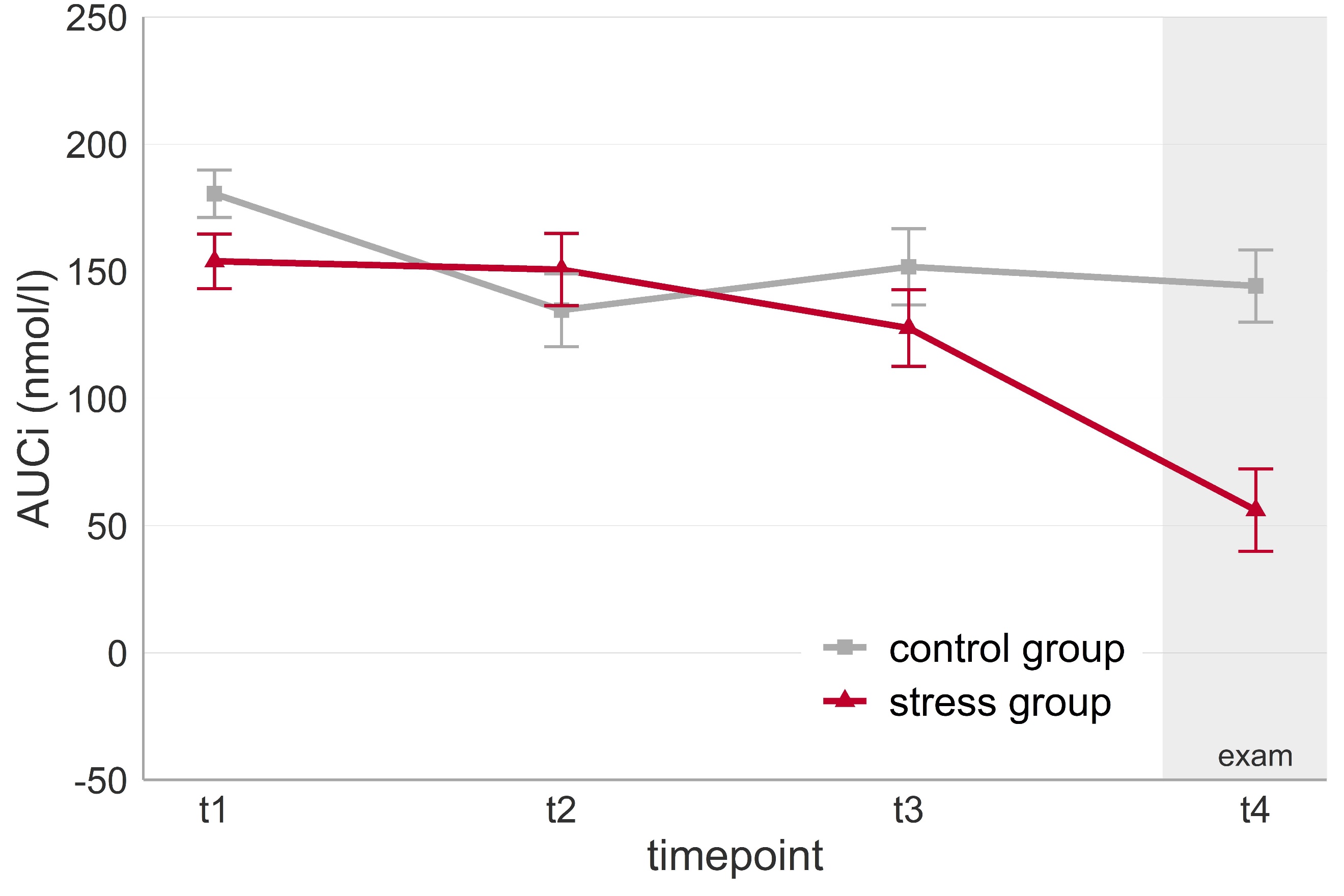


FIGURE S14. Time course of mean area under the curve with respect to the increase (*AUCi* ± SEM) in the stress group (SG) and control group (CG) until the exam at timepoint 4.


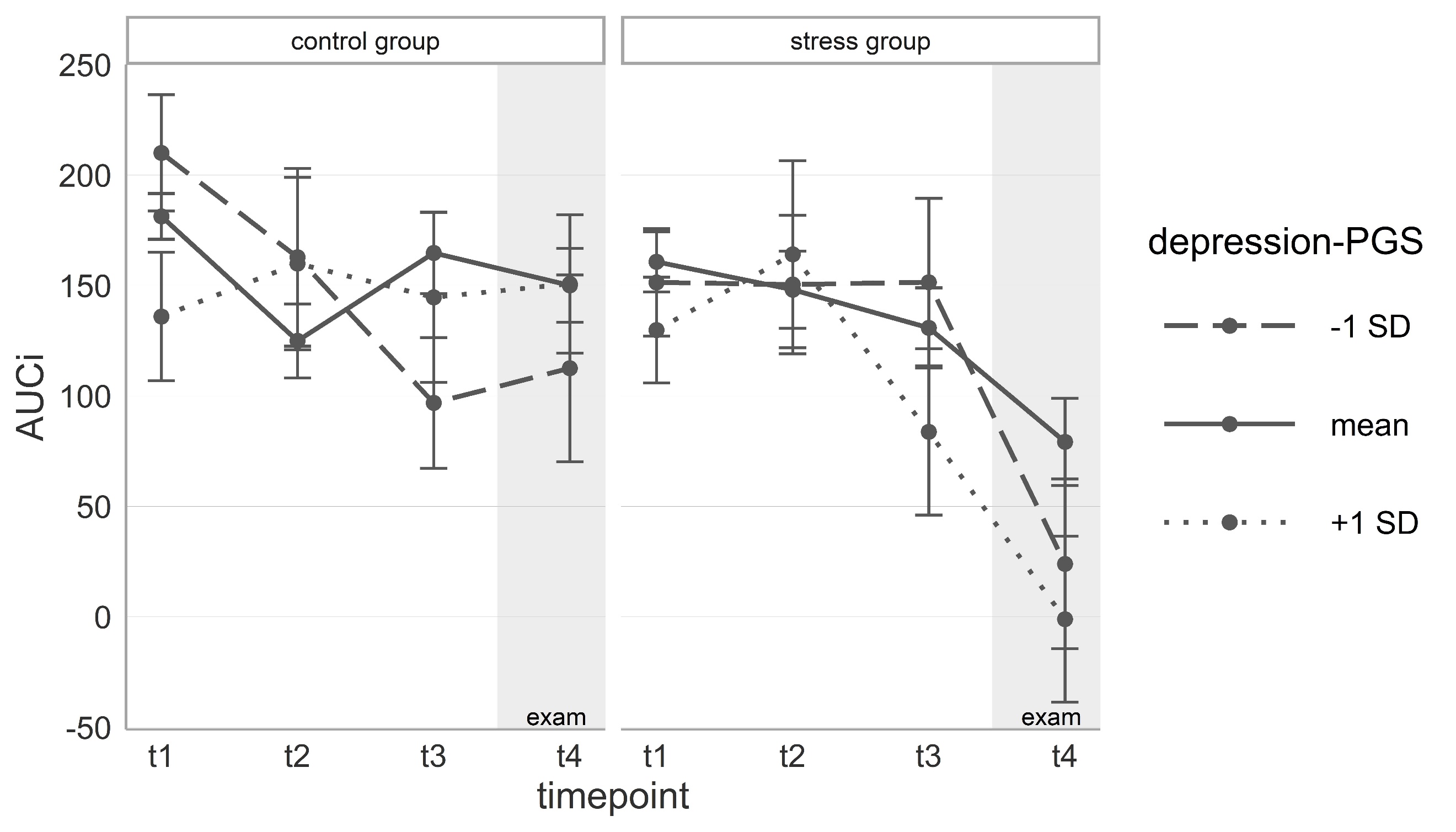


FIGURE S15. Time course of mean area under the curve with respect to the increase (*AUCi* ± SEM) in the stress group (SG) and control group (CG) separated by polygenic score (PGS) for depression (grouping based on SD for illustrative purposes only). *SD* = standard deviation.


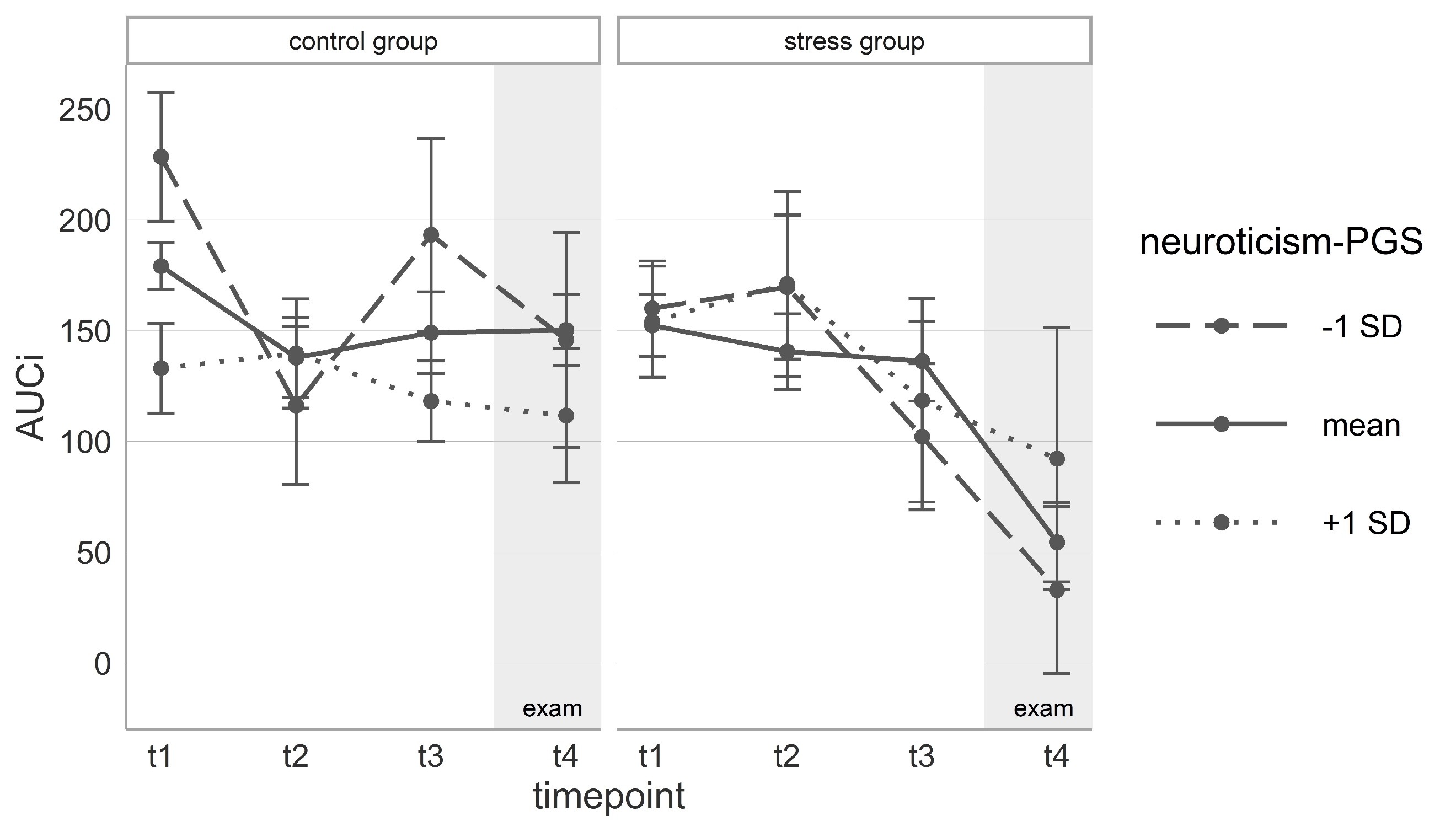


FIGURE S16. Time course of mean area under the curve with respect to the increase (*AUCi* ± SEM) in the stress group (SG) and control group (CG) separated by polygenic score (PGS) for neuroticism (grouping based on SD for illustrative purposes only). *SD* = standard deviation.

# Supplemental References

1. Giglberger M, Peter HL, Kraus E, et al. Daily life stress and the cortisol awakening response over a 13-months stress period - findings from the LawSTRESS project. *Psychoneuroendocrinology.* 2022;141. <https://doi.org/10.1016/j.psyneuen.2022.105771>
